# Single‐Cell Transcriptomic Analysis Identifies a Novel OLR1 ^+^ SLC7A7 ^+^ Liver‐Enriched Metastatic Subset With Immunometabolic Rewiring in Pancreatic Cancer

**DOI:** 10.1002/cam4.71345

**Published:** 2025-11-02

**Authors:** Xuan Yang, Xinyuan Chen, Zixin Wang, Yanfang Liu, Huiting Hu, Qingru Wu, Hailing Zhang, Yu Xiong, Xin Li, Xiaotao Cheng, Xiaoyu Ruan, Yan Gu

**Affiliations:** ^1^ National key Laboratory of Immunity and Inflammation, Institute of Immunology Naval Medical University Shanghai China; ^2^ Department of Pathology, Changhai Hospital Naval Medical University Shanghai China; ^3^ Department of Neurology, Changhai Hospital Naval Medical University Shanghai China; ^4^ AisenseBio Biomedical Technology Co., Ltd. JiangSu China

**Keywords:** immunosuppression, liver metastases, macrophages, metabolic reprogramming, pancreatic ductal adenocarcinoma

## Abstract

**Background:**

Pancreatic ductal adenocarcinoma (PDAC) is an exceptionally lethal malignancy, with high percents of patients presenting with liver metastases (LM). However, the mechanisms driving liver metastases remain critical bottlenecks requiring urgent exploration.

**Objective:**

To identify the key cellular subsets driving PDAC liver metastases, elucidate their interactions with the metastatic microenvironment, and define the underlying mechanisms of liver colonization.

**Materials and Methods:**

Integrated single‐cell transcriptomic analysis was performed using scRNA‐seq data of PT and LM. The expression of signature genes within the identified cell subset was validated using clinical samples from PDAC PT and LM patients. Furthermore, ligand‐receptor network analysis was conducted between the specific tumor cell subset and key immune cells.

**Results:**

We identified a novel liver‐enriched metastatic subset (LEMS), a terminally differentiated malignant cell subpopulation characterized by metabolic reprogramming and hyperactivation of immunosuppressive pathways. We further validated the LEMS signature genes, oxidized low‐density lipoprotein receptor 1 (OLR1) and solute carrier family 7 member 7 (SLC7A7), as potential diagnostic biomarkers for liver metastases. Importantly, we found that SPP1^+^ macrophages interacted with LEMS via ligand‐receptor networks, thereby driving invasion and immune evasion.

**Discussion:**

We revealed the highly malignant features of LEMS and crosstalk between LEMS and SPP1^+^ macrophages in liver metastases. However, it is necessary to expand clinical cohorts and in vivo models to comprehensively elucidate the specific mechanistic interactions between LEMS and macrophages.

**Conclusion:**

We delineated LEMS as an enriched subset in LM and proposed targeting of LEMS‐SPP1^+^ macrophage interactions as a therapeutic strategy to disrupt metastatic progression.

## 1 Introduction

Pancreatic cancer is a particularly aggressive solid malignancy with an increasing incidence rate annually. It is characterized by a dismal prognosis, with an overall 5‐year survival rate of approximately 10%, among the lowest of all cancers [1, 2]. Most pancreatic neoplasms present as pancreatic ductal adenocarcinoma (PDAC), in which invasion and metastases are critical factors contributing to its high mortality. More than half of patients present with systemic metastases at diagnosis, with the liver serving as the primary site of distant metastases [3]. The process of metastases is multifaceted, encompassing genetic alterations, intricate biological mechanisms, and dynamic interactions with components of the tumor microenvironment (TME) [4].

The development and metastases of PDAC are driven by crucial gene mutations and dysregulation of key signaling pathways. Notably, KRAS mutation is present in 90% of PDAC patients [5]. Elevated levels of the oncogenic KRAS gene and its aberrant signaling pathway lead to diverse tumor cell phenotypic, differentiation, and metastases, contributing to poor clinical outcomes [6]. Common oncogenic mutations in PDAC are enriched in 13 key signaling pathways, including RAS, TGF‐β, JNK, integrin, WNT‐Notch, and Hedgehog [7]. However, the precise mechanisms by which genetic mutations coordinate with metabolic and immune reprogramming to enable metastatic spread remain poorly understood. Elucidating these mechanisms is critical for identifying therapeutic vulnerabilities and improving outcomes in PDAC.

The metabolic abnormalities of tumor cells are one of the leading causes of their heterogeneity. One of the important hallmarks of cancer is metabolic reprogramming, which provides a steady supply of energy and biosynthetic precursors to fuel tumor progression. It can improve the capability of malignant cells to adapt to an adverse microenvironment and facilitate immune evasion [8]. These metabolic alterations, characterized by aerobic glycolysis, modified lipid metabolism, and glutaminolysis, not only vary among different cancer types but also show significant variation between primary tumors and their metastatic counterparts [9, 10]. In PDAC, the metabolic change of oxidative phosphorylation switching to glycolysis enhances epithelial‐mesenchymal transition (EMT), angiogenesis and tumor cell colonization of distant organs, facilitating the invasion–metastasis cascade [11]. Under metabolic stress, cancer cells integrate lipolysis and lipogenesis to form a fatty acid cycling network that sustains rapid tumor growth, proliferation, and invasive metastases [12]. Therefore, elucidating the metabolic alterations and regulatory mechanisms in primary and metastatic tumor cells is of paramount importance to uncover the metastatic process.

In PDAC, liver metastases exhibit pronounced immune exclusion, characterized by decreased cytotoxic CD8^+^ T cells, enriched regulatory T cells (Tregs) [13, 14], and tumor‐associated macrophages (TAMs) polarized toward immunosuppressive phenotypes [15]. Secreted phosphoprotein 1 (SPP1)^+^ TAMs, in particular, drive fibrosis, angiogenesis, and T cell exhaustion, fostering a sanctuary for metastatic cells [16, 17]. Moreover, myeloid‐derived suppressor cells (MDSCs) and neutrophil extracellular traps (NETs) further amplify immune suppression by impairing natural killer (NK) cell function and promoting pre‐metastatic niche formation [18, 19, 20]. These immunosuppressive mechanisms are exacerbated by metabolic crosstalk: lactate from glycolytic tumor cells inhibits dendritic cell maturation, while arginase‐expressing myeloid cells deplete extracellular arginine, crippling T cell receptor signaling [21, 22, 23]. However, how PDAC malignant cell subsets directly orchestrate immune evasion in liver metastases remains poorly understood, particularly through ligand‐receptor networks that dynamically reshape the metastatic niche.

In this study, we analyzed available single‐cell sequencing data from PDAC PT and LM obtained from the GSE205013 and GSE197177 databases. Subsequently, we identified a specific subset of malignant cells enriched in LM, which we designated as the liver‐enriched metastatic subset (LEMS). The LEMS is a terminally differentiated malignant cell subset with immunometabolic rewiring to fuel pancreatic cancer liver metastases.

## 2 Results

### 2.1 Single‐Cell Transcription Atlas and Cell Typing in PT and LM

**FIGURE 1:**
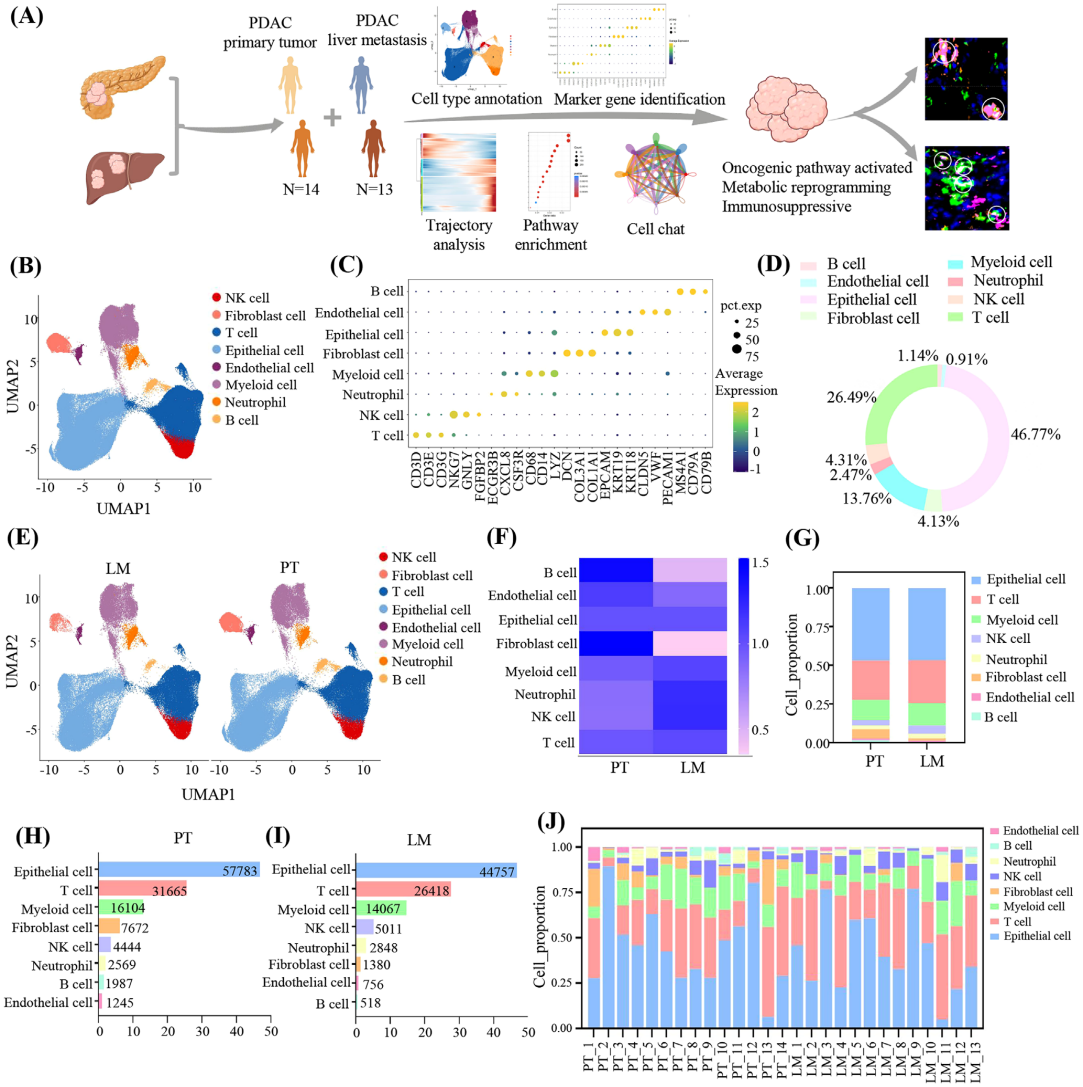
Analysis of distinct cellular constitutions between PT and LM through integrated single cell transcriptomic data. (A) Flowchart of the overall experimental design and analysis. (B) Uniform manifold approximation and projection (UMAP) plot of 219,224 cells showing the clustering of different cell types. (C) Bubble plot showing canonical marker genes in eight cell types. The size represents the proportion of cells expressing the genes and the color represents mean expression levels of the genes. (D) The percentage of each cell type. (E) UMAP plot displaying cell distribution, categorized by PT and LM. (F) The preference of each cell type across different groups estimated by Ro/e score. (G) Bar plot displaying relative proportion of cell subpopulations between PT (left) and LM (right). (H, I) The abundance and proportions of eight cell types ranked in descending order in PT (H) and LM (I). (J) Stacked plot showing the proportion of various cell types contributed to each sample.

To investigate the mechanisms underlying the high propensity of pancreatic cancer to metastasize to the liver, we used the Harmony algorithm to integrate available single‐cell RNA sequencing (scRNA‐seq) data from previous studies by Gregor Werba et al. [24] and Shu Zhang et al. [25], which can effectively remove batch effects (Figure S1A,B). We generated high‐precision scRNA‐seq profiles from 14 PT and 13 LM samples, retaining 219,224 cells after stringent filtration and normalization (Figure 1A). Among these, 123,469 cells were from PT and 95,755 from LM. Unsupervised clustering analysis and Uniform Manifold Approximation and Projection (UMAP) were used for dimensionality reduction and visualization. Based on well‐known marker genes, cells were classified into eight major types (Figure 1B,C). Epithelial cells and T cells were the most abundant, followed by myeloid cells (Figure 1D).

We further analyzed the single‐cell transcriptome atlases for PT and LM separately to show the compositional changes (Figure 1E). The integrated cellular atlas demonstrated a remarkable concordance with the respective tissue subgroup mappings (Figure 1G–I). The ratio of observed over expected cell numbers (Ro/e) analysis revealed an accumulation of B cells and fibroblasts in PT compared to LM (Figure 1F). Fibroblasts, particularly cancer‐associated fibroblasts (CAFs), are prevalent in the PDAC microenvironment and drive tumor progression through extracellular matrix production and cytokine secretion [26, 27]. In contrast, neutrophils and NK cells were enriched in LM compared to PT (Figure 1F). Neutrophils significantly contribute to the establishment of the immunosuppressive microenvironment in LM of PDAC, with P2RX1‐negative neutrophils promoting metabolic reprogramming and antitumor immunity evasion [28]. Although all cell subtypes were present in each lesion, their proportions varied significantly among individuals, indicating both lesion consistency and inter‐individual heterogeneity (Figure 1J). Thus, we analyzed single‐cell transcriptomic data to show the cell typing in the primary tumors and liver metastatic lesions of PDAC.

### 2.2 Identification of a Special Subset of Malignant Cells Enriched in LM

**FIGURE 2:**
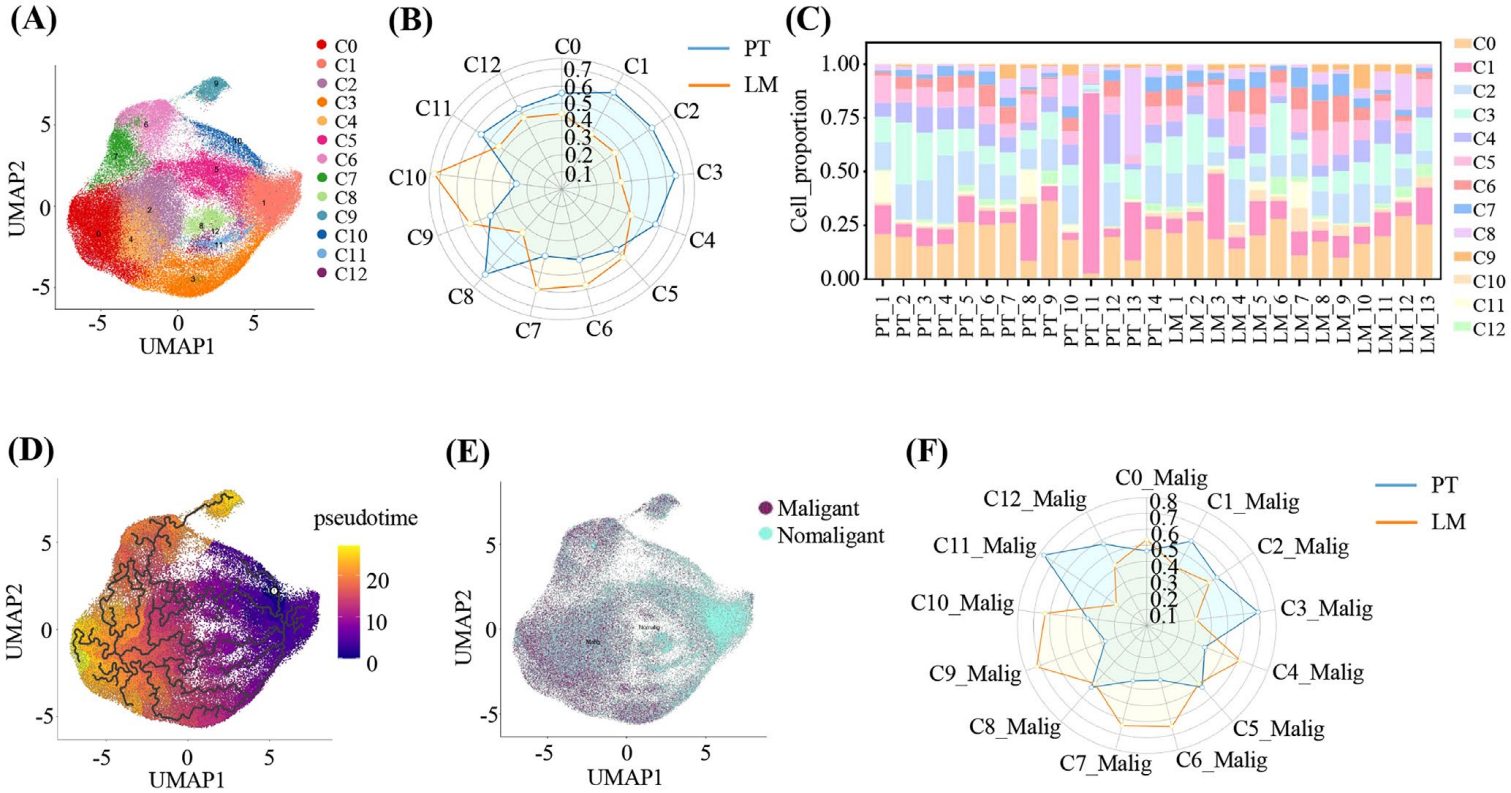
The heterogeneity of epithelial cells. (A) UMAP plot of epithelial cells color‐coded by clusters. (B) Radar plot showing the relative proportions of thirteen epithelial cell clusters in PT and LM. The numbers on the circular ring start from zero and represent the maximum range of cell composition. (C) Stacked plot showing the proportion of different cell clusters in each sample. (D) The differentiation trajectory of epithelial cells inferred by Monocle 3, with cells color‐coded based on pseudotime. (E) UMAP plot showing the malignant cells and nonmalignant cells identified by CopyKAT analysis. (F) Radar plot showing the relative proportions of malignant clusters in PT and LM. Starting with zero, the numbers indicate the maximum range of cell composition. Malig, malignant.

Epithelial cells constitute the predominant cell population and the cellular origin of pancreatic cancer [29]. To explore the heterogeneity of epithelial cells, we partitioned 102,540 epithelial cells into 13 clusters, with 57,783 cells from PT and 44,757 cells from LM (Figure 2A). Each cluster contained cells from both PT and LM, but their proportions varied. Cluster 8 (C8) was more abundant in PT, while C10 was predominantly from LM (Figure 2B). The abundance of different clusters varied substantially among individual samples, indicating significant inter‐patient heterogeneity (Figure 2C). Interestingly, we observed that the proportion of the C1 in patient PT_11 was significantly higher than that of other clusters (Figure 2C). Through gene ontology (GO) analysis, we found that immune response‐activating signaling pathways were significantly enriched in C1, suggesting that C1 had a lower degree of malignancy and was more likely to reside in PT (Figure S2). Moreover, we performed gene set enrichment analysis (GSEA) for biological process pathways in different clusters of epithelial cells. The results showed that pathways related to epidermal development were highly activated in C0. In C1 and C8, immune response activating‐related pathways were highly activated. C2 and C4 showed high activation of pathways related to epithelial and endothelial migration. In C3, C11, and C12, pathways associated with aerobic respiration and metabolic reprogramming were highly activated. C6 and C7 exhibited high activation of pathways related to cell division and proliferation. In C5, pathways related to type II interferon secretion were highly activated. C9 showed high activation of immunosuppressive pathways. In C10, pathways related to protein synthesis were highly activated (Figure S3). The highly activated signaling pathways vary across different clusters, highlighting the significant heterogeneity among epithelial cells. Then we conducted differentiation trajectory analysis by Monocle 3. The differentiation trajectory progressed from clusters C1, C10, and C5 to clusters C0, C7, C6, and C9, with the latter clusters exhibiting a higher degree of malignancy (Figure 2D).

CopyKAT was used to calculate genome‐wide copy number profiles and isolate malignant cells from each epithelial cluster (Figure 2E, Table S1). We further investigated the proportional distribution of malignant cell clusters between PT and LM, finding that C6‐malig, C7‐malig, and C9‐malig were significantly enriched in LM, especially over 70% of C9‐malig originating from LM (Figure 2F).

**FIGURE 3:**
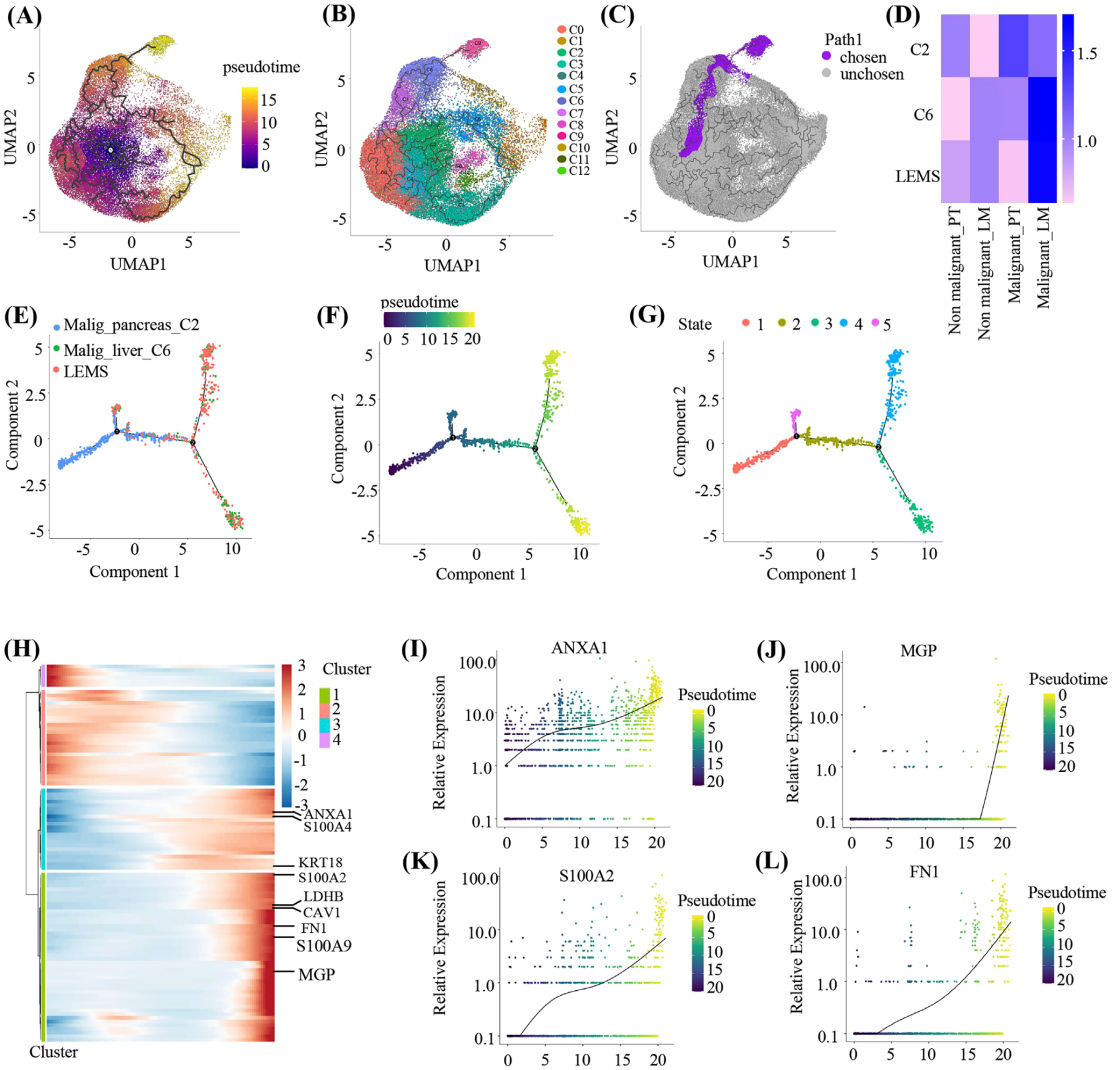
Trajectory analysis of the origin of LEMS specifically enriched in LM. (A, B) The differentiation trajectory of malignant cells inferred by Monocle 3, with cells color‐coded based on pseudotime (A) and clusters (B). (C) The differentiation path of LEMS identified by Monocle 3. (D) The preference of cell clusters in the LEMS differentiation path across different groups estimated by Ro/e score. (E, F) The differentiation trajectory from C2 into C6 and LEMS predicted by Monocle, with cells color‐coded according to clusters (E) and pseudotime (F). (G) The differentiation trajectory of LEMS yielded 5 cellular states. (H) Heatmap of the top 100 differentially expressed genes in pseudotime analysis, categorized into four clusters. (I–L) The expression profiles of key genes across pseudotime.

Pseudotime analysis on all malignant epithelial cells identified C9‐malig as the terminal differentiation branch, indicating an extremely high degree of malignancy. (Figure 3A). Since C9 was primarily enriched in LM, we named it the liver‐enriched metastatic subset (LEMS). To investigate the origin of LEMS, we utilized the choose_graph_segments function and identified a differentiation path starting with C2, progressing through C6, and culminating in LEMS (Figure 3B,C). ROE analysis showed that C6 and LEMS were predominantly composed of liver malignant cells, while C2 was primarily pancreatic malignant cells (Figure 3D). Based on this, we hypothesized that C2 cells from PT migrated, colonized, and differentiated into C6 and LEMS in LM. Pseudotime analysis revealed potential developmental trajectories, with C2 transforming into C6 and LEMS (Figure 3E,F). This trajectory was divided into five cellular states, with C2 being enriched in State 1, LEMS in State 3, and C6 in State 4 (Figure 3G). Key differentiation‐related genes were identified and the top 100 genes in heatmaps were presented, most of which have been proven to be closely related to cancer progression (Figure 3H). Among them, Annexin A1 (ANXA1), Matrix Gla protein (MGP), Fibronectin‐1 (FN1) and S100 calcium binding protein A2 (S100A2) were the top up‐regulated genes in cell differentiation (Figure 3I). Prior studies have demonstrated that ANXA1 and MGP are up‐regulated in various cancers and facilitate cancer immune evasion [30, 31]. FN1 can drive the occurrence and progression of various cancers through metabolic reprogramming [32], while the overexpression of S100A2 can enhance glycolysis and promote cancer cell proliferation [33]. Additionally, we observed a set of downregulated genes in cell differentiation (Figure 3H, Table S3). We performed functional enrichment analysis on these downregulated genes and found that these downregulated genes were associated with pathways related to proliferation and development (Figure S4). The results indicated that the downregulation of these genes might be correlated with a reduced proliferative capacity of tumor cells.

The above studies have confirmed that a multitude of genes upregulated during the LEMS differentiation process had the potential not only to expedite the proliferation of cancer cells but also to initiate immune and metabolic reprogramming.

### 2.3 LEMS Exhibits Metabolic Reprogramming and Immunosuppressive Phenotype

**FIGURE 4:**
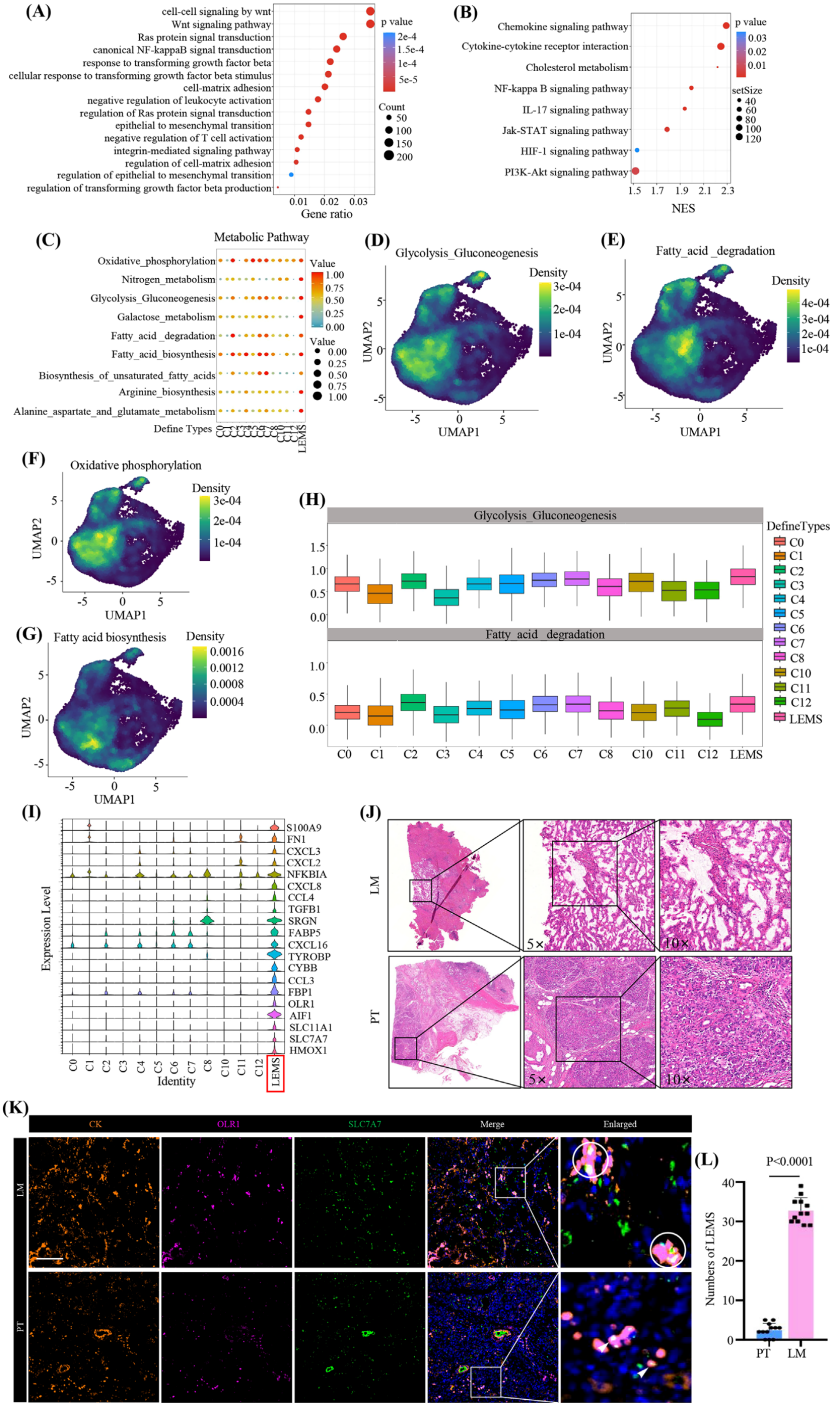
Properties of oncogenicity, immunosuppression and metabolic reprogramming in LEMS. (A) Gene ontology (GO) enrichment of LEMS by using differential expressed genes (DEG). (B) Gene set enrichment analysis (GSEA) analysis revealing the enrichment of metabolism and its related downstream signaling pathways in LEMS. (C) Bubble plot illustrating the highest metabolic score of LEMS compared to other malignant cell clusters by metabolic activity analysis. Both the size and the color of the circle reflect the scaled metabolic score. (D) UMAP plot showing the density of Glycolysis‐Gluconeogenesis pathway scores of all epithelial cells. (E) UMAP plot showing the density of Fatty‐acid‐degradation pathway scores of all epithelial cells. (F) UMAP plot showing the density of Oxidative phosphorylation pathway scores of all epithelial cells. (G) UMAP plot showing the density of Fatty‐acid biosynthesis pathway scores of all epithelial cells. (H) Box plot indicating the increase of metabolic score of the Glycolysis‐Gluconeogenesis and Fatty‐acid‐degradation pathways during the differentiation from C2 to LEMS. (I) Heatmap illustrating the signature DEGs between LEMS and other malignant cell clusters. (J) HE staining of serial sections of the PT and LM clinical samples. (K) Representative multiplexed IF staining images showing the co‐localization of OLR1, SLC7A7 and CK in PT and LM. Images were acquired at 200× magnification. OLR1, pink; SLC7A7, green; CK19, orange; DAPI, blue (*n* = 4 patients, 3 fields assessed per sample). Scale bar, 15 μm. (L) Number of OLR1^+^ SLC7A7^+^ LEMS in PT and LM (*n* = 12).

LEMS was specifically enriched in LM, with numerous oncogenesis and metabolic reprogramming genes upregulated during differentiation [34, 35, 36]. The phenotypic and functional characteristics it possessed and the reason why it tended to be enriched in LM, require further exploration. GO enrichment analysis revealed that EMT, WNT, and RAS‐related pathways were activated in LEMS (Figure 4A). Research indicates that recurrent mutant genes in pancreatic cancer are predominantly enriched in signaling pathways such as KRAS, TGF‐β, WNT, and NOTCH, which is consistent with our findings [37]. KRAS orchestrates intracellular signaling pathways, driving cell proliferation, migration, and survival [38]. WNT signaling interacts with FGF, Notch, Hedgehog, and TGF‐β/BMP pathways [39], suggesting the cancer stem cell‐like characteristics of LEMS. LEMS also exhibited enrichment in pathways related to T cell suppression and TGF‐β production, suggesting its immunosuppressive phenotype (Figure 4A).

GSEA showed activation of the HIF‐1α [40, 41], MAPK [42], and NF‐κB [43] signaling pathways in LEMS (Figure 4B). These pathways are associated with metabolic reprogramming, which can be observed between the primary tumor and metastases [44]. Metabolic reprogramming is the hallmark of malignant tumor cells. Tumor cells can enhance aerobic glycolysis, lipid metabolism, and other metabolic pathways, which can remodel the TME by recruiting many immunosuppressive cells such as TAMs and Tregs and promoting the secretion of regulatory cytokines [45]. Moreover, lactate, a product of aerobic glycolysis, can inhibit the glucose uptake of CD8^+^ T cells, thereby suppressing their tumor‐killing effects [46]. The increased fatty acid uptake by tumor cells can recruit more CCR6^+^ Tregs [47]. Consequently, we performed a targeted enrichment analysis focusing on metabolic pathways and found that, in contrast to other epithelial clusters, a multitude of metabolism‐associated pathways were upregulated in LEMS (Figure 4C), including glycolysis‐gluconeogenesis, fatty acid degradation, oxidative phosphorylation, and fatty acid biosynthesis signaling pathways (Figure 4D–G). Interestingly, we found that during the differentiation of C2 into LEMS, only the glycolysis‐gluconeogenesis signaling pathway gradually intensified (Figure 4H). This suggests that LEMS, as a terminally differentiated malignant tumor cell subpopulation, exhibits a high glycolytic phenotype. Moreover, we observed that the pathway‐enrichment scores for both fatty‐acid synthesis and degradation declined during the early C2‐to‐LEMS transition but rebounded at the terminal stage (Table S4). We speculated that during early tumorigenesis, the activation of oncogenes promoted fatty‐acid synthesis and degradation. In the middle phase, lipid metabolism was suppressed by the accumulation of lactate and ROS. In the late phase, metastatic dissemination and adaptive reprogramming re‐activated lipogenesis and fatty‐acid oxidation.

Metabolic and immunosuppressive marker genes, including OLR1, SLC7A7, FBP1, FABP5, NFKBIA, and TGF‐B1, were enriched in LEMS (Figure 4I). OLR1, as an oxidized low‐density lipoprotein receptor, exhibits a positive correlation with the infiltration of immunosuppressive cells, while negatively correlating with effector T cells [48]. It has been validated that OLR1 can promote metastases by upregulating the expression of c‐MYC to enhance the transcription of HMGA2 [49]. OLR1 can promote de novo lipogenesis and activate the NF‐kB signaling pathway to inhibit tumor cell apoptosis and promote cancer progression [50]. In addition, solute carrier family 7 member 7 (SLC7A7), a cationic amino acid transporter, is associated with a poor prognosis [51]. And SLC7A7 is enriched during the EMT process and may facilitate colorectal cancer metastases through the SLC7A7/APC/WNT/β‐catenin signaling pathway [52]. It has been reported that SLC7A7 is a key factor in regulating lipid metabolism, and the ATF3‐SLC7A7‐mTORC1 signaling axis plays an important role in lipogenesis and tumorigenesis [53]. These indicate the potential role of SLC7A7 and OLR1 in metabolic reprogramming. However, there are very few reports on the roles of OLR1 and SLC7A7 in pancreatic cancer liver metastases.

We utilized serial sections of the PT and LM clinical samples and performed HE staining and multiplex immunofluorescence (mIF) experiments simultaneously. We verified the lesions in PT and LM through HE staining (Figure 4J). The results of mIF experiments indicated that CK19 expression was associated with tumor cells in PT and LM, and the co‐localization of OLR1 and SLC7A7 was significantly higher in LM than in PT, highlighting the potential of OLR1 and SLC7A7 as diagnostic biomarkers for patients with pancreatic cancer liver metastases (Figure 4K,L).

Furthermore, we analyzed the expression levels of OLR1 and SLC7A7 in pancreatic cancer patients using the GPEIA2. The results showed that OLR1 and SLC7A7 were highly expressed in pancreatic cancer patients and associated with the disease stage, indicating that OLR1 and SLC7A7 were important for the diagnosis of pancreatic cancer (Figure S5).

### 2.4 The Immune Microenvironment and Distinct Subtypes of Macrophages in PT and LM

**FIGURE 5:**
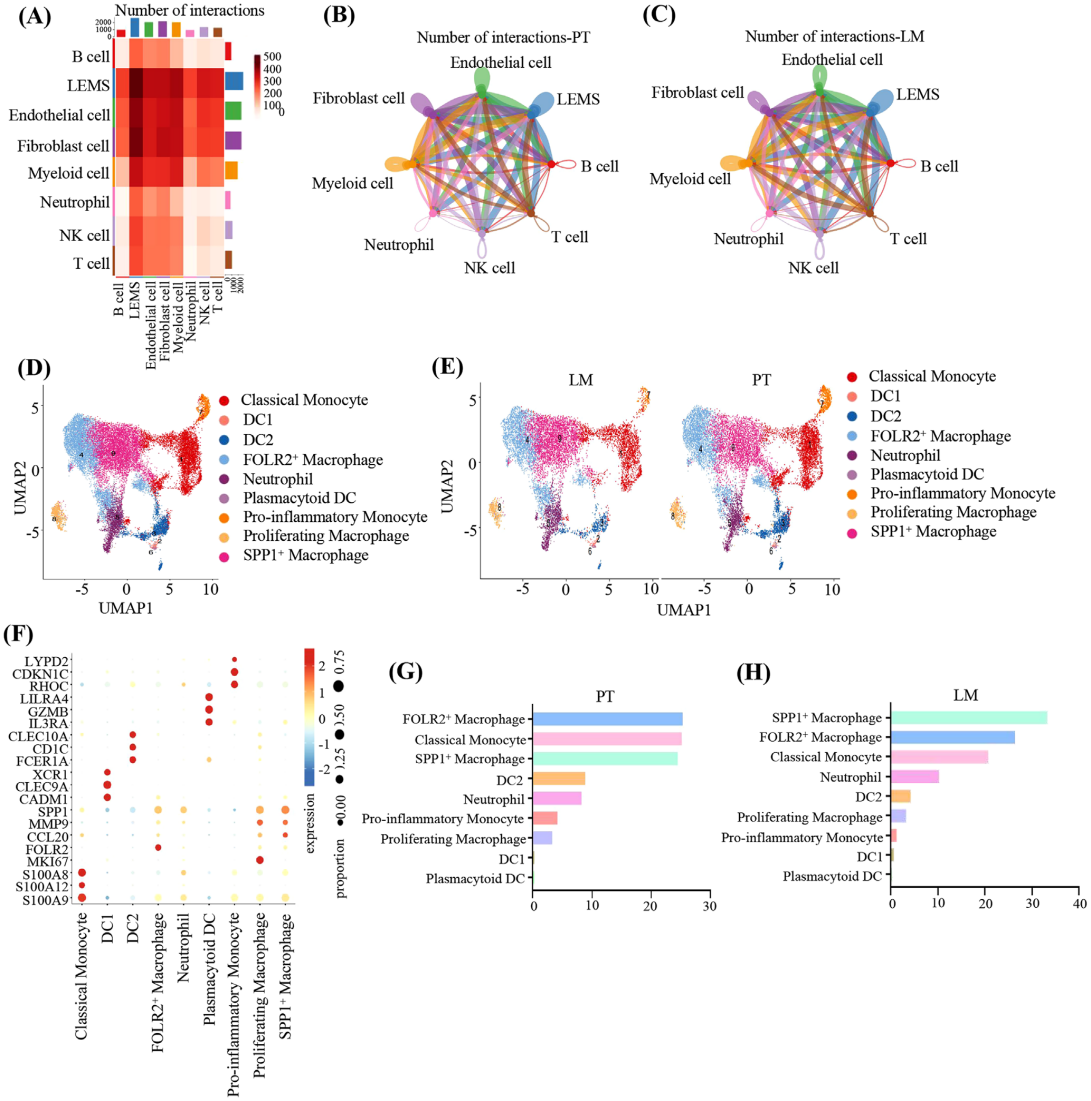
The diversity of the immune microenvironment in the PT and LM of pancreatic cancer. (A) The heatmap showing the communication probability between LEMS and other cell types. (B, C) Network diagram showing the predicted interaction frequency of ligand‐receptor between LEMS and other cell types in PT (B) and LM (C). (D) UMAP plot of myeloid cells color‐coded by cell subtypes. (E) UMAP plot displaying the distribution of myeloid cell subtypes, categorized by PT and LM. (F) Bubble plot showing representative marker genes in nine myeloid cell subtypes. The size represents the proportion of cells expressing the genes and the color represents mean expression levels of the genes. (G, H) The abundance and proportions of myeloid cell subtypes in PT (G) and LM (H), ranked in descending order.

In the TME, diverse components can either promote fibrosis and immune suppression at both primary and metastatic sites or facilitate tumor metastases through mechanisms such as angiogenesis, lymphangiogenesis, EMT, cancer cell invasion and migration, and the establishment of pre‐metastatic niches [54, 55]. Given the multifaceted roles of these components, we utilized Cell‐chat to dissect the interactions between LEMS and other cell subtypes. LEMS were observed to engage in interactions with a wide array of cell types, with a particularly high intensity on myeloid cells (Figure 5A–C).

To further explore the potential role of specific myeloid subpopulations in LEMS invasion and metastases, we performed sub‐clustering analysis of myeloid cells, identifying nine distinct clusters: classical monocytes, folate receptor 2 (FOLR2^+^) macrophages, pro‐inflammatory monocytes, proliferating macrophages, SPP1^+^ macrophages, DC1, DC2, neutrophils, and plasmacytoid DC (Figure 5D–F). Among these clusters, FOLR2^+^ macrophages were the most abundant in PT, whereas SPP1^+^ macrophages predominated in LM (Figure 5G,H).

**FIGURE 6:**
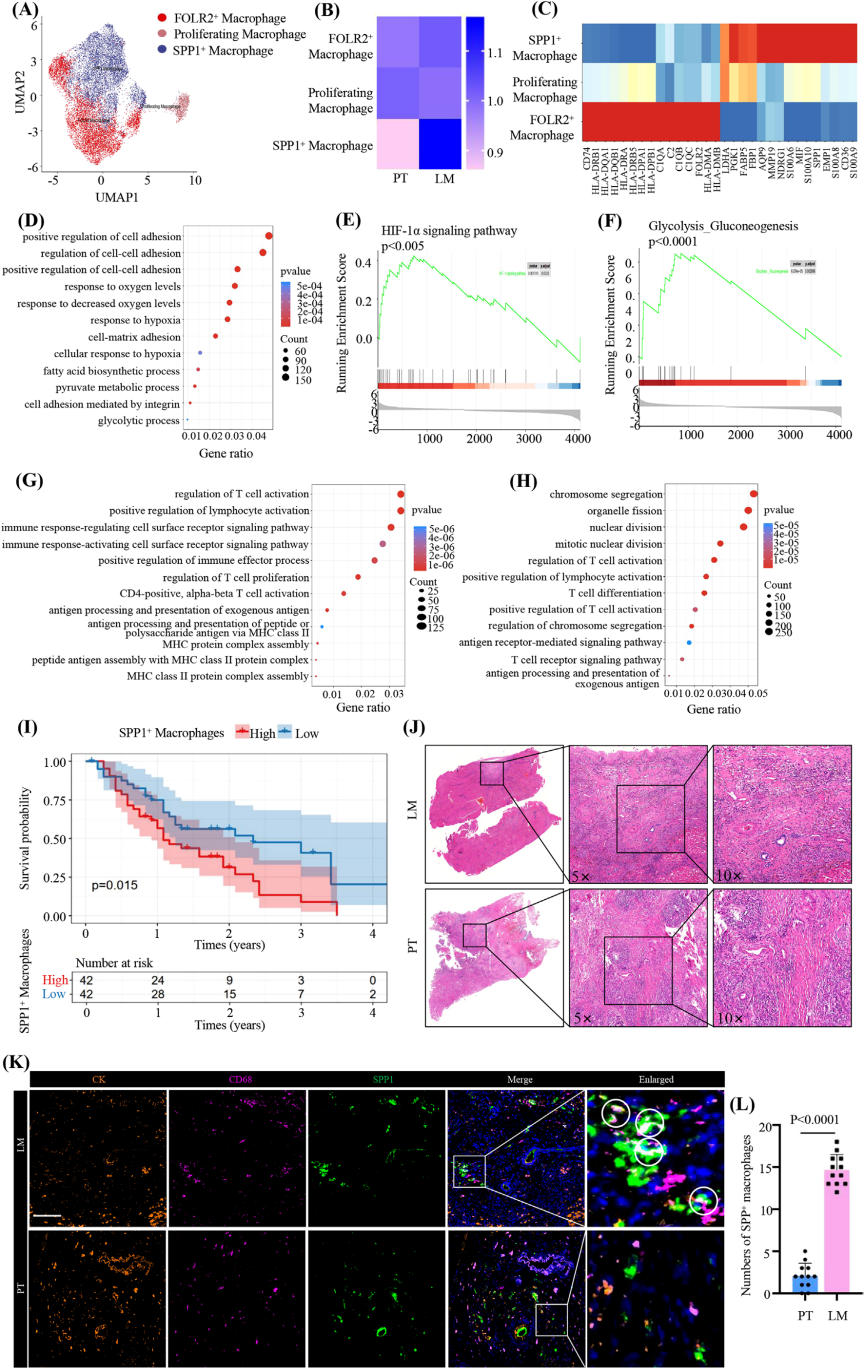
SPP1^+^ macrophages with properties of metastases‐promoting and metabolic reprogramming enriched in LM. (A) UMAP plot showing the re‐clustering of three macrophage subpopulations. (B) The preference of three macrophage subpopulations across different groups estimated by Ro/e score. (C) Heatmap illustrating the signature DEGs among three macrophage subpopulations. (D) GO analysis highlighting the enrichment of pathways associated with metastases promotion and metabolic reprogramming in SPP1^+^ macrophages. (E, F) GSEA results showing HIF‐1α signaling pathway (E) and Glycolysis‐Gluconeogenesis signaling pathway (F) upregulated in SPP1^+^ macrophage. (G) GO analysis indicating the enrichment of pathways associated with antigen presentation and immune response in FOLR2^+^ macrophage. (H) GO analysis indicating the enrichment of pathways associated with cell division and proliferation in proliferating macrophage. (I) Kaplan–Meier survival curves showing the influence of SPP1^+^ macrophages on the survival of pancreatic cancer patients. (J) HE staining of serial sections of the PT and LM clinical samples. (K) Representative multiplexed IF staining showing the spatial characterization of SPP1^+^ macrophages in PT and LM. Images were acquired at 200× magnification. CD68, pink; SPP1, green; CK19, orange; DAPI, blue (*n* = 4 patients, 3 fields assessed per sample). Scale bar, 15 μm. (L) Bar plot quantifying the number of SPP1^+^ macrophages in PT versus LM samples (*n* = 12).

To investigate the role of macrophages in the liver metastases of pancreatic cancer, we conducted a separate clustering analysis of macrophages (Figure 6A). Ro/e analysis revealed that SPP1^+^ macrophages were significantly enriched in LM compared to PT (Figure 6B). Previous studies have highlighted the pivotal roles of CXCL9 and SPP1 in determining TAM polarization [56, 57]. Furthermore, SPP1^+^ macrophages were shown to accumulate preferentially in hypoxic and necrotic tumor regions, correlating with poor outcomes in colon cancer patients [58]. These findings underscore how SPP1^+^ macrophages are involved in tumor progression and metastases.

In contrast to FOLR2^+^ macrophages and proliferating macrophages, SPP1^+^ macrophages exhibited a distinct set of metabolic genes, including FABP5, LDHA, PGK1, and CD36 (Figure 6C). GO analysis indicated that SPP1^+^ macrophages were involved in pathways related to cell migration, hypoxia, and metabolic reprogramming (Figure 6D). Hypoxia, a hallmark feature of the tumor microenvironment in solid malignancies, triggers a marked upregulation of hypoxia‐inducible factors (HIFs) in cells under low oxygen conditions [59]. GSEA identified significant activation of the HIF‐1α signaling pathway and glycolysis/gluconeogenesis pathways in SPP1^+^ macrophages, suggesting their potential to drive metastases and metabolic reprogramming (Figure 6E,F). It has been reported that the metabolic profile of TAM and the metabolites in TME have great influence in regulating the function and polarization of TAMs [60]. Macrophages can polarize into M1 antitumor and M2 pro‐tumor basic types [61]. Therefore, we further evaluated the expression of M1 and M2 related signature genes in SPP1^+^ macrophages. The results showed that the scores for both M1 and M2 macrophage related marker genes were elevated in SPP1^+^ macrophages in LM compared to PT. Moreover, the score for M2‐related genes was higher than M1‐related marker genes in SPP1^+^ macrophages, indicating that SPP1^+^ macrophages tend to exhibit a pro‐metastatic phenotype (Figure S6). FOLR2^+^ macrophages, in contrast, were enriched in immune response and antigen processing pathways (Figure 6G), indicating their role in immune activation. In proliferating macrophages, pathways related to cell division and proliferation were strongly activated (Figure 6H). Moving forward, we utilized Bulk‐seq data from pancreatic cancer patients in the public database GSE28735 and conducted joint deconvolution analysis with single‐cell sequencing data. The result showed that pancreatic cancer patients with a high proportion of SPP1^+^ macrophages had a significantly shorter survival, indicating the important role of SPP1^+^ macrophages in pancreatic cancer progression and patient prognosis (Figure 6I). To validate the significance of SPP1^+^ macrophages in the metastatic process, we utilized serial sections of the PT and LM clinical samples and performed HE staining and mIF experiments simultaneously. We identified the lesions in PT and LM through HE staining (Figure 6J). The results of mIF showed that in both LM and PT lesions, SPP1^+^ macrophages were likely close to CK19‐positive tumor cells spatially (Figure 6K). Moreover, compared to PT lesions, the enrichment of SPP1^+^ macrophages was higher in LM lesions (Figure 6L). These findings collectively highlighted the distinct functional roles and metabolic adaptations of SPP1^+^ macrophages in the context of tumor progression and metastases.

Moreover, we analyzed the interactions between LEMS and NK cells and found that there were some differential receptor‐ligand pairs between PT and LM, such as MIF‐(CD74 + CXCR4), HLA‐E‐KLRC2, and HLA‐E‐CD94:NKG2C, which were highly expressed in LM (Figure S7A). And we also analyzed the interactions between LEMS and T cells and found that receptor‐ligand pairs such as SPP1‐CD44, MIF‐(CD74 + CXCR4), and MIF‐(CD74 + CD44) were highly expressed in LM (Figure S7B). It has been reported that early metastatic epithelial cells communicate with other immune cells through receptor‐ligand pairs such as MIF‐(CD74 + CXCR4) and MIF‐(CD74 + CD44) [62]. Therefore, we speculated that these specific receptor‐ligand pairs also played an important role in pancreatic cancer liver metastases.

### 2.5 Crosstalk Between LEMS and SPP1 ^+^ Macrophages Defines a Pro‐Metastatic Niche

**FIGURE 7:**
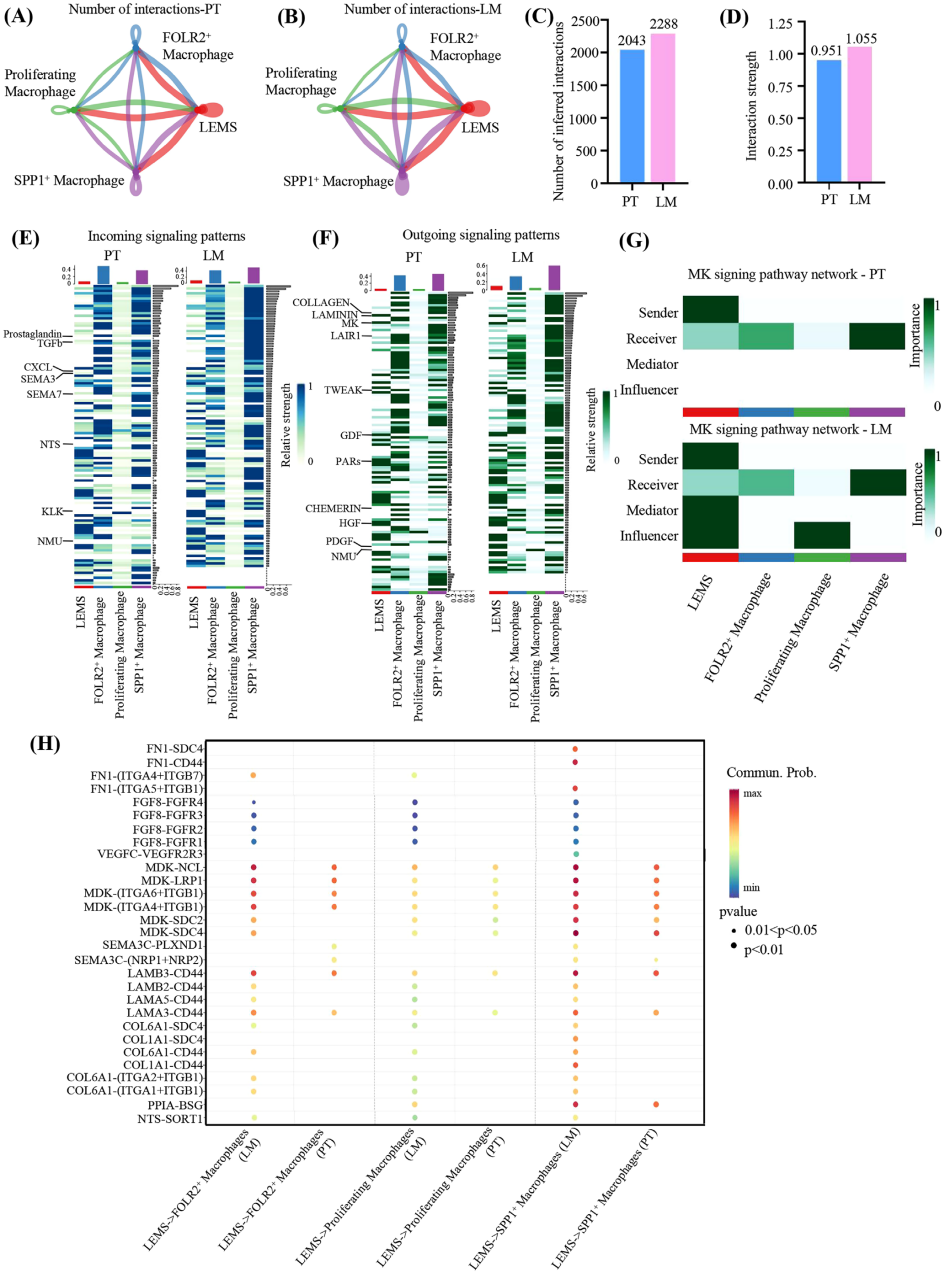
The different communication of LEMS and macrophages between PT and LM. (A, B) Network diagram showing the predicted interaction frequency of ligand‐receptor between LEMS and three macrophage subpopulations in PT (A) and LM (B). (C, D) The bar chart illustrating the interaction number (C) and strength (D) in PT and LM. (E, F) The incoming (E) and outgoing (F) signaling patterns of LEMS and macrophages between PT and LM. (G) The heatmap highlighting the key senders, receivers, mediators, and influencers in the MK signaling pathways between PT and LM, as inferred by network centrality analysis. (H) The important differential receptor‐ligand pairs from LEMS to macrophages in PT and LM. The color of the dot reflects the communication probability, with blank indicating a communication probability of zero. The size of the dot corresponds to the *p*‐value.

To elucidate the crosstalk between LEMS and macrophages, we compared cellular signaling flux patterns among LEMS, SPP1^+^ macrophages, FOLR2^+^ macrophages, and proliferating macrophages in PT and LM. Compared with PT, the frequency and intensity of interactions between LEMS and macrophages were higher in LM (Figure 7A–D). We observed distinct alterations in signaling dynamics. Outgoing signaling from LEMS, including GDF, CHEMERIN, PDGF, and NMU, was significantly enhanced in LM, while incoming signaling to LEMS, such as SEMA, NMU, NTS, and KLK, was predominantly enriched in LM (Figure 7E,F). These signaling shifts are likely pivotal in driving LEMS liver metastases. The growth differentiation factor (GDF) signaling pathway is known to promote tumor cell invasion and immune modulation via enhancing cytotoxic T cell infiltration and interferon γ expression, emerging as a therapeutic target in clinical trials [63]. Similarly, Kallikrein‐related peptidases (KLK)–mediated proteolytic cascades play a critical role in tumor progression by remodeling the TME [64].

In LM, the incoming signaling patterns of Prostaglandin, TGF‐β, and CXCL in SPP1^+^ macrophages were significantly enhanced, while the outgoing signal patterns of TWEAK, LAIR1, and PAR were markedly increased compared to PT (Figure 7E,F). These changes may drive metabolic reprogramming in SPP1^+^ macrophages, fostering a pro‐metastatic niche. For instance, TGF‐β upregulates the glycolytic enzyme PFKL, stimulating glycolysis and suppressing pro‐inflammatory cytokine production [65]. TWEAK, via its receptor FN14, promotes EMT and tumor cell migration [66].

Further analysis revealed that Midkine (MK), LAMININ, GDF and HGF pathways constituted the main outgoing signaling flows from LEMS in LM (Figure 7F). In the MK signaling network, LEMS transitioned from a sender in PT to the dominant sender, mediator, and influencer in LM, correlating with enhanced malignant cell invasion and migration (Figure 7G). Ligand‐receptor communication analysis identified specific interactions between LEMS and macrophages in LM, which might be implicated in immune suppression and metastases (Figure 7H, Table S2). Among these specific interactions, several receptor‐ligand pairs such as MDK‐SDC4, COL6A1‐CD44, and FN1‐CD44 have been reported to contribute to the formation of an immunosuppressive microenvironment and promote tumor metastases. COL6A1 is highly expressed in the malignant tumor cell subpopulation and has the potential to participate in extracellular matrix (ECM) and local lesion adhesion [62]. CD44, a stemness marker, serves as a crucial component in the signaling pathway responsible for inducing macrophage migration [67]. The interaction between MDK‐high tumor cells and SPP1^+^ macrophages has been identified as the key mechanism contributing to the immunosuppressive TME [68]. FN1‐CD44 receptor‐ligand pair can promote tumor metastases and invasion [69]. Therefore, we inferred that the interaction between LEMS and SPP1^+^ macrophages contributed to the formation of a pro‐metastatic microenvironment. However, through cell‐chat analysis between SPP1^+^ macrophages and 13 distinct clusters, we found that similar interactions were also presented in other malignant cells. But there were several specific receptor‐ligand pairs such as PGE2‐PTGES3‐PTGER4 and APOE‐TREM2 between LEMS and SPP1^+^ macrophages (Figure S8). Moving forward, we planned to investigate the mechanisms by which these specific receptor‐ligand pairs promote metastases.

Collectively, these findings suggest that enhanced ligand‐receptor interactions between LEMS and SPP1^+^ macrophages in LM foster an immunosuppressive and pro‐metastatic niche, though further mechanistic studies are warranted.

## 3 Discussion

In this study, we integrated two independent single‐cell sequencing datasets and identified a terminally differentiated malignant cell subset, termed LEMS, which was specifically enriched in LM of pancreatic cancer. LEMS exhibited a unique phenotype characterized by metabolic reprogramming and immune suppression, driven by the activation of key oncogenic pathways such as WNT, RAS, and TGF‐β. The marker genes OLR1 and SLC7A7, which were significantly upregulated in LEMS, were validated in clinical samples, suggesting their potential as biomarkers for pancreatic cancer liver metastases. Furthermore, we identified SPP1^+^ macrophages as a critical component of the TME in LM, where they interacted with LEMS to enhance tumor cell migration and invasion.

Exploring the underlying mechanisms and identifying potential biomarkers and therapeutic targets have great clinical importance for patients with pancreatic cancer liver metastases. The composition of pancreatic cancer cells with high inter‐patient heterogeneity is a crucial determinant in classifying PDAC subtypes [70]. According to distinct immune and metabolic profiles, malignant cells are classified into five subtypes and the upregulated genes REG4 and SPINK1 represent potential therapeutic targets [71]. A unique malignant cell cluster, Ep_VGLL1, with transitional characteristics bridging the basal‐like and classical subtypes is identified and associated with poor prognosis [72]. However, research on the differences between malignant cells in PT and LM remains limited, and the origins of metastatic malignant cells and the mechanisms of metastases are still underexplored.

The identification of LEMS as a distinct subset of malignant cells enriched in liver metastases underscores the importance of cellular heterogeneity in pancreatic cancer progression. LEMS exhibits a highly malignant phenotype, with activation of cancer stem cell‐related pathways such as WNT and RAS, which are known to drive tumor cell proliferation, migration, and survival [38, 39]. The enrichment of immune‐suppressive pathways, including TGF‐β signaling, in LEMS further highlights its role in creating an immunosuppressive TME that facilitates immune evasion and metastatic colonization. This is in agreement with prior investigations revealing that pancreatic cancer cells can modulate the TME to promote immune suppression and metastases [73]. The metabolic reprogramming observed in LEMS, particularly the upregulation of glycolysis and fatty‐acid degradation pathways, aligns with the known metabolic adaptations of cancer cells during metastases. The shift toward a glycolytic phenotype in LEMS likely sustains the elevated bioenergetic needs of rapidly proliferating and migrating tumor cells, while also contributing to the acidic and hypoxic conditions that further suppress immune responses [44, 74]. These results reveal that LEMS is a highly malignant subpopulation distinguished by a pronounced tropism for metastatic dissemination, coupled with synergistic activation of immune and metabolic pathways. The identification of key metabolic genes such as OLR1 and SLC7A7 in LEMS provides potential targets for disrupting these metabolic adaptations and impairing metastatic progression. Although it has been reported that OLR1 is expressed in hepatic stellate cells and is necessary for their fibrotic activation, its expression in other pancreatic cells has not been reported. By using OLR1 and SLC7A7 as markers for LEMS, we can effectively exclude this influence. However, this analysis involves a relatively small clinical cohort. As an exploratory study, it inherently shares the limitations common to single‐cell research. In future studies, we will expand the clinical cohort to validate these findings. The specific mechanisms by which LEMS promotes liver metastases remain to be elucidated, and validation of its signature genes OLR1 and SLC7A7 requires expanded clinical sampling. Moreover, the expression of key genes in these activated signaling pathways is needed to further validate through qPCR and Western blotting experiments. We will construct pancreatic cancer liver metastases models in mice using SLC7A7 and OLR1 knockout pancreatic cancer cells to verify their crucial roles in pancreatic cancer liver metastases. Further studies are needed to investigate the correlation of OLR1 and SLC7A7 with pathological staging and survival in pancreatic cancer patients through immunohistochemistry. Concurrently, we aim to design small molecule drugs targeting OLR1 and SLC7A7 to explore their therapeutic effects.

Our findings also highlight the critical role of the crosstalk between LEMS and SPP1^+^ macrophages in the liver metastatic niche. These macrophages are highly enriched in LM and exhibit a pro‐metastatic phenotype, characterized by the activation of pathways related to cell migration, hypoxia, and metabolic reprogramming. It has been reported that SPP1^+^ macrophages are significantly associated with epidermal growth factor receptor (EGFR) amplification, compromised T cell function and poor patient outcomes [75]. The interaction between SPP1 ligand and PD‐L1 expressed on SPP1^+^ macrophages promotes CD8^+^ T cell exhaustion [76]. In liver metastases, TGF‐β and IL‐6 can deliver signaling to the liver and promote the enrichment of intermediate clusters associated with fibronectin and SPP1 expression, and induce tumor‐specific T cell exhaustion [77]. In our study, the interaction between LEMS and SPP1^+^ macrophages, mediated by specific receptor‐ligand pairs such as MDK‐SDC4, FN1‐CD44, and COL6A1‐CD44, suggests a cooperative relationship that enhances tumor cell migration and invasion. This result aligns with previous studies demonstrating that SPP1^+^ macrophages foster the establishment of a pre‐metastatic niche and promote immune suppression in the TME [78, 79]. Certainly, other differential receptor‐ligand pairs also hold great research value. The reason why SPP1^+^ macrophages preferentially colonize liver metastases is unknown, and whether LEMS and SPP1^+^ macrophages specifically promote metastases through the aforementioned receptor‐ligand pairs needs further validation. However, due to the significant challenges in collecting clinical samples from PT and LM, it is extremely difficult for us to isolate a sufficient number of LEMS, SPP1^+^ macrophages and C2 subset to investigate their mechanisms in promoting metastases. Currently, we are expanding the collection of clinical samples from PT and LM, with the aim of isolating enough LEMS, SPP1^+^ macrophages and C2 subset to conduct mechanistic researches. Considering the limitations of single‐cell sequencing in resolving dynamic changes in proteins and metabolites, we plan to integrate multi‐omics sequencing technologies for further analysis. We plan to perform proteomics and metabolomics analysis, integrating single‐cell sequencing data to explore the expression and function of key proteins. And we will use corresponding antibodies to block these interactions and screen for key regulatory molecules in pancreatic cancer liver metastases. Additionally, we plan to isolate SPP1^+^ macrophages, co‐culture them with tumor cells, and investigate the impact and regulatory mechanisms of SPP1^+^ macrophages on tumor cells. Moreover, we will establish a transwell co‐culture model of LEMS and macrophages to investigate the impact of macrophages on the migration and invasion capabilities of LEMS subpopulations, thereby validating the interplay between LEMS and macrophages.

In conclusion, our study identifies LEMS as a key driver of pancreatic cancer liver metastases, characterized by metabolic reprogramming and immune suppression. The interaction between LEMS and SPP1^+^ macrophages creates a pro‐metastatic niche that facilitates tumor cell migration and invasion. These findings provide novel perspectives on the mechanisms of pancreatic cancer liver metastases and identify promising diagnostic markers and therapeutic targets for this aggressive malignancy.

## 4 Methods

### 4.1 Open Source Data Acquisition

The single‐cell sequencing data utilized in this analysis were sourced from publicly accessible databases and featured in high‐impact journals. Specifically, the raw data were retrieved from the GEO database (accession numbers GSE205013 and GSE197177). Following the exclusion of treatment groups, a total of 14 primary pancreatic cancer samples and 13 pancreatic cancer liver metastasis samples were ultimately included in the study.

### 4.2 Data Processing and Cell Type Annotations

Harmony algorithm can project cells into a shared embedding space, where cells cluster based on their cell type rather than dataset‐specific conditions [80]. We used it to analyze data from different platforms or time points, effectively removing batch effects between different datasets while preserving biological variability. Dimensionality reduction was performed by employing the Run PCA and Run UMAP functions within the Seurat package. Subsequently, cell clusters were delineated using the FindClusters function in Seurat, with a default resolution of 0.8. For each subpopulation, marker genes were analyzed using the Wilcoxon algorithm. Candidate marker genes for individual clusters were selected by comparing the gene expression profiles of cells within each cluster to those in the remaining clusters, based on the average log fold change (avg_logFC) in expression levels. To further identify cell subclusters and annotate cell subpopulations, we repeated the normalization, dimensionality reduction, and clustering processes described above. For the initial cell annotation, cells were identified based on the following markers: T cells (CD3D, CD3E, and CD3G), B cells (MS4A1, CD79A, and CD79B), myeloid cells (CD68, CD14, and LYZ), endothelial cells (CLDN5, VWF, and PECAM1), epithelial cells (EPCAM, KRT19, and KRT18), fibroblasts (DCN, COL3A1, and COL1A1), NK cells (NKG7, GNLY, and FGFBP2), neutrophils (FCGR3B, CXCL8, and CSF3R).

### 4.3 Functional Enrichment Analysis

The “Find All Markers” function in Seurat was utilized to identify differentially expressed genes. A gene was deemed differentially expressed if its log_2_ fold change (Log FC) in the proportion of cells expressing the gene was ≥ 0.25 and the adjusted *p*‐value was < 0.05 compared to other cells. GO enrichment analysis was carried out using the R package “Cluster Profiler”, with the results visualized in a bubble chart. GSEA analysis does not necessitate the identification of differential genes. Instead, it operates at the gene set level, leveraging the KEGG database to evaluate biological pathways. A gene set is deemed statistically significant when the absolute value of the normalized enrichment score |NES| > 1 and the *p*‐value < 0.05.

### 4.4 Single Cell Tissue Preference Analysis

The preference of each subpopulation for different groups was quantified using the Ro/e index through statistical methods. By comparing the distribution of cell subpopulations across different groups, we analyzed the preferences of these subpopulations in various groupings. The criteria for classification are as follows: Ro/e > 1 indicates that the cell subpopulation is highly specific to the tissue (+++); 0.8–1 (++); 0.2–0.8 (+); 0–0.2 (+/−); 0 (−).

### 4.5 Identification of Malignant Tumor Cells by Using CopyKAT

To identify malignant and nonmalignant cells, all epithelial cells from the single‐cell dataset were extracted and employed the CopyKAT v1.1.0 software package to differentiate between aneuploid and diploid cells based on copy number variation.

### 4.6 Pseudotime Analysis

In this study, Monocle 2 and Monocle 3 were employed to investigate the potential differentiation trajectories of malignant cells. The Seurat object was imported into Monocle via the “as.cell data set” function. Using the R package Monocle 3, we conducted pseudotime analysis, which revealed that LEMS was a terminally differentiated subpopulation of malignant cells. Then the “choose graph segments” function was used to explore the differentiation pathways of LEMS cells and construct a pseudotime trajectory based on C2, C6, and LEMS with Monocle 2. Following dimensionality reduction and cell ordering, the cells were mapped and organized into distinct branching trajectories. Cells grouped within the same branch were classified in an identical biological state. Ultimately, cells were ordered along the pseudotime trajectory using the “order cells” function, and the dynamic changes in gene expression over pseudotime were visualized using the “plot genes in pseudotime” function.

### 4.7 ScMetabolism Analysis

ScMetabolism tool was employed to analyze the single‐cell metabolism, utilizing both VISION and AU Cell analysis methods. The analysis results were stored in Seurat in the form of metabolic pathway activity scores, which were then used for further comparisons between cell clusters or cellular states. Additionally, specific pathways were visualized using UMAP.

### 4.8 Cell–Cell Communication Analysis

The Cell‐chat software was employed to predict, infer, and characterize the communication between different cells, with the expression matrix normalized using Seurat. A list of ligand‐receptor pairs was obtained from CellChatDB. Differences in ligand‐receptor pairs between different cells or groups were analyzed based on the proportion of cells expressing each gene and the average expression level of genes within cell clusters.

### 4.9 Multiplex Immunofluorescence (mIF) Staining

Formalin‐fixed, paraffin‐embedded (FFPE) specimens of PDAC primary tumors and liver metastases were sourced from the First Affiliated Hospital of Naval Medical University. The paraffin sections were deparaffinized through a graded series of xylene and ethanol and rehydrated. Antigen retrieval was then performed by heating the specimens in citrate buffer (pH 6.0) using a pressure cooker. Once cooled to ambient temperature, the sections were treated with 3% hydrogen peroxide for 10 min to inhibit endogenous peroxidase activity, followed by blocking with goat serum for an additional 10 min. The primary antibodies were added and incubated at ambient temperature for 30 min. Afterward, the sections were rinsed three times with PBS, followed by incubation with secondary antibodies under the same conditions for an additional 30 min. After an additional series of three PBS rinses, the sections were treated with TSA (tyramide signal amplification) reagent at ambient temperature for 10 min and washed three more times with PBS to prepare for subsequent antigen retrieval steps. Once all antigen labeling was complete, nuclei were stained with DAPI. The stained slides were imaged at 20× magnification using a Leica microscope. Multiple primary antibodies were used, including CK19 (GB12198‐100, Servicebio, 1:500), SLC7A7 (CSB‐PA892349LA01HU, CUSABIO, 1:100), OLR1 (57,946, Cell Signaling Technology, 1:200), CD68 (GB113150‐50, Servicebio, 1:100), SPP1 (ab214050, abcom, 1:100).

Criteria for field‐of‐view selection: First, we identified the tumor region as the core reference and established all subsequent evaluation criteria relative to this tumor focus. Then we randomly selected three fields of view for quantitative assessment using confocal *z*‐stacks acquired at a defined axial step size. The definition of the positive signal threshold: Following background subtraction, positive signal areas were automatically identified using ImageJ software with a predefined fluorescence intensity threshold set at > 3 times the mean background intensity.

### 4.10 Statistical Analysis

Group mean comparisons were conducted with Student's two‐tailed t‐test. Statistical analysis was conducted using Prism 8 (version 8.0.2).

## Author Contributions

**Xuan Yang:** investigation, writing – original draft, data curation. **Xinyuan Chen:** investigation, writing – original draft. **Zixin Wang:** investigation. **Yanfang Liu:** data curation, resources. **Huiting Hu:** investigation. **Qingru Wu:** investigation. **Hailing Zhang:** funding acquisition, supervision. **Yu Xiong:** methodology, formal analysis, data curation. **Xin Li:** methodology, formal analysis, data curation. **Xiaotao Cheng:** methodology, formal analysis, data curation. **Xiaoyu Ruan:** supervision, writing – review and editing. **Yan Gu:** supervision, writing – review and editing, funding acquisition.

## Ethics Statement

This study was approved by the Shanghai Changhai Hospital Ethics Committee (CHEC2024‐109) and performed All participants provided written informed consent.

## Conflicts of Interest

The authors declare no conflicts of interest.

## Supporting information


**Figure S1:** Data processing. (A, B) The UMAP plots respectively illustrating the integration of different GEO datasets before (A) and after (B) batch effects correction through the Harmony algorithm.
**Figure S2:** The enrichment of immune response‐activating signaling pathways in C1. Bubble plot showing the activation of immune response‐activating signaling pathways in C1 analyzed by GO enrichment analysis.
**Figure S3:** The biological function of 13 distinct epithelial clusters. Bubble plot illustrating the different pathways enriched in each epithelial cluster.
**Figure S4:** GO analysis of downregulated genes in the differentiation trajectory of malignant cells. The downregulated genes in the differentiation trajectory of malignant cells were associated with pathways related to cell proliferation and development analyzed by GO analysis.
**Figure S5:** The expression of OLR1 and SLC7A7 in pancreatic cancer patients. (A)The expression of OLR1 in pancreatic cancer tissues and normal tissues. (B) The expression of OLR1 at different stages of pancreatic cancer. (C) The expression of SLC7A7 in pancreatic cancer tissues and normal tissues. (D) The expression of SLC7A7 at different stages of pancreatic cancer.
**Figure S6:** The expression of M1 and M2 related signature genes in SPP1^+^ macrophages. Ridgeplot showing the expression of M1 and M2 related signature genes in SPP1^+^ macrophages between PT and LM.
**Figure S7:** Cell‐chat of LEMS and immune cells between PT and LM. (A)The important differential receptor‐ligand pairs from LEMS to NK cells in PT and LM. (B) The important differential receptor‐ligand pairs from LEMS to T cells in PT and LM. The color of the dot reflects the communication probability, with blank indicating a communication probability of zero. And the size of the dot corresponds to the *p*‐value.
**Figure S8:** Cell‐chat of SPP1^+^ macrophages and 13 epithelial clusters between PT and LM. The important differential receptor‐ligand pairs from SPP1^+^ macrophages to distinct epithelial clusters in PT and LM. The color of the dot reflects the communication probability, with blank indicating a communication probability of zero. And the size of the dot corresponds to the *p*‐value.

**Table S1:** The raw processed data of CopyKAT results for malignant and non‐malignant epithelial cells.

**Table S2:** The different receptor‐ligand pairs between LEMS and macrophages.

**Table S3:** Top 100 differentially expressed genes identified by pseudotemporal trajectory analysis.

**Table S4:** The score of key metabolic pathways.

## Data Availability

The data that support the findings of this study are available in Gene Expression Omnibus at https://www.ncbi.nlm.nih.gov/geo/. These data were derived from the following resources available in the public domain: GSE205013, https://www.ncbi.nlm.nih.gov/geo/query/acc.cgi?acc=GSE205013; GSE197177, https://www.ncbi.nlm.nih.gov/geo/query/acc.cgi?acc=GSE197177.
