# Supplementary material for: Single‐Cell Transcriptomic Analysis Identifies a Novel OLR1 + SLC7A7 + Liver‐Enriched Metastatic Subset With Immunometabolic Rewiring in Pancreatic Cancer
Source: Cancer Med. 2025 Nov 2;14(21):e71345. doi: 10.1002/cam4.71345 (PMC12579899; doi:10.1002/cam4.71345)
Supplement: Supplementary file 1 — Figure S1: Data processing. (A, B) The UMAP plots respectively illustrating the integration of different GEO datasets before (A) and after (B) batch effects correction through the Harmony algorithm. Figure S2: The enrichment of immune response‐activating signaling pathways in C1. Bubble plot showing the activation of immune response‐activating signaling pathways in C1 analyzed by GO enrichment analysis. Figure S3: The biological function of 13 distinct epithelial clusters. Bubble plot illustrating the different pathways enriched in each epithelial cluster. Figure S4: GO analysis of downregulated genes in the differentiation trajectory of malignant cells. The downregulated genes in the differentiation trajectory of malignant cells were associated with pathways related to cell proliferation and development analyzed by GO analysis. Figure S5: The expression of OLR1 and SLC7A7 in pancreatic cancer patients. (A)The expression of OLR1 in pancreatic cancer tissues and normal tissues. (B) The expression of OLR1 at different stages of pancreatic cancer. (C) The expression of SLC7A7 in pancreatic cancer tissues and normal tissues. (D) The expression of SLC7A7 at different stages of pancreatic cancer. Figure S6: The expression of M1 and M2 related signature genes in SPP1+ macrophages. Ridgeplot showing the expression of M1 and M2 related signature genes in SPP1+ macrophages between PT and LM. Figure S7: Cell‐chat of LEMS and immune cells between PT and LM. (A)The important differential receptor‐ligand pairs from LEMS to NK cells in PT and LM. (B) The important differential receptor‐ligand pairs from LEMS to T cells in PT and LM. The color of the dot reflects the communication probability, with blank indicating a communication probability of zero. And the size of the dot corresponds to the p‐value. Figure S8: Cell‐chat of SPP1+ macrophages and 13 epithelial clusters between PT and LM. The important differential receptor‐ligand pairs from SPP1+ macrophages to distinct epithelial [file CAM4-14-e71345-s005.docx]

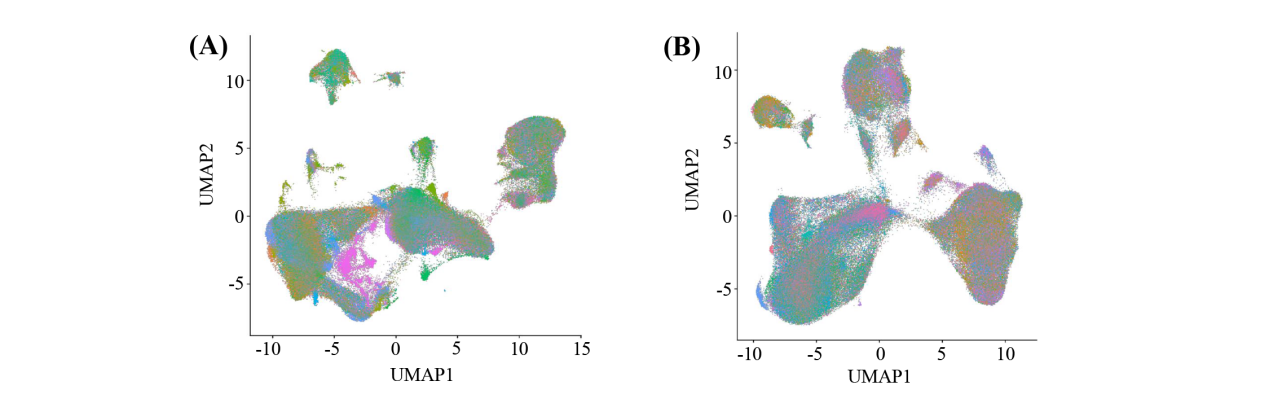


**Supplementary Figure 1. Data processing.** (A-B) The UMAP plots respectively illustrating the integration of different GEO datasets before (A) and after (B) batch effects correction through the Harmony algorithm.


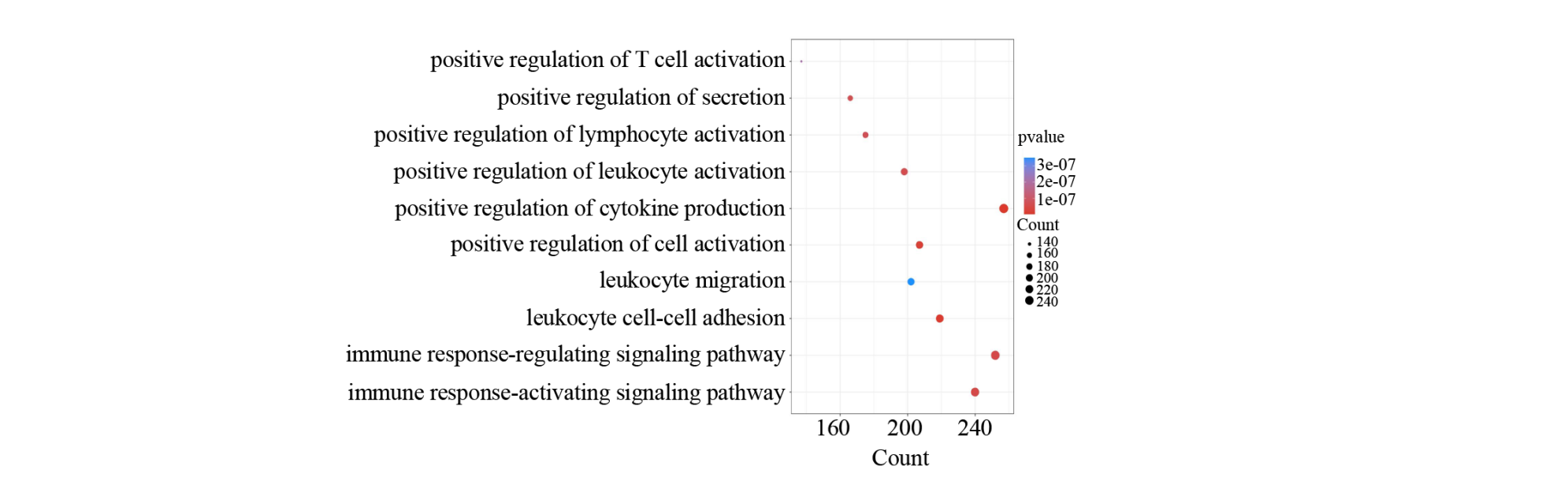
**Supplementary Figure 2. The enrichment of immune response-activating signaling pathways in C1.** Bubble plot showing the activation of immune response-activating signaling pathways in C1 analyzed by GO enrichment analysis.


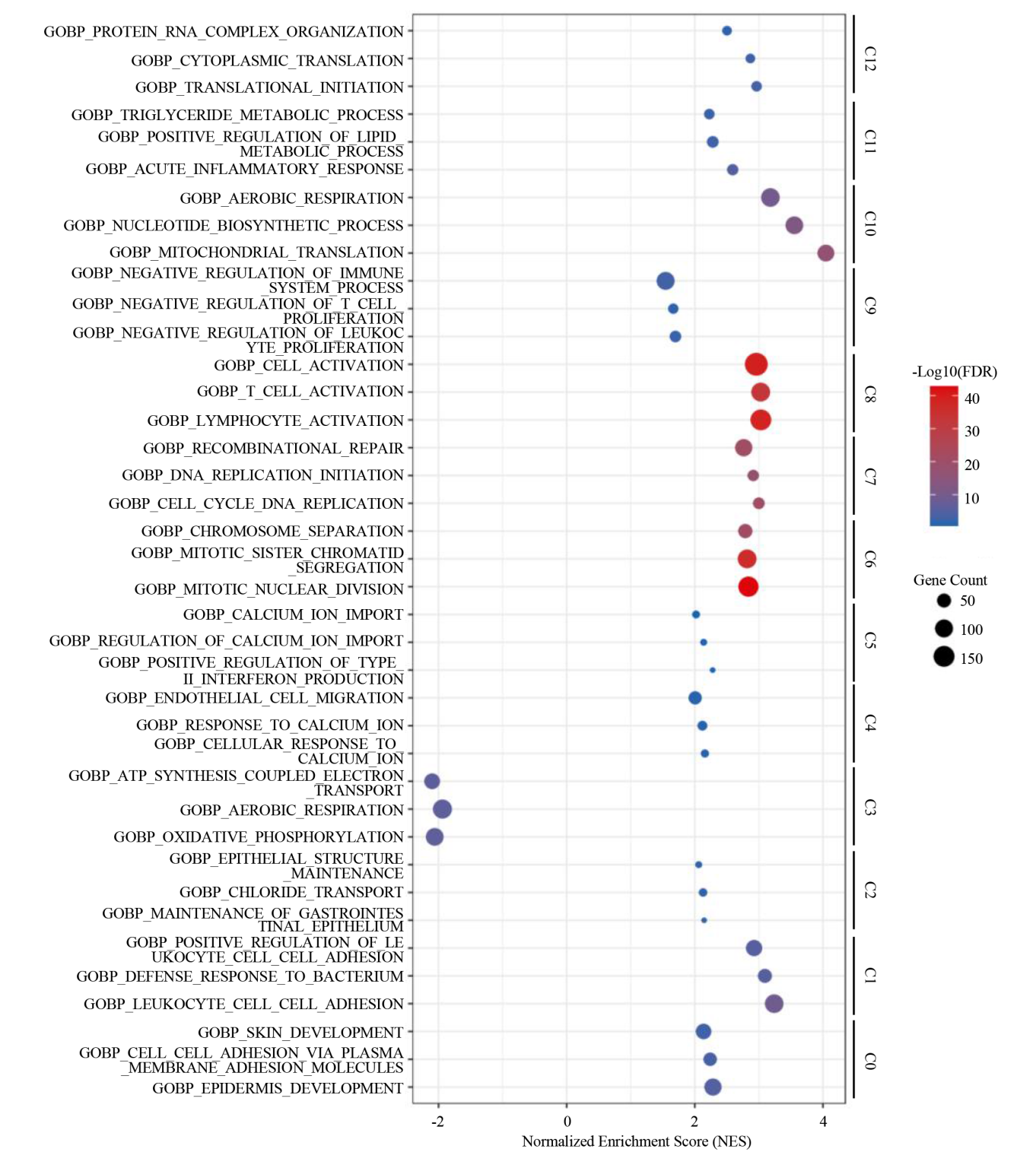
**Supplementary Figure 3. The biological function of 13 distinct epithelial clusters.** Bubble plot illustrating the different pathways enriched in each epithelial cluster.


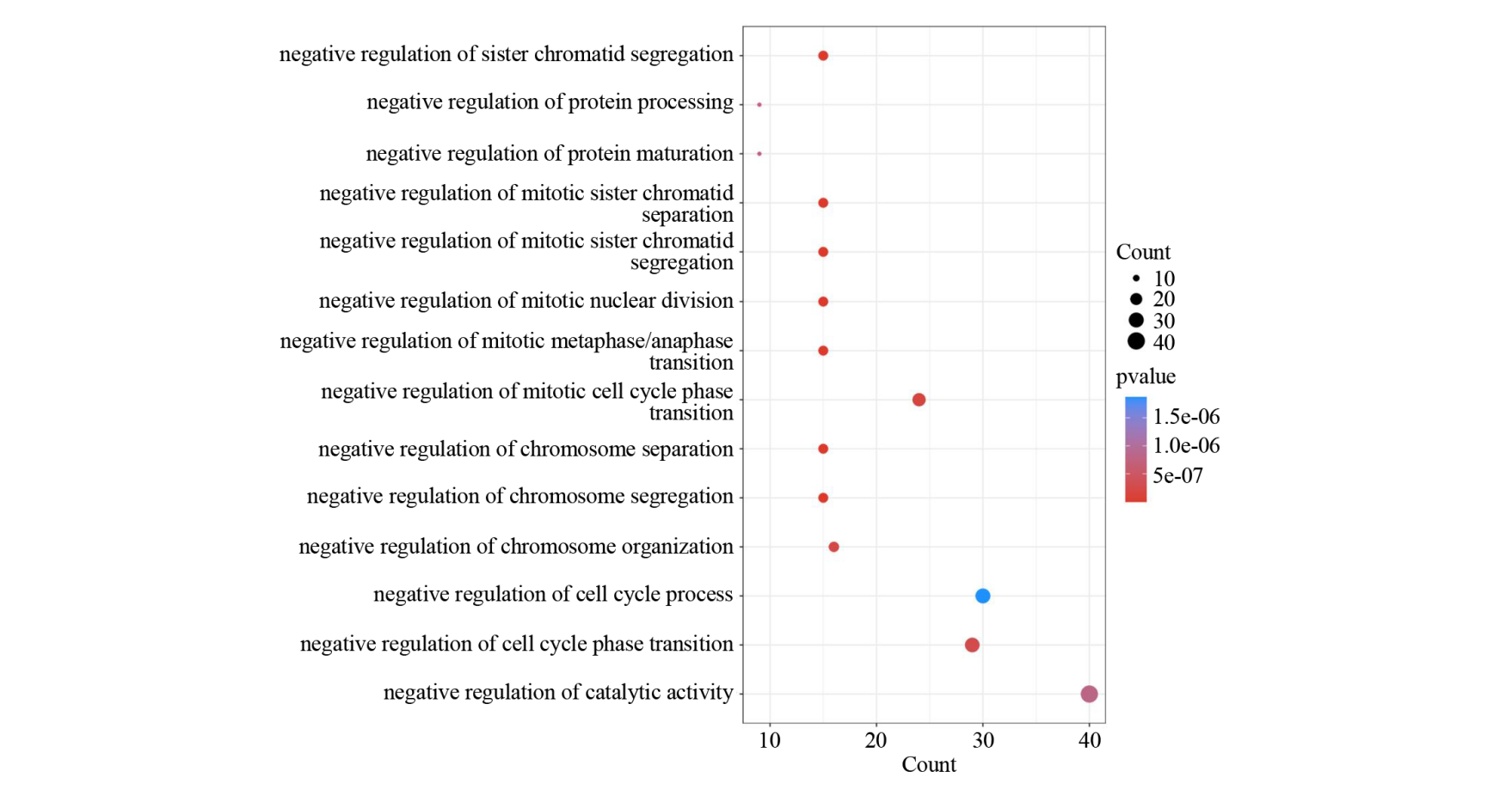


**Supplementary Figure 4. GO analysis of downregulated genes in the differentiation trajectory of malignant cells.** The downregulated genes in the differentiation trajectory of malignant cells were associated with pathways related to cell proliferation and development analyzed by GO analysis.


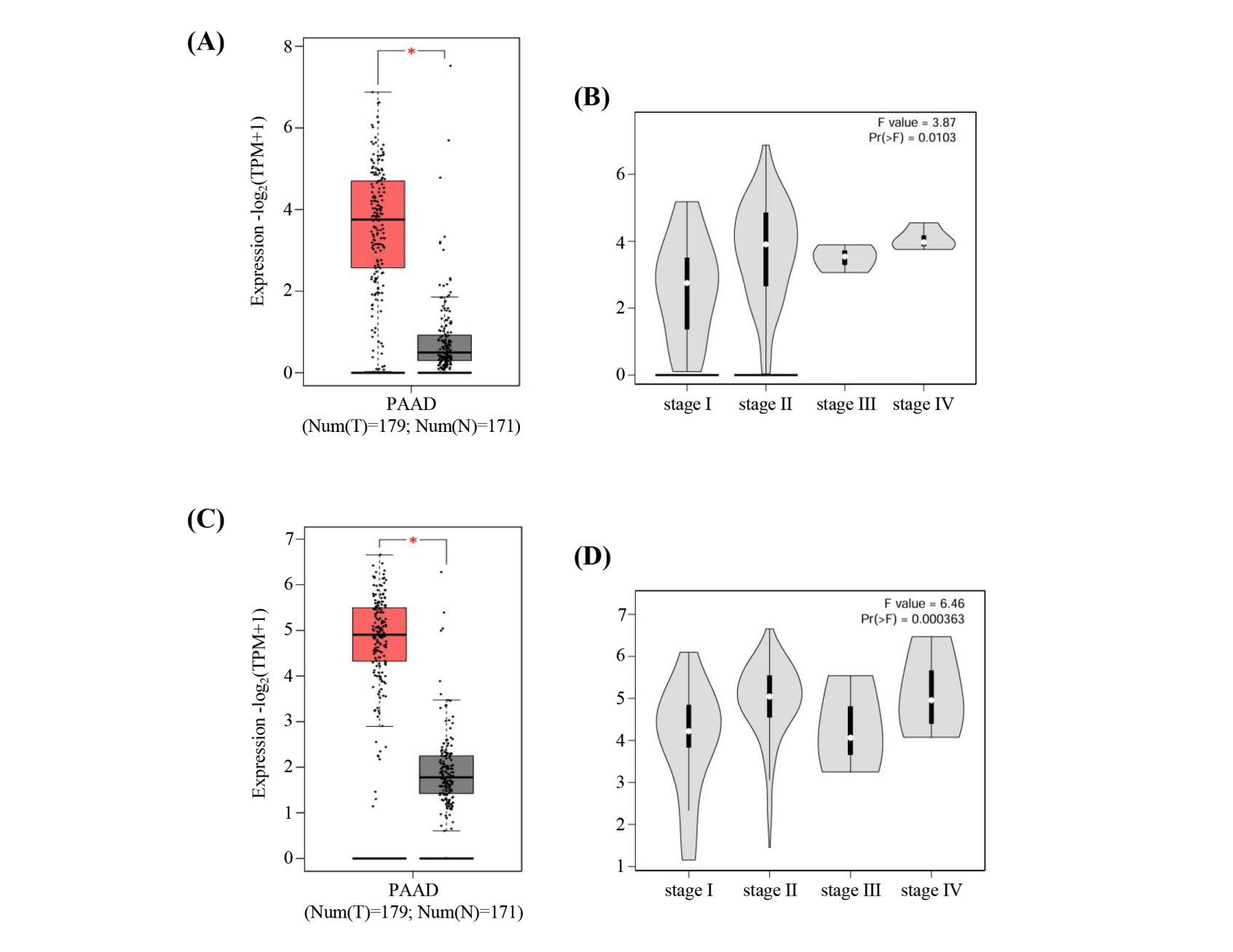
**Supplementary Figure 5. The expression of OLR1 and SLC7A7 in pancreatic cancer patients.** (A) The expression of OLR1 in pancreatic cancer tissues and normal tissues. (B) The expression of OLR1 at different stages of pancreatic cancer. (C) The expression of SLC7A7 in pancreatic cancer tissues and normal tissues. (D) he expression of SLC7A7 at different stages of pancreatic cancer.


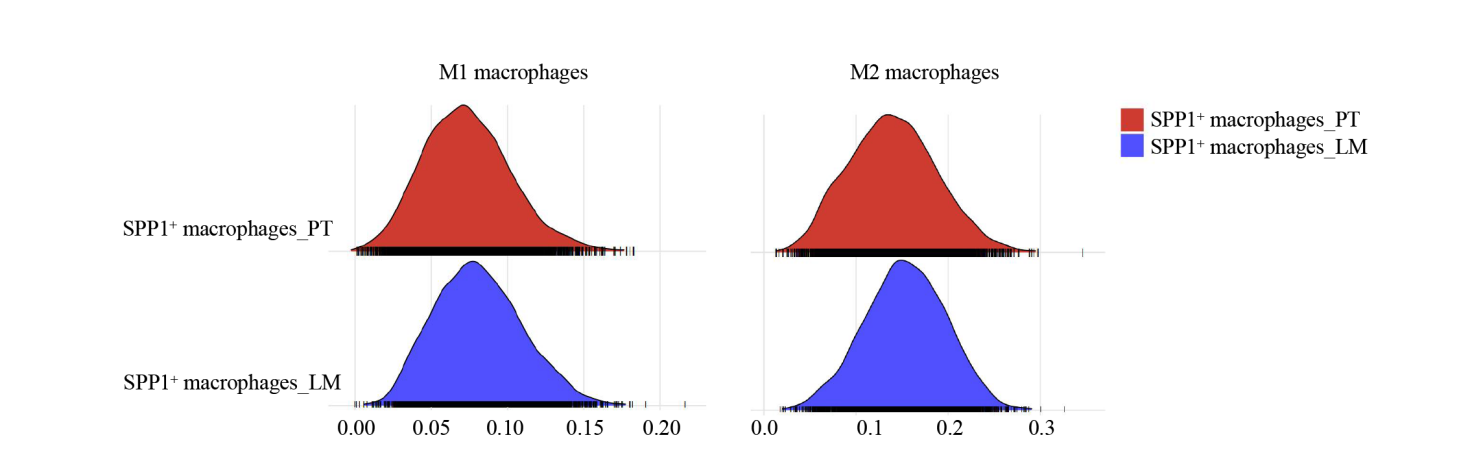


**Supplementary Figure 6. The expression of M1 and M2 related signature genes in SPP1^+^ macrophages.** Ridgeplot showing the expression of M1 and M2 related signature genes in SPP1^+^ macrophages between PT and LM.


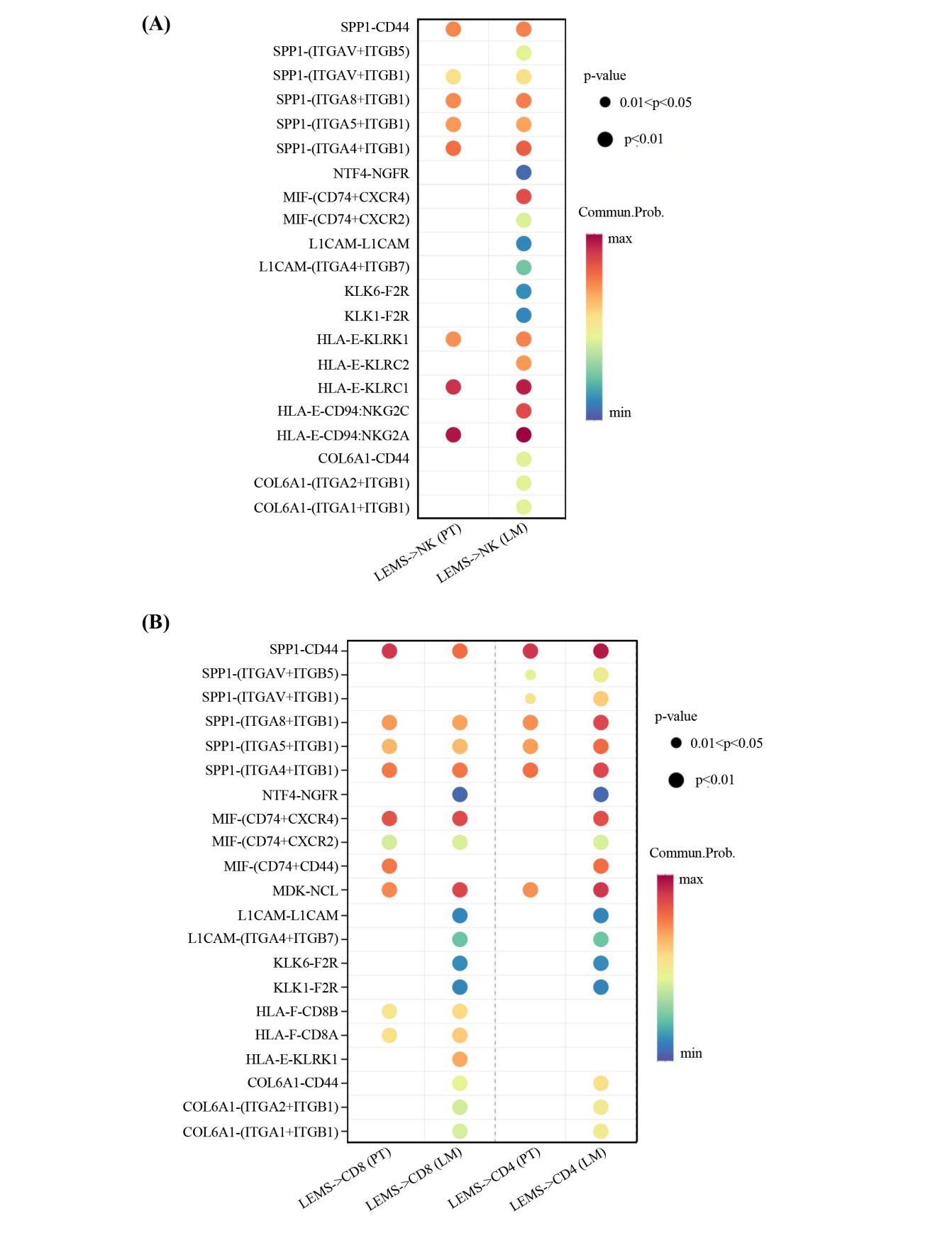
**Supplementary Figure 7. Cell-chat of LEMS and immune cells between PT and LM.** (A) The important differential receptor-ligand pairs from LEMS to NK cells in PT and LM. (B) The important differential receptor-ligand pairs from LEMS to T cells in PT and LM. The color of the dot reflects the communication probability, with blank indicating a communication probability of zero. And the size of the dot corresponds to the p-value.


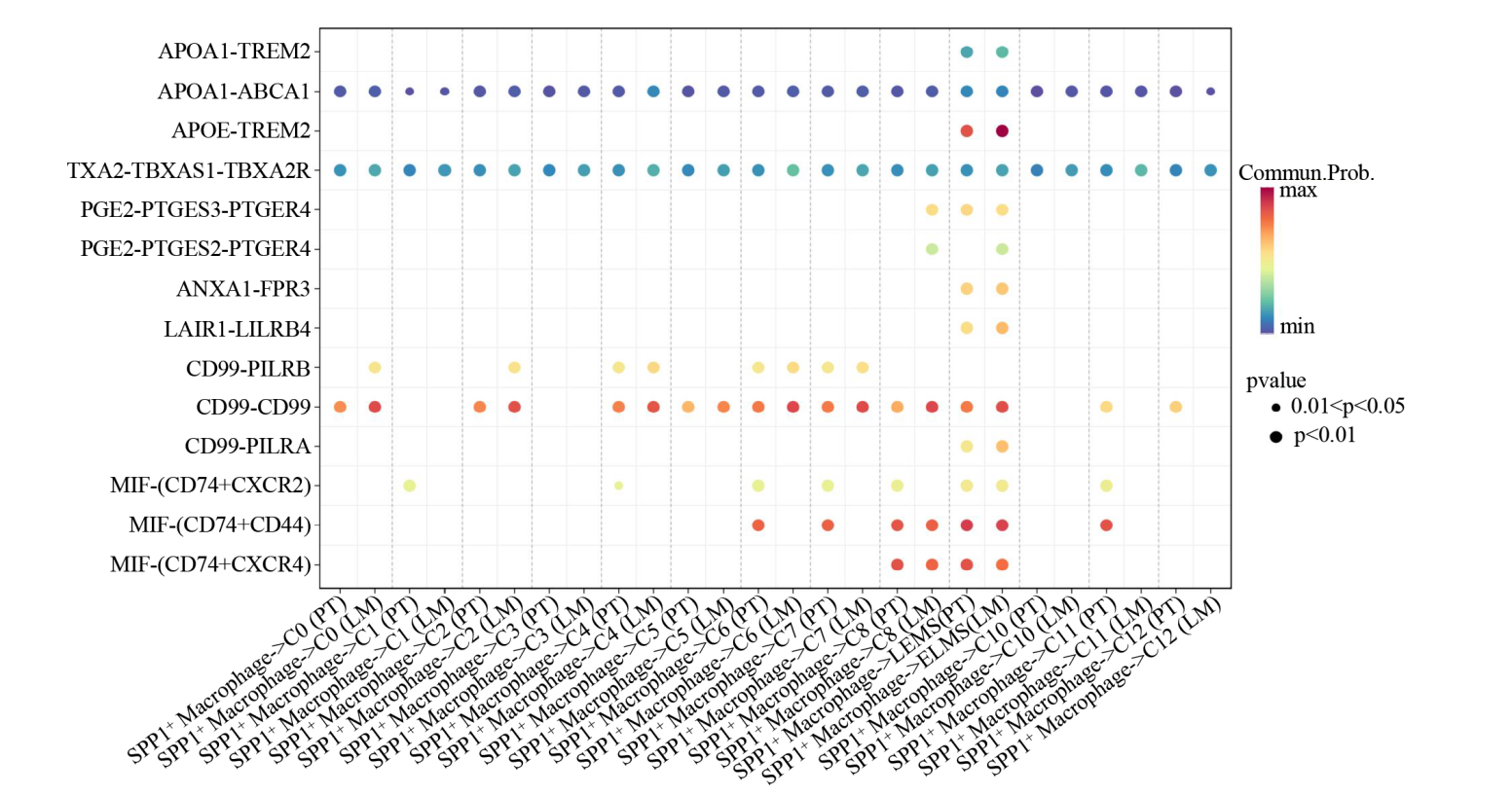
**Supplementary Figure 8. Cell-chat of SPP1^+^ macrophages and 13 epithelial clusters between PT and LM.** The important differential receptor-ligand pairs from SPP1^+^ macrophages to distinct epithelial clusters in PT and LM. The color of the dot reflects the communication probability, with blank indicating a communication probability of zero. And the size of the dot corresponds to the p-value.
