# Supplementary material for: Single‐Cell Transcriptomic Analysis Identifies a Novel OLR1 + SLC7A7 + Liver‐Enriched Metastatic Subset With Immunometabolic Rewiring in Pancreatic Cancer
Source: Cancer Med. 2025 Nov 2;14(21):e71345. doi: 10.1002/cam4.71345 (PMC12579899; doi:10.1002/cam4.71345)
Supplement: Supplementary file 2 — Table S1: The raw processed data of CopyKAT results for malignant and non‐malignant epithelial cells. [file CAM4-14-e71345-s001.docx]

| **Supplementary Table 1: The raw processed data of CopyKAT results for malignant and non-malignant epithelial cells** | | |
| --- | --- | --- |
|  | **cell.names** | **copykat.pred** |
| AAAGGGCTCGCCAGAC-1_3_1 | AAAGGGCTCGCCAGAC.1_3_1 | aneuploid |
| AACAACCCACTCAGAT-1_3_1 | AACAACCCACTCAGAT.1_3_1 | diploid |
| AACAAGAAGCCGTAAG-1_3_1 | AACAAGAAGCCGTAAG.1_3_1 | aneuploid |
| AACAAGATCTCTCCGA-1_3_1 | AACAAGATCTCTCCGA.1_3_1 | diploid |
| AACACACGTGAATGAT-1_3_1 | AACACACGTGAATGAT.1_3_1 | diploid |
| AACCAACAGACGAAGA-1_3_1 | AACCAACAGACGAAGA.1_3_1 | diploid |
| AACCCAATCGCACTCT-1_3_1 | AACCCAATCGCACTCT.1_3_1 | aneuploid |
| AACCTGAGTACTGGGA-1_3_1 | AACCTGAGTACTGGGA.1_3_1 | diploid |
| AACCTTTCAGCGACAA-1_3_1 | AACCTTTCAGCGACAA.1_3_1 | diploid |
| AACGGGACAACAGCCC-1_3_1 | AACGGGACAACAGCCC.1_3_1 | diploid |
| AACGTCACACTCACTC-1_3_1 | AACGTCACACTCACTC.1_3_1 | diploid |
| AAGAACACAGCGCGTT-1_3_1 | AAGAACACAGCGCGTT.1_3_1 | diploid |
| AAGACTCAGTTGTCGT-1_3_1 | AAGACTCAGTTGTCGT.1_3_1 | diploid |
| AAGACTCGTCTGTGAT-1_3_1 | AAGACTCGTCTGTGAT.1_3_1 | diploid |
| AAGCATCCAAGTCCCG-1_3_1 | AAGCATCCAAGTCCCG.1_3_1 | aneuploid |
| AAGCATCGTCTGTCCT-1_3_1 | AAGCATCGTCTGTCCT.1_3_1 | aneuploid |
| AAGCCATGTAAGAACT-1_3_1 | AAGCCATGTAAGAACT.1_3_1 | diploid |
| AAGCGAGGTGTGATGG-1_3_1 | AAGCGAGGTGTGATGG.1_3_1 | diploid |
| AAGTGAAAGTGCTACT-1_3_1 | AAGTGAAAGTGCTACT.1_3_1 | diploid |
| AAGTGAAGTAAGATAC-1_3_1 | AAGTGAAGTAAGATAC.1_3_1 | aneuploid |
| AAGTTCGAGGCCCGTT-1_3_1 | AAGTTCGAGGCCCGTT.1_3_1 | aneuploid |
| AATAGAGCAGGCACAA-1_3_1 | AATAGAGCAGGCACAA.1_3_1 | diploid |
| AATAGAGGTATACAGA-1_3_1 | AATAGAGGTATACAGA.1_3_1 | aneuploid |
| AATCGACAGCGTCAGA-1_3_1 | AATCGACAGCGTCAGA.1_3_1 | aneuploid |
| AATCGTGAGTACCCTA-1_3_1 | AATCGTGAGTACCCTA.1_3_1 | diploid |
| AATCGTGGTATCGGTT-1_3_1 | AATCGTGGTATCGGTT.1_3_1 | diploid |
| AATGAAGCACTGTGAT-1_3_1 | AATGAAGCACTGTGAT.1_3_1 | diploid |
| AATGAAGTCTACCAGA-1_3_1 | AATGAAGTCTACCAGA.1_3_1 | diploid |
| AATGACCCAAAGGGTC-1_3_1 | AATGACCCAAAGGGTC.1_3_1 | diploid |
| AATGGAATCGTCGCTT-1_3_1 | AATGGAATCGTCGCTT.1_3_1 | diploid |
| AATGGCTCATTGTCGA-1_3_1 | AATGGCTCATTGTCGA.1_3_1 | aneuploid |
| AATGGCTTCCGCGAGT-1_3_1 | AATGGCTTCCGCGAGT.1_3_1 | diploid |
| ACAAAGAGTTCTTGTT-1_3_1 | ACAAAGAGTTCTTGTT.1_3_1 | diploid |
| ACAAAGATCCTTTGAT-1_3_1 | ACAAAGATCCTTTGAT.1_3_1 | diploid |
| ACAAAGATCTTCTGGC-1_3_1 | ACAAAGATCTTCTGGC.1_3_1 | aneuploid |
| ACAACCACACAAGGTG-1_3_1 | ACAACCACACAAGGTG.1_3_1 | diploid |
| ACAGCCGCAAACCATC-1_3_1 | ACAGCCGCAAACCATC.1_3_1 | diploid |
| ACAGGGAAGGCACGAT-1_3_1 | ACAGGGAAGGCACGAT.1_3_1 | aneuploid |
| ACAGGGAGTAGTTCCA-1_3_1 | ACAGGGAGTAGTTCCA.1_3_1 | diploid |
| ACATCCCCATTGAGGG-1_3_1 | ACATCCCCATTGAGGG.1_3_1 | diploid |
| ACCAAACAGATACCAA-1_3_1 | ACCAAACAGATACCAA.1_3_1 | diploid |
| ACCACAAAGAGTAACT-1_3_1 | ACCACAAAGAGTAACT.1_3_1 | diploid |
| ACCACAAGTTTGCCGG-1_3_1 | ACCACAAGTTTGCCGG.1_3_1 | aneuploid |
| ACGATGTTCAGGAGAC-1_3_1 | ACGATGTTCAGGAGAC.1_3_1 | diploid |
| ACGGTTACAGGACTAG-1_3_1 | ACGGTTACAGGACTAG.1_3_1 | diploid |
| ACGGTTATCACCTGTC-1_3_1 | ACGGTTATCACCTGTC.1_3_1 | diploid |
| ACGGTTATCCGGACGT-1_3_1 | ACGGTTATCCGGACGT.1_3_1 | diploid |
| ACGTAACCAAGTTTGC-1_3_1 | ACGTAACCAAGTTTGC.1_3_1 | diploid |
| ACGTAACTCGGCAGTC-1_3_1 | ACGTAACTCGGCAGTC.1_3_1 | aneuploid |
| ACGTAGTAGTAGAGTT-1_3_1 | ACGTAGTAGTAGAGTT.1_3_1 | aneuploid |
| ACGTAGTAGTTGGCTT-1_3_1 | ACGTAGTAGTTGGCTT.1_3_1 | aneuploid |
| ACGTTCCAGCAACAAT-1_3_1 | ACGTTCCAGCAACAAT.1_3_1 | diploid |
| ACGTTCCCATGAGTAA-1_3_1 | ACGTTCCCATGAGTAA.1_3_1 | diploid |
| ACTATGGAGCAGGTCA-1_3_1 | ACTATGGAGCAGGTCA.1_3_1 | aneuploid |
| ACTATGGAGTAGATCA-1_3_1 | ACTATGGAGTAGATCA.1_3_1 | diploid |
| ACTCCCAAGCAGCCTC-1_3_1 | ACTCCCAAGCAGCCTC.1_3_1 | diploid |
| ACTCCCAGTCCTCATC-1_3_1 | ACTCCCAGTCCTCATC.1_3_1 | aneuploid |
| ACTCCCAGTGCCTAAT-1_3_1 | ACTCCCAGTGCCTAAT.1_3_1 | aneuploid |
| ACTCTCGGTAGACAAT-1_3_1 | ACTCTCGGTAGACAAT.1_3_1 | aneuploid |
| ACTCTCGTCTAGGCCG-1_3_1 | ACTCTCGTCTAGGCCG.1_3_1 | aneuploid |
| ACTGATGCAGGCGAAT-1_3_1 | ACTGATGCAGGCGAAT.1_3_1 | aneuploid |
| ACTGTGAGTGACATCT-1_3_1 | ACTGTGAGTGACATCT.1_3_1 | diploid |
| ACTTAGGTCGTGTTCC-1_3_1 | ACTTAGGTCGTGTTCC.1_3_1 | diploid |
| ACTTCCGAGAACGTGC-1_3_1 | ACTTCCGAGAACGTGC.1_3_1 | diploid |
| ACTTCCGAGCTCTTCC-1_3_1 | ACTTCCGAGCTCTTCC.1_3_1 | diploid |
| ACTTCCGCACATACGT-1_3_1 | ACTTCCGCACATACGT.1_3_1 | diploid |
| ACTTCGCCAATGCTCA-1_3_1 | ACTTCGCCAATGCTCA.1_3_1 | diploid |
| ACTTTGTCATCATTGG-1_3_1 | ACTTTGTCATCATTGG.1_3_1 | diploid |
| ACTTTGTTCAGACCGC-1_3_1 | ACTTTGTTCAGACCGC.1_3_1 | aneuploid |
| AGAAATGGTGGCCTCA-1_3_1 | AGAAATGGTGGCCTCA.1_3_1 | diploid |
| AGAACAAGTAGTATAG-1_3_1 | AGAACAAGTAGTATAG.1_3_1 | diploid |
| AGAAGTATCCAGCTCT-1_3_1 | AGAAGTATCCAGCTCT.1_3_1 | diploid |
| AGACAAAGTTCGTACA-1_3_1 | AGACAAAGTTCGTACA.1_3_1 | diploid |
| AGACACTGTCGCATCG-1_3_1 | AGACACTGTCGCATCG.1_3_1 | diploid |
| AGACCATAGATGAATC-1_3_1 | AGACCATAGATGAATC.1_3_1 | diploid |
| AGACCATGTCCATAGT-1_3_1 | AGACCATGTCCATAGT.1_3_1 | diploid |
| AGAGAATGTATCGTTG-1_3_1 | AGAGAATGTATCGTTG.1_3_1 | diploid |
| AGAGCCCGTCGCCACA-1_3_1 | AGAGCCCGTCGCCACA.1_3_1 | aneuploid |
| AGCATCAAGATAGTGT-1_3_1 | AGCATCAAGATAGTGT.1_3_1 | diploid |
| AGCATCATCCCTCGAT-1_3_1 | AGCATCATCCCTCGAT.1_3_1 | diploid |
| AGCTACACAGAAATCA-1_3_1 | AGCTACACAGAAATCA.1_3_1 | diploid |
| AGGAAATTCAAATGAG-1_3_1 | AGGAAATTCAAATGAG.1_3_1 | diploid |
| AGGAATAAGGTACAAT-1_3_1 | AGGAATAAGGTACAAT.1_3_1 | diploid |
| AGGACGAGTTTCCCAC-1_3_1 | AGGACGAGTTTCCCAC.1_3_1 | aneuploid |
| AGGACTTTCGTTTACT-1_3_1 | AGGACTTTCGTTTACT.1_3_1 | aneuploid |
| AGGATCTAGACTACGG-1_3_1 | AGGATCTAGACTACGG.1_3_1 | aneuploid |
| AGGCCACTCACTCGAA-1_3_1 | AGGCCACTCACTCGAA.1_3_1 | diploid |
| AGGGCTCGTTGCTTGA-1_3_1 | AGGGCTCGTTGCTTGA.1_3_1 | diploid |
| AGGGTCCTCTCTCAAT-1_3_1 | AGGGTCCTCTCTCAAT.1_3_1 | aneuploid |
| AGGGTGACAGAAGCTG-1_3_1 | AGGGTGACAGAAGCTG.1_3_1 | diploid |
| AGGGTGACAGATTTCG-1_3_1 | AGGGTGACAGATTTCG.1_3_1 | diploid |
| AGGTAGGTCCGCATAA-1_3_1 | AGGTAGGTCCGCATAA.1_3_1 | diploid |
| AGGTTGTGTGCATGTT-1_3_1 | AGGTTGTGTGCATGTT.1_3_1 | aneuploid |
| AGTAACCTCCCAGGCA-1_3_1 | AGTAACCTCCCAGGCA.1_3_1 | diploid |
| AGTACTGCATCACCAA-1_3_1 | AGTACTGCATCACCAA.1_3_1 | diploid |
| AGTACTGTCCTAAGTG-1_3_1 | AGTACTGTCCTAAGTG.1_3_1 | aneuploid |
| AGTACTGTCTCAGTCC-1_3_1 | AGTACTGTCTCAGTCC.1_3_1 | aneuploid |
| AGTCACAGTGTAGGAC-1_3_1 | AGTCACAGTGTAGGAC.1_3_1 | diploid |
| AGTCTCCAGGCTTCCG-1_3_1 | AGTCTCCAGGCTTCCG.1_3_1 | diploid |
| AGTGACTGTCACAATC-1_3_1 | AGTGACTGTCACAATC.1_3_1 | diploid |
| AGTGATCGTACAAGCG-1_3_1 | AGTGATCGTACAAGCG.1_3_1 | aneuploid |
| AGTGTTGTCAATGCAC-1_3_1 | AGTGTTGTCAATGCAC.1_3_1 | diploid |
| AGTGTTGTCTGCGATA-1_3_1 | AGTGTTGTCTGCGATA.1_3_1 | diploid |
| AGTTAGCTCCCAAGCG-1_3_1 | AGTTAGCTCCCAAGCG.1_3_1 | aneuploid |
| AGTTAGCTCGCACGGT-1_3_1 | AGTTAGCTCGCACGGT.1_3_1 | diploid |
| ATACCTTGTTTCGTGA-1_3_1 | ATACCTTGTTTCGTGA.1_3_1 | diploid |
| ATAGACCAGCACCAGA-1_3_1 | ATAGACCAGCACCAGA.1_3_1 | diploid |
| ATAGAGAAGTGGTTCT-1_3_1 | ATAGAGAAGTGGTTCT.1_3_1 | diploid |
| ATCACGACAGGAATCG-1_3_1 | ATCACGACAGGAATCG.1_3_1 | diploid |
| ATCATTCCACACCAGC-1_3_1 | ATCATTCCACACCAGC.1_3_1 | diploid |
| ATCCTATCACGCTTAA-1_3_1 | ATCCTATCACGCTTAA.1_3_1 | aneuploid |
| ATCCTATGTCTGATCA-1_3_1 | ATCCTATGTCTGATCA.1_3_1 | diploid |
| ATCGGCGCAGAAGTGC-1_3_1 | ATCGGCGCAGAAGTGC.1_3_1 | diploid |
| ATCGTAGAGGTACAGC-1_3_1 | ATCGTAGAGGTACAGC.1_3_1 | aneuploid |
| ATCGTGACATCTGGGC-1_3_1 | ATCGTGACATCTGGGC.1_3_1 | aneuploid |
| ATCTCTAAGTCGCCAC-1_3_1 | ATCTCTAAGTCGCCAC.1_3_1 | diploid |
| ATCTCTACAGCTGCCA-1_3_1 | ATCTCTACAGCTGCCA.1_3_1 | diploid |
| ATCTCTATCTGAGATC-1_3_1 | ATCTCTATCTGAGATC.1_3_1 | diploid |
| ATGAAAGAGAGTCTTC-1_3_1 | ATGAAAGAGAGTCTTC.1_3_1 | diploid |
| ATGAGTCAGCACTCAT-1_3_1 | ATGAGTCAGCACTCAT.1_3_1 | aneuploid |
| ATGAGTCGTGACTATC-1_3_1 | ATGAGTCGTGACTATC.1_3_1 | aneuploid |
| ATGCATGGTTGGCCGT-1_3_1 | ATGCATGGTTGGCCGT.1_3_1 | aneuploid |
| ATGCGATCATAGGCGA-1_3_1 | ATGCGATCATAGGCGA.1_3_1 | diploid |
| ATGCGATGTTCGATTG-1_3_1 | ATGCGATGTTCGATTG.1_3_1 | diploid |
| ATGGAGGGTAACATAG-1_3_1 | ATGGAGGGTAACATAG.1_3_1 | aneuploid |
| ATGGAGGGTCCAGCAC-1_3_1 | ATGGAGGGTCCAGCAC.1_3_1 | diploid |
| ATGGAGGGTCTCGGGT-1_3_1 | ATGGAGGGTCTCGGGT.1_3_1 | aneuploid |
| ATGGTTGTCCTTCTAA-1_3_1 | ATGGTTGTCCTTCTAA.1_3_1 | diploid |
| ATGTCTTTCACTGTTT-1_3_1 | ATGTCTTTCACTGTTT.1_3_1 | diploid |
| ATTACTCTCGTTGTAG-1_3_1 | ATTACTCTCGTTGTAG.1_3_1 | diploid |
| ATTCATCCAGTATACC-1_3_1 | ATTCATCCAGTATACC.1_3_1 | aneuploid |
| ATTCCTAAGGCCTAGA-1_3_1 | ATTCCTAAGGCCTAGA.1_3_1 | diploid |
| ATTCCTATCCAATGCA-1_3_1 | ATTCCTATCCAATGCA.1_3_1 | diploid |
| ATTCCTATCCATACAG-1_3_1 | ATTCCTATCCATACAG.1_3_1 | diploid |
| ATTCTACAGGTCATCT-1_3_1 | ATTCTACAGGTCATCT.1_3_1 | diploid |
| ATTCTTGCAGCGAACA-1_3_1 | ATTCTTGCAGCGAACA.1_3_1 | diploid |
| ATTCTTGCATTGCTGA-1_3_1 | ATTCTTGCATTGCTGA.1_3_1 | diploid |
| ATTCTTGGTCTTCCGT-1_3_1 | ATTCTTGGTCTTCCGT.1_3_1 | aneuploid |
| ATTGGGTTCCGTGCGA-1_3_1 | ATTGGGTTCCGTGCGA.1_3_1 | aneuploid |
| ATTTACCGTTTCACTT-1_3_1 | ATTTACCGTTTCACTT.1_3_1 | diploid |
| CAACAACCACCAGCTG-1_3_1 | CAACAACCACCAGCTG.1_3_1 | diploid |
| CAACAACCAGACAAGC-1_3_1 | CAACAACCAGACAAGC.1_3_1 | diploid |
| CAACCAATCGGTCAGC-1_3_1 | CAACCAATCGGTCAGC.1_3_1 | diploid |
| CAAGCTACACAAGCTT-1_3_1 | CAAGCTACACAAGCTT.1_3_1 | aneuploid |
| CAAGCTATCACTGCTC-1_3_1 | CAAGCTATCACTGCTC.1_3_1 | diploid |
| CAAGCTATCAGCTTGA-1_3_1 | CAAGCTATCAGCTTGA.1_3_1 | aneuploid |
| CAATGACCAGGTTCGC-1_3_1 | CAATGACCAGGTTCGC.1_3_1 | diploid |
| CAATGACTCCCGAGGT-1_3_1 | CAATGACTCCCGAGGT.1_3_1 | diploid |
| CACAACACACGACGTC-1_3_1 | CACAACACACGACGTC.1_3_1 | diploid |
| CACACAAAGAGTACCG-1_3_1 | CACACAAAGAGTACCG.1_3_1 | diploid |
| CACACAACAATAGGAT-1_3_1 | CACACAACAATAGGAT.1_3_1 | diploid |
| CACAGATAGTATGATG-1_3_1 | CACAGATAGTATGATG.1_3_1 | diploid |
| CACAGATTCTCGTCGT-1_3_1 | CACAGATTCTCGTCGT.1_3_1 | aneuploid |
| CACAGGCAGCATAGGC-1_3_1 | CACAGGCAGCATAGGC.1_3_1 | diploid |
| CACAGGCAGTAGATCA-1_3_1 | CACAGGCAGTAGATCA.1_3_1 | diploid |
| CACATGAAGGAGGTTC-1_3_1 | CACATGAAGGAGGTTC.1_3_1 | aneuploid |
| CACATGATCGTGGACC-1_3_1 | CACATGATCGTGGACC.1_3_1 | aneuploid |
| CACCAAATCCGTGCGA-1_3_1 | CACCAAATCCGTGCGA.1_3_1 | diploid |
| CACCGTTCAGTCGGAA-1_3_1 | CACCGTTCAGTCGGAA.1_3_1 | aneuploid |
| CACGAATGTGGAATGC-1_3_1 | CACGAATGTGGAATGC.1_3_1 | aneuploid |
| CACGGGTCATCATCTT-1_3_1 | CACGGGTCATCATCTT.1_3_1 | diploid |
| CACGTGGGTTATCTTC-1_3_1 | CACGTGGGTTATCTTC.1_3_1 | diploid |
| CACGTTCCAGGTCTCG-1_3_1 | CACGTTCCAGGTCTCG.1_3_1 | diploid |
| CACTGAATCACTGGTA-1_3_1 | CACTGAATCACTGGTA.1_3_1 | diploid |
| CACTGTCCAAATCGGG-1_3_1 | CACTGTCCAAATCGGG.1_3_1 | aneuploid |
| CACTGTCGTCTACAGT-1_3_1 | CACTGTCGTCTACAGT.1_3_1 | diploid |
| CACTTCGTCCGCAGTG-1_3_1 | CACTTCGTCCGCAGTG.1_3_1 | aneuploid |
| CAGATACTCATGCATG-1_3_1 | CAGATACTCATGCATG.1_3_1 | diploid |
| CAGCAGCGTCGCATTA-1_3_1 | CAGCAGCGTCGCATTA.1_3_1 | aneuploid |
| CAGCCAGGTTGCTCAA-1_3_1 | CAGCCAGGTTGCTCAA.1_3_1 | diploid |
| CAGTTAGAGCAACCAG-1_3_1 | CAGTTAGAGCAACCAG.1_3_1 | diploid |
| CATAAGCAGTCGCGAA-1_3_1 | CATAAGCAGTCGCGAA.1_3_1 | aneuploid |
| CATACAGAGGTACAAT-1_3_1 | CATACAGAGGTACAAT.1_3_1 | aneuploid |
| CATACAGTCAGTCATG-1_3_1 | CATACAGTCAGTCATG.1_3_1 | diploid |
| CATACTTCAGGAAGTC-1_3_1 | CATACTTCAGGAAGTC.1_3_1 | aneuploid |
| CATCAAGGTCTTCATT-1_3_1 | CATCAAGGTCTTCATT.1_3_1 | diploid |
| CATCCCACAAGGAGTC-1_3_1 | CATCCCACAAGGAGTC.1_3_1 | diploid |
| CATCCCAGTGTGAATA-1_3_1 | CATCCCAGTGTGAATA.1_3_1 | aneuploid |
| CATCGCTCAAGGTCAG-1_3_1 | CATCGCTCAAGGTCAG.1_3_1 | diploid |
| CATCGTCAGAGGGTGG-1_3_1 | CATCGTCAGAGGGTGG.1_3_1 | diploid |
| CATGAGTTCCTACTGC-1_3_1 | CATGAGTTCCTACTGC.1_3_1 | diploid |
| CATGCAACACAAGCTT-1_3_1 | CATGCAACACAAGCTT.1_3_1 | diploid |
| CATGCTCAGACTAAGT-1_3_1 | CATGCTCAGACTAAGT.1_3_1 | aneuploid |
| CATGGATCACGCGTGT-1_3_1 | CATGGATCACGCGTGT.1_3_1 | aneuploid |
| CATGGTATCTTTACAC-1_3_1 | CATGGTATCTTTACAC.1_3_1 | diploid |
| CATTGAGCAGGGTTGA-1_3_1 | CATTGAGCAGGGTTGA.1_3_1 | diploid |
| CCAAGCGCATATCTGG-1_3_1 | CCAAGCGCATATCTGG.1_3_1 | diploid |
| CCACAAATCTTGGTGA-1_3_1 | CCACAAATCTTGGTGA.1_3_1 | diploid |
| CCACACTCATGCGTGC-1_3_1 | CCACACTCATGCGTGC.1_3_1 | aneuploid |
| CCACGAGAGCCACCGT-1_3_1 | CCACGAGAGCCACCGT.1_3_1 | aneuploid |
| CCACGAGAGGAGGGTG-1_3_1 | CCACGAGAGGAGGGTG.1_3_1 | diploid |
| CCATAAGCAACGACTT-1_3_1 | CCATAAGCAACGACTT.1_3_1 | diploid |
| CCATCACAGAGAACCC-1_3_1 | CCATCACAGAGAACCC.1_3_1 | aneuploid |
| CCATCACAGCGTTCCG-1_3_1 | CCATCACAGCGTTCCG.1_3_1 | diploid |
| CCATCACCACTACCCT-1_3_1 | CCATCACCACTACCCT.1_3_1 | diploid |
| CCCAACTGTGCGGATA-1_3_1 | CCCAACTGTGCGGATA.1_3_1 | diploid |
| CCCATTGCAGGCGATA-1_3_1 | CCCATTGCAGGCGATA.1_3_1 | diploid |
| CCGAACGCAAACCATC-1_3_1 | CCGAACGCAAACCATC.1_3_1 | diploid |
| CCGATCTTCGTAGCTA-1_3_1 | CCGATCTTCGTAGCTA.1_3_1 | aneuploid |
| CCGCAAGGTGGAACAC-1_3_1 | CCGCAAGGTGGAACAC.1_3_1 | diploid |
| CCGCAAGGTTGCATGT-1_3_1 | CCGCAAGGTTGCATGT.1_3_1 | diploid |
| CCGCAAGTCCGTATGA-1_3_1 | CCGCAAGTCCGTATGA.1_3_1 | diploid |
| CCGGTAGGTGACTAAA-1_3_1 | CCGGTAGGTGACTAAA.1_3_1 | diploid |
| CCGGTGAAGCAGGGAG-1_3_1 | CCGGTGAAGCAGGGAG.1_3_1 | aneuploid |
| CCGGTGATCACCCTGT-1_3_1 | CCGGTGATCACCCTGT.1_3_1 | aneuploid |
| CCGTTCAAGGATTTCC-1_3_1 | CCGTTCAAGGATTTCC.1_3_1 | diploid |
| CCTAACCGTCTGATCA-1_3_1 | CCTAACCGTCTGATCA.1_3_1 | diploid |
| CCTACGTAGATCCGAG-1_3_1 | CCTACGTAGATCCGAG.1_3_1 | diploid |
| CCTATCGGTAGTGTGG-1_3_1 | CCTATCGGTAGTGTGG.1_3_1 | diploid |
| CCTCAACAGCTGCGAA-1_3_1 | CCTCAACAGCTGCGAA.1_3_1 | aneuploid |
| CCTCAACTCCCGAAAT-1_3_1 | CCTCAACTCCCGAAAT.1_3_1 | diploid |
| CCTCAACTCCTGGGAC-1_3_1 | CCTCAACTCCTGGGAC.1_3_1 | diploid |
| CCTCATGAGTCAGCGA-1_3_1 | CCTCATGAGTCAGCGA.1_3_1 | diploid |
| CCTCCAAAGCGACTGA-1_3_1 | CCTCCAAAGCGACTGA.1_3_1 | diploid |
| CCTCCTCAGGTAAACT-1_3_1 | CCTCCTCAGGTAAACT.1_3_1 | diploid |
| CCTCTAGAGTTGCGAG-1_3_1 | CCTCTAGAGTTGCGAG.1_3_1 | diploid |
| CCTCTCCCAAACACCT-1_3_1 | CCTCTCCCAAACACCT.1_3_1 | diploid |
| CCTGTTGCAGGTATGG-1_3_1 | CCTGTTGCAGGTATGG.1_3_1 | diploid |
| CCTTCAGTCTAGGCCG-1_3_1 | CCTTCAGTCTAGGCCG.1_3_1 | diploid |
| CCTTTGGAGACGAGCT-1_3_1 | CCTTTGGAGACGAGCT.1_3_1 | aneuploid |
| CCTTTGGTCCAAGAGG-1_3_1 | CCTTTGGTCCAAGAGG.1_3_1 | diploid |
| CGAAGGACATCATTTC-1_3_1 | CGAAGGACATCATTTC.1_3_1 | diploid |
| CGAAGTTAGCAATTCC-1_3_1 | CGAAGTTAGCAATTCC.1_3_1 | diploid |
| CGACAGCAGTGTTCAC-1_3_1 | CGACAGCAGTGTTCAC.1_3_1 | aneuploid |
| CGACAGCTCCATCGTC-1_3_1 | CGACAGCTCCATCGTC.1_3_1 | aneuploid |
| CGAGGAAGTATAATGG-1_3_1 | CGAGGAAGTATAATGG.1_3_1 | diploid |
| CGAGTTATCTGAACGT-1_3_1 | CGAGTTATCTGAACGT.1_3_1 | diploid |
| CGATCGGCACTGGATT-1_3_1 | CGATCGGCACTGGATT.1_3_1 | diploid |
| CGATGGCCAACGTAAA-1_3_1 | CGATGGCCAACGTAAA.1_3_1 | diploid |
| CGATGGCGTTCCGCAG-1_3_1 | CGATGGCGTTCCGCAG.1_3_1 | aneuploid |
| CGCAGGTGTGCCCAGT-1_3_1 | CGCAGGTGTGCCCAGT.1_3_1 | aneuploid |
| CGCATAACATGGGAAC-1_3_1 | CGCATAACATGGGAAC.1_3_1 | diploid |
| CGCATAATCTCAACCC-1_3_1 | CGCATAATCTCAACCC.1_3_1 | diploid |
| CGCATGGTCGGATACT-1_3_1 | CGCATGGTCGGATACT.1_3_1 | diploid |
| CGCCATTAGTCCCAGC-1_3_1 | CGCCATTAGTCCCAGC.1_3_1 | aneuploid |
| CGGGACTCAAACTCTG-1_3_1 | CGGGACTCAAACTCTG.1_3_1 | diploid |
| CGGGACTCACGACAAG-1_3_1 | CGGGACTCACGACAAG.1_3_1 | diploid |
| CGGGTGTCAGTTTGGT-1_3_1 | CGGGTGTCAGTTTGGT.1_3_1 | diploid |
| CGGTCAGTCCAAGCTA-1_3_1 | CGGTCAGTCCAAGCTA.1_3_1 | diploid |
| CGTAAGTCAACTGATC-1_3_1 | CGTAAGTCAACTGATC.1_3_1 | aneuploid |
| CGTCAAACAAGATGGC-1_3_1 | CGTCAAACAAGATGGC.1_3_1 | diploid |
| CGTGTCTAGCTGAGCA-1_3_1 | CGTGTCTAGCTGAGCA.1_3_1 | diploid |
| CGTGTCTTCACCTGGG-1_3_1 | CGTGTCTTCACCTGGG.1_3_1 | aneuploid |
| CGTTAGACAGGTTTAC-1_3_1 | CGTTAGACAGGTTTAC.1_3_1 | diploid |
| CGTTGGGAGGTACAGC-1_3_1 | CGTTGGGAGGTACAGC.1_3_1 | diploid |
| CTAAGTGAGCCTCATA-1_3_1 | CTAAGTGAGCCTCATA.1_3_1 | aneuploid |
| CTAAGTGCATGACCCG-1_3_1 | CTAAGTGCATGACCCG.1_3_1 | diploid |
| CTACGGGGTTATGTCG-1_3_1 | CTACGGGGTTATGTCG.1_3_1 | diploid |
| CTACTATTCACCATGA-1_3_1 | CTACTATTCACCATGA.1_3_1 | diploid |
| CTAGACACAAGTGGGT-1_3_1 | CTAGACACAAGTGGGT.1_3_1 | aneuploid |
| CTAGGTACACGACGTC-1_3_1 | CTAGGTACACGACGTC.1_3_1 | aneuploid |
| CTATCTATCCTAAACG-1_3_1 | CTATCTATCCTAAACG.1_3_1 | diploid |
| CTCAAGAAGCAGGCTA-1_3_1 | CTCAAGAAGCAGGCTA.1_3_1 | diploid |
| CTCAAGACAGCCTATA-1_3_1 | CTCAAGACAGCCTATA.1_3_1 | aneuploid |
| CTCAATTAGCCTGCCA-1_3_1 | CTCAATTAGCCTGCCA.1_3_1 | diploid |
| CTCAATTCATCGATAC-1_3_1 | CTCAATTCATCGATAC.1_3_1 | diploid |
| CTCACTGCATCATTGG-1_3_1 | CTCACTGCATCATTGG.1_3_1 | aneuploid |
| CTCAGTCGTCATAACC-1_3_1 | CTCAGTCGTCATAACC.1_3_1 | diploid |
| CTCATCGAGCATCTTG-1_3_1 | CTCATCGAGCATCTTG.1_3_1 | diploid |
| CTCCATGTCTGAGGCC-1_3_1 | CTCCATGTCTGAGGCC.1_3_1 | diploid |
| CTCCCTCCAACTGATC-1_3_1 | CTCCCTCCAACTGATC.1_3_1 | diploid |
| CTCCCTCTCAGCTCTC-1_3_1 | CTCCCTCTCAGCTCTC.1_3_1 | diploid |
| CTCCCTCTCTGCGAGC-1_3_1 | CTCCCTCTCTGCGAGC.1_3_1 | aneuploid |
| CTCTCAGAGAGTCAAT-1_3_1 | CTCTCAGAGAGTCAAT.1_3_1 | aneuploid |
| CTCTCAGCAGCTACTA-1_3_1 | CTCTCAGCAGCTACTA.1_3_1 | aneuploid |
| CTCTGGTAGTTAGTAG-1_3_1 | CTCTGGTAGTTAGTAG.1_3_1 | diploid |
| CTGAATGAGCAGATAT-1_3_1 | CTGAATGAGCAGATAT.1_3_1 | aneuploid |
| CTGAGGCCAAAGGGTC-1_3_1 | CTGAGGCCAAAGGGTC.1_3_1 | diploid |
| CTGATCCGTATCCCTC-1_3_1 | CTGATCCGTATCCCTC.1_3_1 | diploid |
| CTGATCCTCTCTTAAC-1_3_1 | CTGATCCTCTCTTAAC.1_3_1 | aneuploid |
| CTGCAGGCAACGATCT-1_3_1 | CTGCAGGCAACGATCT.1_3_1 | aneuploid |
| CTGCTCAAGCTGTACT-1_3_1 | CTGCTCAAGCTGTACT.1_3_1 | diploid |
| CTGGACGAGTGCAACG-1_3_1 | CTGGACGAGTGCAACG.1_3_1 | aneuploid |
| CTGGCAGGTCGTTGCG-1_3_1 | CTGGCAGGTCGTTGCG.1_3_1 | diploid |
| CTGGTCTAGTAACAGT-1_3_1 | CTGGTCTAGTAACAGT.1_3_1 | aneuploid |
| CTGGTCTCAACAAGAT-1_3_1 | CTGGTCTCAACAAGAT.1_3_1 | diploid |
| CTGTACCCACCGCTGA-1_3_1 | CTGTACCCACCGCTGA.1_3_1 | diploid |
| CTGTAGAGTGTGAGCA-1_3_1 | CTGTAGAGTGTGAGCA.1_3_1 | diploid |
| CTGTGGGCAGGTGAGT-1_3_1 | CTGTGGGCAGGTGAGT.1_3_1 | aneuploid |
| CTTCAATGTAACGCGA-1_3_1 | CTTCAATGTAACGCGA.1_3_1 | diploid |
| CTTCCTTGTGTGCCTG-1_3_1 | CTTCCTTGTGTGCCTG.1_3_1 | diploid |
| CTTCCTTTCTGCACCT-1_3_1 | CTTCCTTTCTGCACCT.1_3_1 | aneuploid |
| CTTCTAAAGTAGTCCT-1_3_1 | CTTCTAAAGTAGTCCT.1_3_1 | diploid |
| CTTCTCTCACGCGTCA-1_3_1 | CTTCTCTCACGCGTCA.1_3_1 | aneuploid |
| CTTCTCTCACGTAACT-1_3_1 | CTTCTCTCACGTAACT.1_3_1 | diploid |
| CTTGATTTCATTCCTA-1_3_1 | CTTGATTTCATTCCTA.1_3_1 | diploid |
| CTTTCAACAACCAACT-1_3_1 | CTTTCAACAACCAACT.1_3_1 | aneuploid |
| GAAACCTGTGCAGTGA-1_3_1 | GAAACCTGTGCAGTGA.1_3_1 | diploid |
| GAACTGTGTTCAGGTT-1_3_1 | GAACTGTGTTCAGGTT.1_3_1 | diploid |
| GAAGCCCCAAGAGCTG-1_3_1 | GAAGCCCCAAGAGCTG.1_3_1 | diploid |
| GAAGCGAAGAACAGGA-1_3_1 | GAAGCGAAGAACAGGA.1_3_1 | aneuploid |
| GAAGGGTTCCATCGTC-1_3_1 | GAAGGGTTCCATCGTC.1_3_1 | aneuploid |
| GAAGTAAAGGTAAAGG-1_3_1 | GAAGTAAAGGTAAAGG.1_3_1 | diploid |
| GAAGTAAGTAGTCCTA-1_3_1 | GAAGTAAGTAGTCCTA.1_3_1 | diploid |
| GACACGCCAACAAGTA-1_3_1 | GACACGCCAACAAGTA.1_3_1 | diploid |
| GACATCACATCGGAAG-1_3_1 | GACATCACATCGGAAG.1_3_1 | diploid |
| GACCCAGCAGTCAGAG-1_3_1 | GACCCAGCAGTCAGAG.1_3_1 | diploid |
| GACCCAGCAGTGTGCC-1_3_1 | GACCCAGCAGTGTGCC.1_3_1 | diploid |
| GACCGTGTCTCGGTAA-1_3_1 | GACCGTGTCTCGGTAA.1_3_1 | diploid |
| GACGTTATCCTACAAG-1_3_1 | GACGTTATCCTACAAG.1_3_1 | diploid |
| GACGTTATCTGAGAAA-1_3_1 | GACGTTATCTGAGAAA.1_3_1 | aneuploid |
| GACTATGCAACTCCAA-1_3_1 | GACTATGCAACTCCAA.1_3_1 | aneuploid |
| GACTATGGTCGATTTG-1_3_1 | GACTATGGTCGATTTG.1_3_1 | diploid |
| GACTCAACACAAGCAG-1_3_1 | GACTCAACACAAGCAG.1_3_1 | diploid |
| GACTCAACAGGCTCTG-1_3_1 | GACTCAACAGGCTCTG.1_3_1 | aneuploid |
| GACTGATCAAAGGGTC-1_3_1 | GACTGATCAAAGGGTC.1_3_1 | aneuploid |
| GAGACCCTCAACTGGT-1_3_1 | GAGACCCTCAACTGGT.1_3_1 | aneuploid |
| GAGCCTGTCTCCGATC-1_3_1 | GAGCCTGTCTCCGATC.1_3_1 | diploid |
| GAGCTGCGTCAACCAT-1_3_1 | GAGCTGCGTCAACCAT.1_3_1 | aneuploid |
| GAGCTGCTCACGATCA-1_3_1 | GAGCTGCTCACGATCA.1_3_1 | aneuploid |
| GAGGCAAAGAGTCTGG-1_3_1 | GAGGCAAAGAGTCTGG.1_3_1 | diploid |
| GAGGGATAGGGTATAT-1_3_1 | GAGGGATAGGGTATAT.1_3_1 | aneuploid |
| GAGGGATCAACACAGG-1_3_1 | GAGGGATCAACACAGG.1_3_1 | diploid |
| GAGGGATCAGAGTTCT-1_3_1 | GAGGGATCAGAGTTCT.1_3_1 | diploid |
| GAGTCTACATGAGGGT-1_3_1 | GAGTCTACATGAGGGT.1_3_1 | aneuploid |
| GAGTTACGTATGTCTG-1_3_1 | GAGTTACGTATGTCTG.1_3_1 | diploid |
| GAGTTGTAGATGATTG-1_3_1 | GAGTTGTAGATGATTG.1_3_1 | diploid |
| GAGTTGTCACAGTGAG-1_3_1 | GAGTTGTCACAGTGAG.1_3_1 | aneuploid |
| GAGTTTGAGGATACGC-1_3_1 | GAGTTTGAGGATACGC.1_3_1 | aneuploid |
| GAGTTTGCACAAATAG-1_3_1 | GAGTTTGCACAAATAG.1_3_1 | diploid |
| GATAGAAAGGATGCGT-1_3_1 | GATAGAAAGGATGCGT.1_3_1 | diploid |
| GATAGCTGTGTCTTAG-1_3_1 | GATAGCTGTGTCTTAG.1_3_1 | diploid |
| GATCCCTCAAACTCTG-1_3_1 | GATCCCTCAAACTCTG.1_3_1 | diploid |
| GATCGTACACCCTCTA-1_3_1 | GATCGTACACCCTCTA.1_3_1 | diploid |
| GATGACTGTGACTGAG-1_3_1 | GATGACTGTGACTGAG.1_3_1 | aneuploid |
| GATGAGGTCGCGCCAA-1_3_1 | GATGAGGTCGCGCCAA.1_3_1 | aneuploid |
| GATGCTAGTAAGTCAA-1_3_1 | GATGCTAGTAAGTCAA.1_3_1 | aneuploid |
| GATGTTGAGTGCAAAT-1_3_1 | GATGTTGAGTGCAAAT.1_3_1 | diploid |
| GCAACATCACGGAAGT-1_3_1 | GCAACATCACGGAAGT.1_3_1 | aneuploid |
| GCACATAAGGCCCGTT-1_3_1 | GCACATAAGGCCCGTT.1_3_1 | diploid |
| GCACATATCACTAGCA-1_3_1 | GCACATATCACTAGCA.1_3_1 | aneuploid |
| GCACATATCCGGTTCT-1_3_1 | GCACATATCCGGTTCT.1_3_1 | diploid |
| GCACGGTTCGTTCAGA-1_3_1 | GCACGGTTCGTTCAGA.1_3_1 | aneuploid |
| GCACTAACAGCAGTGA-1_3_1 | GCACTAACAGCAGTGA.1_3_1 | aneuploid |
| GCAGCCAAGCACGATG-1_3_1 | GCAGCCAAGCACGATG.1_3_1 | diploid |
| GCAGCCACAAACTGCT-1_3_1 | GCAGCCACAAACTGCT.1_3_1 | aneuploid |
| GCAGCTGAGTGAACAT-1_3_1 | GCAGCTGAGTGAACAT.1_3_1 | diploid |
| GCAGCTGTCGGCTGAC-1_3_1 | GCAGCTGTCGGCTGAC.1_3_1 | diploid |
| GCAGTTAAGCTACTAC-1_3_1 | GCAGTTAAGCTACTAC.1_3_1 | diploid |
| GCATGATAGAAACTAC-1_3_1 | GCATGATAGAAACTAC.1_3_1 | diploid |
| GCCAGTGGTTGTTTGG-1_3_1 | GCCAGTGGTTGTTTGG.1_3_1 | diploid |
| GCCATTCTCGGTCTAA-1_3_1 | GCCATTCTCGGTCTAA.1_3_1 | aneuploid |
| GCCCAGAAGGCAGGGA-1_3_1 | GCCCAGAAGGCAGGGA.1_3_1 | diploid |
| GCCGTGACAGGCGTTC-1_3_1 | GCCGTGACAGGCGTTC.1_3_1 | diploid |
| GCCGTGAGTTATGACC-1_3_1 | GCCGTGAGTTATGACC.1_3_1 | diploid |
| GCGAGAATCCGGTAGC-1_3_1 | GCGAGAATCCGGTAGC.1_3_1 | diploid |
| GCGATCGGTGTTTGCA-1_3_1 | GCGATCGGTGTTTGCA.1_3_1 | diploid |
| GCTACAATCGGTTGTA-1_3_1 | GCTACAATCGGTTGTA.1_3_1 | aneuploid |
| GCTCAAAGTACCCACG-1_3_1 | GCTCAAAGTACCCACG.1_3_1 | diploid |
| GCTGAATGTCACTCGG-1_3_1 | GCTGAATGTCACTCGG.1_3_1 | diploid |
| GCTTCACGTTTGGGAG-1_3_1 | GCTTCACGTTTGGGAG.1_3_1 | aneuploid |
| GGAACCCCAACGTTAC-1_3_1 | GGAACCCCAACGTTAC.1_3_1 | aneuploid |
| GGAAGTGCACCTGTCT-1_3_1 | GGAAGTGCACCTGTCT.1_3_1 | aneuploid |
| GGAATGGAGATGAACT-1_3_1 | GGAATGGAGATGAACT.1_3_1 | diploid |
| GGAATGGCAAAGCTAA-1_3_1 | GGAATGGCAAAGCTAA.1_3_1 | diploid |
| GGAGATGAGTAGGATT-1_3_1 | GGAGATGAGTAGGATT.1_3_1 | diploid |
| GGAGCAAGTACTCGCG-1_3_1 | GGAGCAAGTACTCGCG.1_3_1 | diploid |
| GGAGGATCACGAAGAC-1_3_1 | GGAGGATCACGAAGAC.1_3_1 | diploid |
| GGAGGATTCGTTCAGA-1_3_1 | GGAGGATTCGTTCAGA.1_3_1 | diploid |
| GGAGGTACAAATCAAG-1_3_1 | GGAGGTACAAATCAAG.1_3_1 | diploid |
| GGATCTAAGGGCTGAT-1_3_1 | GGATCTAAGGGCTGAT.1_3_1 | aneuploid |
| GGATCTAGTGGTTTGT-1_3_1 | GGATCTAGTGGTTTGT.1_3_1 | aneuploid |
| GGATCTATCAGCCTTC-1_3_1 | GGATCTATCAGCCTTC.1_3_1 | diploid |
| GGATGTTGTTCGGCGT-1_3_1 | GGATGTTGTTCGGCGT.1_3_1 | diploid |
| GGATGTTTCTACTATC-1_3_1 | GGATGTTTCTACTATC.1_3_1 | diploid |
| GGCAGTCGTATATGGA-1_3_1 | GGCAGTCGTATATGGA.1_3_1 | diploid |
| GGCTGTGAGGCGTCCT-1_3_1 | GGCTGTGAGGCGTCCT.1_3_1 | diploid |
| GGCTGTGGTGAATTGA-1_3_1 | GGCTGTGGTGAATTGA.1_3_1 | aneuploid |
| GGCTTGGCATCCGTTC-1_3_1 | GGCTTGGCATCCGTTC.1_3_1 | diploid |
| GGCTTTCAGCCTGCCA-1_3_1 | GGCTTTCAGCCTGCCA.1_3_1 | diploid |
| GGGACAACACCTGAAT-1_3_1 | GGGACAACACCTGAAT.1_3_1 | diploid |
| GGGACAACATTGAGGG-1_3_1 | GGGACAACATTGAGGG.1_3_1 | diploid |
| GGGACTCAGGGTTAGC-1_3_1 | GGGACTCAGGGTTAGC.1_3_1 | diploid |
| GGGAGATAGTTCATCG-1_3_1 | GGGAGATAGTTCATCG.1_3_1 | diploid |
| GGGAGTAGTATAATGG-1_3_1 | GGGAGTAGTATAATGG.1_3_1 | diploid |
| GGGATGAGTACGATTC-1_3_1 | GGGATGAGTACGATTC.1_3_1 | diploid |
| GGGCGTTAGACCTCAT-1_3_1 | GGGCGTTAGACCTCAT.1_3_1 | aneuploid |
| GGGCGTTGTGAGTTGG-1_3_1 | GGGCGTTGTGAGTTGG.1_3_1 | diploid |
| GGGTAGAAGAACCCGA-1_3_1 | GGGTAGAAGAACCCGA.1_3_1 | diploid |
| GGGTATTAGCGAGAAA-1_3_1 | GGGTATTAGCGAGAAA.1_3_1 | diploid |
| GGGTCTGCATCCTAAG-1_3_1 | GGGTCTGCATCCTAAG.1_3_1 | diploid |
| GGTAACTAGTATGAAC-1_3_1 | GGTAACTAGTATGAAC.1_3_1 | aneuploid |
| GGTAATCCACTCACTC-1_3_1 | GGTAATCCACTCACTC.1_3_1 | aneuploid |
| GGTGAAGTCCCGGTAG-1_3_1 | GGTGAAGTCCCGGTAG.1_3_1 | diploid |
| GGTGTCGTCTTCTTCC-1_3_1 | GGTGTCGTCTTCTTCC.1_3_1 | aneuploid |
| GGTGTTAAGGACCCAA-1_3_1 | GGTGTTAAGGACCCAA.1_3_1 | diploid |
| GGTGTTAAGTAGCCAG-1_3_1 | GGTGTTAAGTAGCCAG.1_3_1 | aneuploid |
| GTAACACGTATAGGAT-1_3_1 | GTAACACGTATAGGAT.1_3_1 | diploid |
| GTAATCGAGGACTTCT-1_3_1 | GTAATCGAGGACTTCT.1_3_1 | diploid |
| GTAATCGCATAGAGGC-1_3_1 | GTAATCGCATAGAGGC.1_3_1 | aneuploid |
| GTACAACTCAACACGT-1_3_1 | GTACAACTCAACACGT.1_3_1 | diploid |
| GTAGAGGAGATTGCGG-1_3_1 | GTAGAGGAGATTGCGG.1_3_1 | aneuploid |
| GTAGATCGTGCATACT-1_3_1 | GTAGATCGTGCATACT.1_3_1 | diploid |
| GTAGATCTCAAGTCTG-1_3_1 | GTAGATCTCAAGTCTG.1_3_1 | aneuploid |
| GTAGGTTGTGTCATGT-1_3_1 | GTAGGTTGTGTCATGT.1_3_1 | diploid |
| GTAGTACTCTGGGATT-1_3_1 | GTAGTACTCTGGGATT.1_3_1 | diploid |
| GTATTGGGTCACTACA-1_3_1 | GTATTGGGTCACTACA.1_3_1 | aneuploid |
| GTATTTCGTATCAGGG-1_3_1 | GTATTTCGTATCAGGG.1_3_1 | diploid |
| GTCAAACAGACTCTAC-1_3_1 | GTCAAACAGACTCTAC.1_3_1 | diploid |
| GTCAAACCATCTGCGG-1_3_1 | GTCAAACCATCTGCGG.1_3_1 | diploid |
| GTCAAGTTCTGCCTCA-1_3_1 | GTCAAGTTCTGCCTCA.1_3_1 | diploid |
| GTCACTCCAGCCGGTT-1_3_1 | GTCACTCCAGCCGGTT.1_3_1 | diploid |
| GTCAGCGAGCAGCCCT-1_3_1 | GTCAGCGAGCAGCCCT.1_3_1 | aneuploid |
| GTCAGCGTCGAACCTA-1_3_1 | GTCAGCGTCGAACCTA.1_3_1 | aneuploid |
| GTCCACTGTAGCTGTT-1_3_1 | GTCCACTGTAGCTGTT.1_3_1 | diploid |
| GTCCACTGTCGTCAGC-1_3_1 | GTCCACTGTCGTCAGC.1_3_1 | aneuploid |
| GTCCCATGTGAGAACC-1_3_1 | GTCCCATGTGAGAACC.1_3_1 | diploid |
| GTCCTCAAGGTTTACC-1_3_1 | GTCCTCAAGGTTTACC.1_3_1 | aneuploid |
| GTCGCGACAACCCGCA-1_3_1 | GTCGCGACAACCCGCA.1_3_1 | diploid |
| GTCGTTCAGTCGGCCT-1_3_1 | GTCGTTCAGTCGGCCT.1_3_1 | aneuploid |
| GTCTGTCTCTTACCAT-1_3_1 | GTCTGTCTCTTACCAT.1_3_1 | diploid |
| GTCTTTAAGCAATAGT-1_3_1 | GTCTTTAAGCAATAGT.1_3_1 | aneuploid |
| GTGATGTGTTCACCGG-1_3_1 | GTGATGTGTTCACCGG.1_3_1 | aneuploid |
| GTGCACGGTGTTATCG-1_3_1 | GTGCACGGTGTTATCG.1_3_1 | diploid |
| GTGCAGCCAGCTACCG-1_3_1 | GTGCAGCCAGCTACCG.1_3_1 | aneuploid |
| GTGCTGGAGTCTTCCC-1_3_1 | GTGCTGGAGTCTTCCC.1_3_1 | diploid |
| GTGCTTCGTCTACAAC-1_3_1 | GTGCTTCGTCTACAAC.1_3_1 | diploid |
| GTGCTTCTCTGCTAGA-1_3_1 | GTGCTTCTCTGCTAGA.1_3_1 | diploid |
| GTGGGAAGTTATCTGG-1_3_1 | GTGGGAAGTTATCTGG.1_3_1 | diploid |
| GTGGTTAGTATCGCGC-1_3_1 | GTGGTTAGTATCGCGC.1_3_1 | diploid |
| GTGTAACCAGACAATA-1_3_1 | GTGTAACCAGACAATA.1_3_1 | diploid |
| GTGTCCTAGGCCTAAG-1_3_1 | GTGTCCTAGGCCTAAG.1_3_1 | diploid |
| GTGTGATGTAACCAGG-1_3_1 | GTGTGATGTAACCAGG.1_3_1 | diploid |
| GTGTGGCGTGGCGTAA-1_3_1 | GTGTGGCGTGGCGTAA.1_3_1 | diploid |
| GTTACCCTCGGTTGTA-1_3_1 | GTTACCCTCGGTTGTA.1_3_1 | diploid |
| GTTACGACACTATGTG-1_3_1 | GTTACGACACTATGTG.1_3_1 | diploid |
| GTTAGTGAGCTAGAGC-1_3_1 | GTTAGTGAGCTAGAGC.1_3_1 | aneuploid |
| GTTAGTGGTACACGCC-1_3_1 | GTTAGTGGTACACGCC.1_3_1 | diploid |
| GTTATGGCATTGACTG-1_3_1 | GTTATGGCATTGACTG.1_3_1 | diploid |
| GTTCATTCACATATCG-1_3_1 | GTTCATTCACATATCG.1_3_1 | aneuploid |
| GTTCCGTTCGAGAGAC-1_3_1 | GTTCCGTTCGAGAGAC.1_3_1 | aneuploid |
| GTTCGCTTCATAGAGA-1_3_1 | GTTCGCTTCATAGAGA.1_3_1 | diploid |
| GTTCGCTTCGTGCGAC-1_3_1 | GTTCGCTTCGTGCGAC.1_3_1 | diploid |
| GTTCTATAGACCCGCT-1_3_1 | GTTCTATAGACCCGCT.1_3_1 | aneuploid |
| GTTGAACAGTAGGCCA-1_3_1 | GTTGAACAGTAGGCCA.1_3_1 | diploid |
| GTTGCTCGTCCGGACT-1_3_1 | GTTGCTCGTCCGGACT.1_3_1 | diploid |
| GTTTACTAGCTAGAAT-1_3_1 | GTTTACTAGCTAGAAT.1_3_1 | aneuploid |
| GTTTGGAGTCACTCGG-1_3_1 | GTTTGGAGTCACTCGG.1_3_1 | aneuploid |
| TAACTTCTCGACATCA-1_3_1 | TAACTTCTCGACATCA.1_3_1 | aneuploid |
| TAAGCACCAGAGTCTT-1_3_1 | TAAGCACCAGAGTCTT.1_3_1 | diploid |
| TAAGCACCATCGAGCC-1_3_1 | TAAGCACCATCGAGCC.1_3_1 | diploid |
| TAAGCACGTACGCTTA-1_3_1 | TAAGCACGTACGCTTA.1_3_1 | aneuploid |
| TAAGCCATCATAGGCT-1_3_1 | TAAGCCATCATAGGCT.1_3_1 | diploid |
| TAAGTCGTCCTACACC-1_3_1 | TAAGTCGTCCTACACC.1_3_1 | diploid |
| TAATTCCCAAGCTGCC-1_3_1 | TAATTCCCAAGCTGCC.1_3_1 | diploid |
| TACAGGTCAGAGGAAA-1_3_1 | TACAGGTCAGAGGAAA.1_3_1 | diploid |
| TACAGGTGTAACGTTC-1_3_1 | TACAGGTGTAACGTTC.1_3_1 | aneuploid |
| TACAGGTTCTCCTGAC-1_3_1 | TACAGGTTCTCCTGAC.1_3_1 | diploid |
| TACATTCCAGCAGGAT-1_3_1 | TACATTCCAGCAGGAT.1_3_1 | diploid |
| TACATTCCATTGTACG-1_3_1 | TACATTCCATTGTACG.1_3_1 | aneuploid |
| TACATTCGTTAAGCAA-1_3_1 | TACATTCGTTAAGCAA.1_3_1 | diploid |
| TACATTCTCAAATGCC-1_3_1 | TACATTCTCAAATGCC.1_3_1 | aneuploid |
| TACCGAAGTCGTCGGT-1_3_1 | TACCGAAGTCGTCGGT.1_3_1 | diploid |
| TACGCTCTCCTGATAG-1_3_1 | TACGCTCTCCTGATAG.1_3_1 | diploid |
| TACTTCATCTTCCACG-1_3_1 | TACTTCATCTTCCACG.1_3_1 | diploid |
| TAGACCAAGATAGGGA-1_3_1 | TAGACCAAGATAGGGA.1_3_1 | diploid |
| TAGAGTCAGAGTGTGC-1_3_1 | TAGAGTCAGAGTGTGC.1_3_1 | aneuploid |
| TAGAGTCTCATGGTAC-1_3_1 | TAGAGTCTCATGGTAC.1_3_1 | diploid |
| TAGATCGTCCATATGG-1_3_1 | TAGATCGTCCATATGG.1_3_1 | diploid |
| TAGATCGTCGAACGGA-1_3_1 | TAGATCGTCGAACGGA.1_3_1 | diploid |
| TAGCACACAATGCTCA-1_3_1 | TAGCACACAATGCTCA.1_3_1 | diploid |
| TAGCACACAGATCATC-1_3_1 | TAGCACACAGATCATC.1_3_1 | diploid |
| TAGCACAGTCACCGCA-1_3_1 | TAGCACAGTCACCGCA.1_3_1 | diploid |
| TAGGGTTGTACTTGTG-1_3_1 | TAGGGTTGTACTTGTG.1_3_1 | diploid |
| TAGGTACGTTTATGCG-1_3_1 | TAGGTACGTTTATGCG.1_3_1 | aneuploid |
| TATACCTAGACATAGT-1_3_1 | TATACCTAGACATAGT.1_3_1 | diploid |
| TATACCTCATGGTACT-1_3_1 | TATACCTCATGGTACT.1_3_1 | diploid |
| TATATCCTCACTACGA-1_3_1 | TATATCCTCACTACGA.1_3_1 | diploid |
| TATCTGTGTGCTTATG-1_3_1 | TATCTGTGTGCTTATG.1_3_1 | diploid |
| TATGTTCGTGTATTGC-1_3_1 | TATGTTCGTGTATTGC.1_3_1 | diploid |
| TATTCCAAGGACAAGA-1_3_1 | TATTCCAAGGACAAGA.1_3_1 | diploid |
| TATTGCTAGCTAATCC-1_3_1 | TATTGCTAGCTAATCC.1_3_1 | aneuploid |
| TATTGCTTCTAGGCCG-1_3_1 | TATTGCTTCTAGGCCG.1_3_1 | aneuploid |
| TATTGGGTCCGAAATC-1_3_1 | TATTGGGTCCGAAATC.1_3_1 | diploid |
| TATTTCGTCTGTGCTC-1_3_1 | TATTTCGTCTGTGCTC.1_3_1 | aneuploid |
| TCAAGACTCATGAGTC-1_3_1 | TCAAGACTCATGAGTC.1_3_1 | diploid |
| TCAAGCAGTATTCTCT-1_3_1 | TCAAGCAGTATTCTCT.1_3_1 | diploid |
| TCAAGTGGTTGTTTGG-1_3_1 | TCAAGTGGTTGTTTGG.1_3_1 | aneuploid |
| TCAATCTCAGGCTACC-1_3_1 | TCAATCTCAGGCTACC.1_3_1 | diploid |
| TCAATCTCAGTTCACA-1_3_1 | TCAATCTCAGTTCACA.1_3_1 | aneuploid |
| TCAATTCTCATCCTAT-1_3_1 | TCAATTCTCATCCTAT.1_3_1 | diploid |
| TCACATTCAACTTCTT-1_3_1 | TCACATTCAACTTCTT.1_3_1 | diploid |
| TCACATTCACCTGCAG-1_3_1 | TCACATTCACCTGCAG.1_3_1 | diploid |
| TCACGGGTCTCGAGTA-1_3_1 | TCACGGGTCTCGAGTA.1_3_1 | aneuploid |
| TCAGCAATCCCGTTCA-1_3_1 | TCAGCAATCCCGTTCA.1_3_1 | diploid |
| TCAGGGCGTGTTCGAT-1_3_1 | TCAGGGCGTGTTCGAT.1_3_1 | aneuploid |
| TCAGGTACATGACGGA-1_3_1 | TCAGGTACATGACGGA.1_3_1 | aneuploid |
| TCAGTCCTCATTTCCA-1_3_1 | TCAGTCCTCATTTCCA.1_3_1 | diploid |
| TCAGTCCTCCTGTAGA-1_3_1 | TCAGTCCTCCTGTAGA.1_3_1 | diploid |
| TCATATCAGTGAGTTA-1_3_1 | TCATATCAGTGAGTTA.1_3_1 | aneuploid |
| TCATGAGAGCCTGAGA-1_3_1 | TCATGAGAGCCTGAGA.1_3_1 | diploid |
| TCATGAGTCGCGCCAA-1_3_1 | TCATGAGTCGCGCCAA.1_3_1 | diploid |
| TCATGAGTCTCTATAC-1_3_1 | TCATGAGTCTCTATAC.1_3_1 | diploid |
| TCATTGTGTACCTATG-1_3_1 | TCATTGTGTACCTATG.1_3_1 | aneuploid |
| TCATTTGAGATAGTCA-1_3_1 | TCATTTGAGATAGTCA.1_3_1 | diploid |
| TCCACGTTCTAGTTCT-1_3_1 | TCCACGTTCTAGTTCT.1_3_1 | aneuploid |
| TCCAGAAAGACATCCT-1_3_1 | TCCAGAAAGACATCCT.1_3_1 | diploid |
| TCCAGAATCCTCTAAT-1_3_1 | TCCAGAATCCTCTAAT.1_3_1 | diploid |
| TCCATCGTCTCCAAGA-1_3_1 | TCCATCGTCTCCAAGA.1_3_1 | aneuploid |
| TCCATGCAGCTGCCTG-1_3_1 | TCCATGCAGCTGCCTG.1_3_1 | diploid |
| TCCATGCCACGGAAGT-1_3_1 | TCCATGCCACGGAAGT.1_3_1 | diploid |
| TCCATGCCAGTTGTTG-1_3_1 | TCCATGCCAGTTGTTG.1_3_1 | aneuploid |
| TCCATGCGTACCGCGT-1_3_1 | TCCATGCGTACCGCGT.1_3_1 | aneuploid |
| TCCATGCTCGGCATTA-1_3_1 | TCCATGCTCGGCATTA.1_3_1 | aneuploid |
| TCCCACACAAGACCGA-1_3_1 | TCCCACACAAGACCGA.1_3_1 | aneuploid |
| TCCCACAGTGATGAAT-1_3_1 | TCCCACAGTGATGAAT.1_3_1 | diploid |
| TCCCAGTAGACCTCCG-1_3_1 | TCCCAGTAGACCTCCG.1_3_1 | diploid |
| TCCCATGGTCGACGCT-1_3_1 | TCCCATGGTCGACGCT.1_3_1 | diploid |
| TCCGAAACATCGTCCT-1_3_1 | TCCGAAACATCGTCCT.1_3_1 | diploid |
| TCCTAATCATCTCAAG-1_3_1 | TCCTAATCATCTCAAG.1_3_1 | diploid |
| TCCTAATGTGGTACAG-1_3_1 | TCCTAATGTGGTACAG.1_3_1 | diploid |
| TCCTCCCAGTGCCTCG-1_3_1 | TCCTCCCAGTGCCTCG.1_3_1 | aneuploid |
| TCCTCGACACCCTGTT-1_3_1 | TCCTCGACACCCTGTT.1_3_1 | diploid |
| TCCTCGATCGAAGCAG-1_3_1 | TCCTCGATCGAAGCAG.1_3_1 | diploid |
| TCCTGCAAGCGCTGCT-1_3_1 | TCCTGCAAGCGCTGCT.1_3_1 | diploid |
| TCCTGCAGTAGGCTCC-1_3_1 | TCCTGCAGTAGGCTCC.1_3_1 | diploid |
| TCCTGCATCTGTCTCG-1_3_1 | TCCTGCATCTGTCTCG.1_3_1 | diploid |
| TCCTTCTCAAGTCGTT-1_3_1 | TCCTTCTCAAGTCGTT.1_3_1 | aneuploid |
| TCCTTTCAGCCGTTGC-1_3_1 | TCCTTTCAGCCGTTGC.1_3_1 | diploid |
| TCGACCTAGGGAGATA-1_3_1 | TCGACCTAGGGAGATA.1_3_1 | aneuploid |
| TCGACGGCAACTGCCG-1_3_1 | TCGACGGCAACTGCCG.1_3_1 | aneuploid |
| TCGCAGGGTAGCCAGA-1_3_1 | TCGCAGGGTAGCCAGA.1_3_1 | diploid |
| TCGCTCATCAGTCAGT-1_3_1 | TCGCTCATCAGTCAGT.1_3_1 | diploid |
| TCGCTTGGTGTTGCCG-1_3_1 | TCGCTTGGTGTTGCCG.1_3_1 | diploid |
| TCGGATAGTCCATACA-1_3_1 | TCGGATAGTCCATACA.1_3_1 | diploid |
| TCGGGCACAAACGTGG-1_3_1 | TCGGGCACAAACGTGG.1_3_1 | aneuploid |
| TCGGGCAGTGGATCAG-1_3_1 | TCGGGCAGTGGATCAG.1_3_1 | diploid |
| TCGGGTGTCGCTGTTC-1_3_1 | TCGGGTGTCGCTGTTC.1_3_1 | diploid |
| TCGTGGGTCACGGTCG-1_3_1 | TCGTGGGTCACGGTCG.1_3_1 | diploid |
| TCTACCGGTTGCGGCT-1_3_1 | TCTACCGGTTGCGGCT.1_3_1 | diploid |
| TCTGCCAGTGTTCCTC-1_3_1 | TCTGCCAGTGTTCCTC.1_3_1 | aneuploid |
| TCTGCCATCACCGGGT-1_3_1 | TCTGCCATCACCGGGT.1_3_1 | aneuploid |
| TCTGTCGCAGACCTAT-1_3_1 | TCTGTCGCAGACCTAT.1_3_1 | diploid |
| TCTTAGTAGGCTGTAG-1_3_1 | TCTTAGTAGGCTGTAG.1_3_1 | diploid |
| TCTTAGTCAGTGCGCT-1_3_1 | TCTTAGTCAGTGCGCT.1_3_1 | diploid |
| TCTTGCGAGTGAGGTC-1_3_1 | TCTTGCGAGTGAGGTC.1_3_1 | aneuploid |
| TCTTGCGCATGAAGGC-1_3_1 | TCTTGCGCATGAAGGC.1_3_1 | aneuploid |
| TCTTTGATCGAGTGGA-1_3_1 | TCTTTGATCGAGTGGA.1_3_1 | aneuploid |
| TGAACGTCACTATCGA-1_3_1 | TGAACGTCACTATCGA.1_3_1 | diploid |
| TGAACGTGTCGTGGAA-1_3_1 | TGAACGTGTCGTGGAA.1_3_1 | diploid |
| TGAATCGAGCTCTGTA-1_3_1 | TGAATCGAGCTCTGTA.1_3_1 | aneuploid |
| TGACAGTCACCACATA-1_3_1 | TGACAGTCACCACATA.1_3_1 | aneuploid |
| TGACAGTTCCAAACCA-1_3_1 | TGACAGTTCCAAACCA.1_3_1 | diploid |
| TGACGCGGTGGCCACT-1_3_1 | TGACGCGGTGGCCACT.1_3_1 | aneuploid |
| TGACGCGTCCCTTTGG-1_3_1 | TGACGCGTCCCTTTGG.1_3_1 | aneuploid |
| TGACTCCTCGCGCTGA-1_3_1 | TGACTCCTCGCGCTGA.1_3_1 | aneuploid |
| TGAGACTGTCGAAACG-1_3_1 | TGAGACTGTCGAAACG.1_3_1 | diploid |
| TGAGACTTCTATCGTT-1_3_1 | TGAGACTTCTATCGTT.1_3_1 | diploid |
| TGAGCATCACTGGACC-1_3_1 | TGAGCATCACTGGACC.1_3_1 | diploid |
| TGAGCATTCTCGTGGG-1_3_1 | TGAGCATTCTCGTGGG.1_3_1 | diploid |
| TGAGGTTAGTGGTGAC-1_3_1 | TGAGGTTAGTGGTGAC.1_3_1 | diploid |
| TGATGCAAGGTGCCTC-1_3_1 | TGATGCAAGGTGCCTC.1_3_1 | diploid |
| TGATGCATCTACTGCC-1_3_1 | TGATGCATCTACTGCC.1_3_1 | diploid |
| TGATTTCAGGGCCTCT-1_3_1 | TGATTTCAGGGCCTCT.1_3_1 | diploid |
| TGCACGGAGATGTTCC-1_3_1 | TGCACGGAGATGTTCC.1_3_1 | diploid |
| TGCACGGGTAGGCAAC-1_3_1 | TGCACGGGTAGGCAAC.1_3_1 | diploid |
| TGCAGGCAGTCCTGTA-1_3_1 | TGCAGGCAGTCCTGTA.1_3_1 | diploid |
| TGCAGGCGTCCGTACG-1_3_1 | TGCAGGCGTCCGTACG.1_3_1 | aneuploid |
| TGCAGGCTCTGTACAG-1_3_1 | TGCAGGCTCTGTACAG.1_3_1 | diploid |
| TGCAGTAGTGGCCCAT-1_3_1 | TGCAGTAGTGGCCCAT.1_3_1 | diploid |
| TGCAGTAGTTGTTGCA-1_3_1 | TGCAGTAGTTGTTGCA.1_3_1 | diploid |
| TGCATCCAGGTGCCAA-1_3_1 | TGCATCCAGGTGCCAA.1_3_1 | diploid |
| TGCCGAGTCCGGTAGC-1_3_1 | TGCCGAGTCCGGTAGC.1_3_1 | diploid |
| TGCGGCACACCCTATC-1_3_1 | TGCGGCACACCCTATC.1_3_1 | diploid |
| TGCGGGTTCCTTCAGC-1_3_1 | TGCGGGTTCCTTCAGC.1_3_1 | diploid |
| TGCTGAACAGCCGTTG-1_3_1 | TGCTGAACAGCCGTTG.1_3_1 | aneuploid |
| TGCTGAATCCACAAGT-1_3_1 | TGCTGAATCCACAAGT.1_3_1 | diploid |
| TGCTGAATCCTAGCTC-1_3_1 | TGCTGAATCCTAGCTC.1_3_1 | diploid |
| TGCTGAATCGAAGGAC-1_3_1 | TGCTGAATCGAAGGAC.1_3_1 | diploid |
| TGCTTCGCAAAGCAAT-1_3_1 | TGCTTCGCAAAGCAAT.1_3_1 | diploid |
| TGCTTCGCATAGGCGA-1_3_1 | TGCTTCGCATAGGCGA.1_3_1 | diploid |
| TGCTTCGTCAGTCCGG-1_3_1 | TGCTTCGTCAGTCCGG.1_3_1 | aneuploid |
| TGCTTCGTCCTGTTGC-1_3_1 | TGCTTCGTCCTGTTGC.1_3_1 | aneuploid |
| TGCTTGCGTAGTTCCA-1_3_1 | TGCTTGCGTAGTTCCA.1_3_1 | diploid |
| TGGAACTAGGAGACCT-1_3_1 | TGGAACTAGGAGACCT.1_3_1 | diploid |
| TGGAGAGAGTTCCTGA-1_3_1 | TGGAGAGAGTTCCTGA.1_3_1 | diploid |
| TGGAGGAAGGTCCAGA-1_3_1 | TGGAGGAAGGTCCAGA.1_3_1 | aneuploid |
| TGGATCAGTCCACTCT-1_3_1 | TGGATCAGTCCACTCT.1_3_1 | aneuploid |
| TGGGAAGGTGAACTAA-1_3_1 | TGGGAAGGTGAACTAA.1_3_1 | diploid |
| TGGGCTGCAATAGGGC-1_3_1 | TGGGCTGCAATAGGGC.1_3_1 | diploid |
| TGGGCTGGTTACGCCG-1_3_1 | TGGGCTGGTTACGCCG.1_3_1 | aneuploid |
| TGGGTTATCTTACCAT-1_3_1 | TGGGTTATCTTACCAT.1_3_1 | diploid |
| TGGTGATTCTGCATGA-1_3_1 | TGGTGATTCTGCATGA.1_3_1 | aneuploid |
| TGGTTAGTCGTGCACG-1_3_1 | TGGTTAGTCGTGCACG.1_3_1 | diploid |
| TGTAACGTCAACTTTC-1_3_1 | TGTAACGTCAACTTTC.1_3_1 | diploid |
| TGTAACGTCTGTCTCG-1_3_1 | TGTAACGTCTGTCTCG.1_3_1 | diploid |
| TGTAAGCCAGATTTCG-1_3_1 | TGTAAGCCAGATTTCG.1_3_1 | diploid |
| TGTACAGCAGGCATTT-1_3_1 | TGTACAGCAGGCATTT.1_3_1 | diploid |
| TGTAGACCAACCGTGC-1_3_1 | TGTAGACCAACCGTGC.1_3_1 | diploid |
| TGTCAGATCATTCTTG-1_3_1 | TGTCAGATCATTCTTG.1_3_1 | aneuploid |
| TGTCCTGTCGCTGTTC-1_3_1 | TGTCCTGTCGCTGTTC.1_3_1 | aneuploid |
| TGTGAGTCAGGCATTT-1_3_1 | TGTGAGTCAGGCATTT.1_3_1 | diploid |
| TGTGAGTGTATCGAGG-1_3_1 | TGTGAGTGTATCGAGG.1_3_1 | diploid |
| TGTGATGAGAAGCGCT-1_3_1 | TGTGATGAGAAGCGCT.1_3_1 | diploid |
| TGTGATGAGGTAAGAG-1_3_1 | TGTGATGAGGTAAGAG.1_3_1 | aneuploid |
| TGTGATGGTAACCCTA-1_3_1 | TGTGATGGTAACCCTA.1_3_1 | diploid |
| TGTGGCGAGGTTGCCC-1_3_1 | TGTGGCGAGGTTGCCC.1_3_1 | aneuploid |
| TGTGGCGCAGCTTCGG-1_3_1 | TGTGGCGCAGCTTCGG.1_3_1 | diploid |
| TGTGTGAGTATCGCGC-1_3_1 | TGTGTGAGTATCGCGC.1_3_1 | diploid |
| TGTTCATCACGAGGAT-1_3_1 | TGTTCATCACGAGGAT.1_3_1 | diploid |
| TGTTCCGCAAGCACAG-1_3_1 | TGTTCCGCAAGCACAG.1_3_1 | diploid |
| TGTTGAGCAGTAGGAC-1_3_1 | TGTTGAGCAGTAGGAC.1_3_1 | diploid |
