# Supplementary material for: Single‐Cell Transcriptomic Analysis Identifies a Novel OLR1 + SLC7A7 + Liver‐Enriched Metastatic Subset With Immunometabolic Rewiring in Pancreatic Cancer
Source: Cancer Med. 2025 Nov 2;14(21):e71345. doi: 10.1002/cam4.71345 (PMC12579899; doi:10.1002/cam4.71345)
Supplement: Supplementary file 3 — Table S2: The different receptor‐ligand pairs between LEMS and macrophages. [file CAM4-14-e71345-s002.docx]

| **Supplementary Table 2 : The different receptor-ligand pairs between LEMS and macrophages** | | | | | | | | | | | | |
| --- | --- | --- | --- | --- | --- | --- | --- | --- | --- | --- | --- | --- |
|  | **source** | **target** | **ligand** | **receptor** | **prob** | **pval** | **interaction_name** | **interaction_name_2** | **pathway_name** | **annotation** | **evidence** | **group** |
| 1 | FOLR2 Macrophage | FOLR2 Macrophage | TGFB1 | TGFbR1_R2 | 0.000518502 | 0 | TGFB1_TGFBR1_TGFBR2 | TGFB1 - (TGFBR1+TGFBR2) | TGFb | Secreted Signaling | KEGG: hsa04350 | LM |
| 2 | Proliferating Macrophage | FOLR2 Macrophage | TGFB1 | TGFbR1_R2 | 6.27E-05 | 0 | TGFB1_TGFBR1_TGFBR2 | TGFB1 - (TGFBR1+TGFBR2) | TGFb | Secreted Signaling | KEGG: hsa04350 | LM |
| 3 | SPP1 Macrophage | FOLR2 Macrophage | TGFB1 | TGFbR1_R2 | 0.00074071 | 0 | TGFB1_TGFBR1_TGFBR2 | TGFB1 - (TGFBR1+TGFBR2) | TGFb | Secreted Signaling | KEGG: hsa04350 | LM |
| 4 | FOLR2 Macrophage | SPP1 Macrophage | TGFB1 | TGFbR1_R2 | 0.000594962 | 0.02 | TGFB1_TGFBR1_TGFBR2 | TGFB1 - (TGFBR1+TGFBR2) | TGFb | Secreted Signaling | KEGG: hsa04350 | LM |
| 5 | SPP1 Macrophage | SPP1 Macrophage | TGFB1 | TGFbR1_R2 | 0.00084997 | 0 | TGFB1_TGFBR1_TGFBR2 | TGFB1 - (TGFBR1+TGFBR2) | TGFb | Secreted Signaling | KEGG: hsa04350 | LM |
| 6 | FOLR2 Macrophage | FOLR2 Macrophage | TGFB2 | TGFbR1_R2 | 8.18E-05 | 0 | TGFB2_TGFBR1_TGFBR2 | TGFB2 - (TGFBR1+TGFBR2) | TGFb | Secreted Signaling | KEGG: hsa04350 | LM |
| 7 | SPP1 Macrophage | FOLR2 Macrophage | TGFB2 | TGFbR1_R2 | 0.00011097 | 0 | TGFB2_TGFBR1_TGFBR2 | TGFB2 - (TGFBR1+TGFBR2) | TGFb | Secreted Signaling | KEGG: hsa04350 | LM |
| 8 | FOLR2 Macrophage | SPP1 Macrophage | TGFB2 | TGFbR1_R2 | 9.38E-05 | 0 | TGFB2_TGFBR1_TGFBR2 | TGFB2 - (TGFBR1+TGFBR2) | TGFb | Secreted Signaling | KEGG: hsa04350 | LM |
| 9 | SPP1 Macrophage | SPP1 Macrophage | TGFB2 | TGFbR1_R2 | 0.000127312 | 0 | TGFB2_TGFBR1_TGFBR2 | TGFB2 - (TGFBR1+TGFBR2) | TGFb | Secreted Signaling | KEGG: hsa04350 | LM |
| 10 | FOLR2 Macrophage | FOLR2 Macrophage | TGFB3 | TGFbR1_R2 | 0.000218922 | 0 | TGFB3_TGFBR1_TGFBR2 | TGFB3 - (TGFBR1+TGFBR2) | TGFb | Secreted Signaling | KEGG: hsa04350 | LM |
| 11 | SPP1 Macrophage | FOLR2 Macrophage | TGFB3 | TGFbR1_R2 | 0.000238577 | 0 | TGFB3_TGFBR1_TGFBR2 | TGFB3 - (TGFBR1+TGFBR2) | TGFb | Secreted Signaling | KEGG: hsa04350 | LM |
| 12 | FOLR2 Macrophage | SPP1 Macrophage | TGFB3 | TGFbR1_R2 | 0.000251174 | 0 | TGFB3_TGFBR1_TGFBR2 | TGFB3 - (TGFBR1+TGFBR2) | TGFb | Secreted Signaling | KEGG: hsa04350 | LM |
| 13 | FOLR2 Macrophage | SPP1 Macrophage | TGFB1 | ACVR1B_TGFbR2 | 0.000425222 | 0 | TGFB1_ACVR1B_TGFBR2 | TGFB1 - (ACVR1B+TGFBR2) | TGFb | Secreted Signaling | PMID: 27449815 | LM |
| 14 | Proliferating Macrophage | SPP1 Macrophage | TGFB1 | ACVR1B_TGFbR2 | 5.14E-05 | 0 | TGFB1_ACVR1B_TGFBR2 | TGFB1 - (ACVR1B+TGFBR2) | TGFb | Secreted Signaling | PMID: 27449815 | LM |
| 15 | SPP1 Macrophage | SPP1 Macrophage | TGFB1 | ACVR1B_TGFbR2 | 0.00060759 | 0 | TGFB1_ACVR1B_TGFBR2 | TGFB1 - (ACVR1B+TGFBR2) | TGFb | Secreted Signaling | PMID: 27449815 | LM |
| 16 | FOLR2 Macrophage | SPP1 Macrophage | TGFB2 | ACVR1B_TGFbR2 | 6.70E-05 | 0 | TGFB2_ACVR1B_TGFBR2 | TGFB2 - (ACVR1B+TGFBR2) | TGFb | Secreted Signaling | PMID: 27449815 | LM |
| 17 | Proliferating Macrophage | SPP1 Macrophage | TGFB2 | ACVR1B_TGFbR2 | 7.47E-06 | 0 | TGFB2_ACVR1B_TGFBR2 | TGFB2 - (ACVR1B+TGFBR2) | TGFb | Secreted Signaling | PMID: 27449815 | LM |
| 18 | SPP1 Macrophage | SPP1 Macrophage | TGFB2 | ACVR1B_TGFbR2 | 9.09E-05 | 0 | TGFB2_ACVR1B_TGFBR2 | TGFB2 - (ACVR1B+TGFBR2) | TGFb | Secreted Signaling | PMID: 27449815 | LM |
| 19 | C9 | SPP1 Macrophage | TGFB3 | ACVR1B_TGFbR2 | 5.40E-05 | 0.01 | TGFB3_ACVR1B_TGFBR2 | TGFB3 - (ACVR1B+TGFBR2) | TGFb | Secreted Signaling | PMID: 27449815 | LM |
| 20 | FOLR2 Macrophage | SPP1 Macrophage | TGFB3 | ACVR1B_TGFbR2 | 0.000179407 | 0 | TGFB3_ACVR1B_TGFBR2 | TGFB3 - (ACVR1B+TGFBR2) | TGFb | Secreted Signaling | PMID: 27449815 | LM |
| 21 | SPP1 Macrophage | SPP1 Macrophage | TGFB3 | ACVR1B_TGFbR2 | 0.000195504 | 0 | TGFB3_ACVR1B_TGFBR2 | TGFB3 - (ACVR1B+TGFBR2) | TGFb | Secreted Signaling | PMID: 27449815 | LM |
| 22 | SPP1 Macrophage | FOLR2 Macrophage | TGFB1 | ACVR1_TGFbR | 0.000197861 | 0 | TGFB1_ACVR1_TGFBR1 | TGFB1 - (ACVR1+TGFBR1) | TGFb | Secreted Signaling | PMID: 29376829 | LM |
| 23 | FOLR2 Macrophage | SPP1 Macrophage | TGFB1 | ACVR1_TGFbR | 0.000172638 | 0 | TGFB1_ACVR1_TGFBR1 | TGFB1 - (ACVR1+TGFBR1) | TGFb | Secreted Signaling | PMID: 29376829 | LM |
| 24 | Proliferating Macrophage | SPP1 Macrophage | TGFB1 | ACVR1_TGFbR | 2.20E-05 | 0.04 | TGFB1_ACVR1_TGFBR1 | TGFB1 - (ACVR1+TGFBR1) | TGFb | Secreted Signaling | PMID: 29376829 | LM |
| 25 | SPP1 Macrophage | SPP1 Macrophage | TGFB1 | ACVR1_TGFbR | 0.000256269 | 0 | TGFB1_ACVR1_TGFBR1 | TGFB1 - (ACVR1+TGFBR1) | TGFb | Secreted Signaling | PMID: 29376829 | LM |
| 26 | FOLR2 Macrophage | FOLR2 Macrophage | TGFB2 | ACVR1_TGFbR | 2.10E-05 | 0.02 | TGFB2_ACVR1_TGFBR1 | TGFB2 - (ACVR1+TGFBR1) | TGFb | Secreted Signaling | PMID: 29376829 | LM |
| 27 | SPP1 Macrophage | FOLR2 Macrophage | TGFB2 | ACVR1_TGFbR | 2.96E-05 | 0 | TGFB2_ACVR1_TGFBR1 | TGFB2 - (ACVR1+TGFBR1) | TGFb | Secreted Signaling | PMID: 29376829 | LM |
| 28 | FOLR2 Macrophage | SPP1 Macrophage | TGFB2 | ACVR1_TGFbR | 2.72E-05 | 0 | TGFB2_ACVR1_TGFBR1 | TGFB2 - (ACVR1+TGFBR1) | TGFb | Secreted Signaling | PMID: 29376829 | LM |
| 29 | SPP1 Macrophage | SPP1 Macrophage | TGFB2 | ACVR1_TGFbR | 3.83E-05 | 0 | TGFB2_ACVR1_TGFBR1 | TGFB2 - (ACVR1+TGFBR1) | TGFb | Secreted Signaling | PMID: 29376829 | LM |
| 30 | FOLR2 Macrophage | FOLR2 Macrophage | TGFB3 | ACVR1_TGFbR | 5.62E-05 | 0 | TGFB3_ACVR1_TGFBR1 | TGFB3 - (ACVR1+TGFBR1) | TGFb | Secreted Signaling | PMID: 29376829 | LM |
| 31 | SPP1 Macrophage | FOLR2 Macrophage | TGFB3 | ACVR1_TGFbR | 6.36E-05 | 0 | TGFB3_ACVR1_TGFBR1 | TGFB3 - (ACVR1+TGFBR1) | TGFb | Secreted Signaling | PMID: 29376829 | LM |
| 32 | FOLR2 Macrophage | SPP1 Macrophage | TGFB3 | ACVR1_TGFbR | 7.28E-05 | 0 | TGFB3_ACVR1_TGFBR1 | TGFB3 - (ACVR1+TGFBR1) | TGFb | Secreted Signaling | PMID: 29376829 | LM |
| 33 | SPP1 Macrophage | SPP1 Macrophage | TGFB3 | ACVR1_TGFbR | 8.24E-05 | 0 | TGFB3_ACVR1_TGFBR1 | TGFB3 - (ACVR1+TGFBR1) | TGFb | Secreted Signaling | PMID: 29376829 | LM |
| 34 | C9 | C9 | BMP2 | BMPR1B_ACVR2A | 1.23E-06 | 0 | BMP2_BMPR1B_ACVR2A | BMP2 - (BMPR1B+ACVR2A) | BMP | Secreted Signaling | KEGG: hsa04350; PMID:26893264 | LM |
| 35 | C9 | Proliferating Macrophage | BMP2 | BMPR1B_ACVR2A | 2.51E-07 | 0 | BMP2_BMPR1B_ACVR2A | BMP2 - (BMPR1B+ACVR2A) | BMP | Secreted Signaling | KEGG: hsa04350; PMID:26893264 | LM |
| 36 | C9 | SPP1 Macrophage | BMP2 | BMPR1B_ACVR2A | 5.08E-06 | 0 | BMP2_BMPR1B_ACVR2A | BMP2 - (BMPR1B+ACVR2A) | BMP | Secreted Signaling | KEGG: hsa04350; PMID:26893264 | LM |
| 37 | SPP1 Macrophage | SPP1 Macrophage | BMP2 | BMPR1B_ACVR2A | 6.00E-06 | 0 | BMP2_BMPR1B_ACVR2A | BMP2 - (BMPR1B+ACVR2A) | BMP | Secreted Signaling | KEGG: hsa04350; PMID:26893264 | LM |
| 38 | C9 | C9 | BMP2 | BMPR1B_ACVR2B | 1.06E-06 | 0 | BMP2_BMPR1B_ACVR2B | BMP2 - (BMPR1B+ACVR2B) | BMP | Secreted Signaling | KEGG: hsa04350; PMID:26893264 | LM |
| 39 | SPP1 Macrophage | C9 | BMP2 | BMPR1B_ACVR2B | 1.25E-06 | 0 | BMP2_BMPR1B_ACVR2B | BMP2 - (BMPR1B+ACVR2B) | BMP | Secreted Signaling | KEGG: hsa04350; PMID:26893264 | LM |
| 40 | C9 | FOLR2 Macrophage | BMP2 | BMPR1B_ACVR2B | 1.69E-06 | 0 | BMP2_BMPR1B_ACVR2B | BMP2 - (BMPR1B+ACVR2B) | BMP | Secreted Signaling | KEGG: hsa04350; PMID:26893264 | LM |
| 41 | C9 | Proliferating Macrophage | BMP2 | BMPR1B_ACVR2B | 2.00E-07 | 0 | BMP2_BMPR1B_ACVR2B | BMP2 - (BMPR1B+ACVR2B) | BMP | Secreted Signaling | KEGG: hsa04350; PMID:26893264 | LM |
| 42 | C9 | SPP1 Macrophage | BMP2 | BMPR1B_ACVR2B | 3.56E-06 | 0 | BMP2_BMPR1B_ACVR2B | BMP2 - (BMPR1B+ACVR2B) | BMP | Secreted Signaling | KEGG: hsa04350; PMID:26893264 | LM |
| 43 | SPP1 Macrophage | SPP1 Macrophage | BMP2 | BMPR1B_ACVR2B | 4.20E-06 | 0 | BMP2_BMPR1B_ACVR2B | BMP2 - (BMPR1B+ACVR2B) | BMP | Secreted Signaling | KEGG: hsa04350; PMID:26893264 | LM |
| 44 | C9 | C9 | BMP2 | BMPR1B_BMPR2 | 1.91E-06 | 0 | BMP2_BMPR1B_BMPR2 | BMP2 - (BMPR1B+BMPR2) | BMP | Secreted Signaling | KEGG: hsa04350; PMID:26893264 | LM |
| 45 | FOLR2 Macrophage | C9 | BMP2 | BMPR1B_BMPR2 | 1.24E-06 | 0.01 | BMP2_BMPR1B_BMPR2 | BMP2 - (BMPR1B+BMPR2) | BMP | Secreted Signaling | KEGG: hsa04350; PMID:26893264 | LM |
| 46 | SPP1 Macrophage | C9 | BMP2 | BMPR1B_BMPR2 | 2.26E-06 | 0 | BMP2_BMPR1B_BMPR2 | BMP2 - (BMPR1B+BMPR2) | BMP | Secreted Signaling | KEGG: hsa04350; PMID:26893264 | LM |
| 47 | C9 | FOLR2 Macrophage | BMP2 | BMPR1B_BMPR2 | 2.93E-06 | 0 | BMP2_BMPR1B_BMPR2 | BMP2 - (BMPR1B+BMPR2) | BMP | Secreted Signaling | KEGG: hsa04350; PMID:26893264 | LM |
| 48 | SPP1 Macrophage | FOLR2 Macrophage | BMP2 | BMPR1B_BMPR2 | 3.46E-06 | 0.01 | BMP2_BMPR1B_BMPR2 | BMP2 - (BMPR1B+BMPR2) | BMP | Secreted Signaling | KEGG: hsa04350; PMID:26893264 | LM |
| 49 | C9 | Proliferating Macrophage | BMP2 | BMPR1B_BMPR2 | 2.29E-07 | 0 | BMP2_BMPR1B_BMPR2 | BMP2 - (BMPR1B+BMPR2) | BMP | Secreted Signaling | KEGG: hsa04350; PMID:26893264 | LM |
| 50 | C9 | SPP1 Macrophage | BMP2 | BMPR1B_BMPR2 | 3.63E-06 | 0 | BMP2_BMPR1B_BMPR2 | BMP2 - (BMPR1B+BMPR2) | BMP | Secreted Signaling | KEGG: hsa04350; PMID:26893264 | LM |
| 51 | C9 | C9 | BMP4 | BMPR1B_ACVR2A | 9.75E-07 | 0 | BMP4_BMPR1B_ACVR2A | BMP4 - (BMPR1B+ACVR2A) | BMP | Secreted Signaling | KEGG: hsa04350; PMID:26893264 | LM |
| 52 | C9 | Proliferating Macrophage | BMP4 | BMPR1B_ACVR2A | 2.00E-07 | 0 | BMP4_BMPR1B_ACVR2A | BMP4 - (BMPR1B+ACVR2A) | BMP | Secreted Signaling | KEGG: hsa04350; PMID:26893264 | LM |
| 53 | C9 | SPP1 Macrophage | BMP4 | BMPR1B_ACVR2A | 4.04E-06 | 0 | BMP4_BMPR1B_ACVR2A | BMP4 - (BMPR1B+ACVR2A) | BMP | Secreted Signaling | KEGG: hsa04350; PMID:26893264 | LM |
| 54 | FOLR2 Macrophage | SPP1 Macrophage | BMP4 | BMPR1B_ACVR2A | 3.91E-06 | 0 | BMP4_BMPR1B_ACVR2A | BMP4 - (BMPR1B+ACVR2A) | BMP | Secreted Signaling | KEGG: hsa04350; PMID:26893264 | LM |
| 55 | SPP1 Macrophage | SPP1 Macrophage | BMP4 | BMPR1B_ACVR2A | 4.95E-06 | 0 | BMP4_BMPR1B_ACVR2A | BMP4 - (BMPR1B+ACVR2A) | BMP | Secreted Signaling | KEGG: hsa04350; PMID:26893264 | LM |
| 56 | C9 | C9 | BMP4 | BMPR1B_ACVR2B | 8.45E-07 | 0 | BMP4_BMPR1B_ACVR2B | BMP4 - (BMPR1B+ACVR2B) | BMP | Secreted Signaling | KEGG: hsa04350; PMID:26893264 | LM |
| 57 | C9 | FOLR2 Macrophage | BMP4 | BMPR1B_ACVR2B | 1.34E-06 | 0 | BMP4_BMPR1B_ACVR2B | BMP4 - (BMPR1B+ACVR2B) | BMP | Secreted Signaling | KEGG: hsa04350; PMID:26893264 | LM |
| 58 | C9 | Proliferating Macrophage | BMP4 | BMPR1B_ACVR2B | 1.60E-07 | 0 | BMP4_BMPR1B_ACVR2B | BMP4 - (BMPR1B+ACVR2B) | BMP | Secreted Signaling | KEGG: hsa04350; PMID:26893264 | LM |
| 59 | C9 | SPP1 Macrophage | BMP4 | BMPR1B_ACVR2B | 2.83E-06 | 0 | BMP4_BMPR1B_ACVR2B | BMP4 - (BMPR1B+ACVR2B) | BMP | Secreted Signaling | KEGG: hsa04350; PMID:26893264 | LM |
| 60 | FOLR2 Macrophage | SPP1 Macrophage | BMP4 | BMPR1B_ACVR2B | 2.74E-06 | 0 | BMP4_BMPR1B_ACVR2B | BMP4 - (BMPR1B+ACVR2B) | BMP | Secreted Signaling | KEGG: hsa04350; PMID:26893264 | LM |
| 61 | SPP1 Macrophage | SPP1 Macrophage | BMP4 | BMPR1B_ACVR2B | 3.47E-06 | 0 | BMP4_BMPR1B_ACVR2B | BMP4 - (BMPR1B+ACVR2B) | BMP | Secreted Signaling | KEGG: hsa04350; PMID:26893264 | LM |
| 62 | C9 | C9 | BMP4 | BMPR1B_BMPR2 | 1.52E-06 | 0 | BMP4_BMPR1B_BMPR2 | BMP4 - (BMPR1B+BMPR2) | BMP | Secreted Signaling | KEGG: hsa04350; PMID:26893264 | LM |
| 63 | FOLR2 Macrophage | C9 | BMP4 | BMPR1B_BMPR2 | 1.47E-06 | 0 | BMP4_BMPR1B_BMPR2 | BMP4 - (BMPR1B+BMPR2) | BMP | Secreted Signaling | KEGG: hsa04350; PMID:26893264 | LM |
| 64 | SPP1 Macrophage | C9 | BMP4 | BMPR1B_BMPR2 | 1.86E-06 | 0 | BMP4_BMPR1B_BMPR2 | BMP4 - (BMPR1B+BMPR2) | BMP | Secreted Signaling | KEGG: hsa04350; PMID:26893264 | LM |
| 65 | C9 | FOLR2 Macrophage | BMP4 | BMPR1B_BMPR2 | 2.33E-06 | 0 | BMP4_BMPR1B_BMPR2 | BMP4 - (BMPR1B+BMPR2) | BMP | Secreted Signaling | KEGG: hsa04350; PMID:26893264 | LM |
| 66 | C9 | Proliferating Macrophage | BMP4 | BMPR1B_BMPR2 | 1.82E-07 | 0 | BMP4_BMPR1B_BMPR2 | BMP4 - (BMPR1B+BMPR2) | BMP | Secreted Signaling | KEGG: hsa04350; PMID:26893264 | LM |
| 67 | C9 | SPP1 Macrophage | BMP4 | BMPR1B_BMPR2 | 2.89E-06 | 0 | BMP4_BMPR1B_BMPR2 | BMP4 - (BMPR1B+BMPR2) | BMP | Secreted Signaling | KEGG: hsa04350; PMID:26893264 | LM |
| 68 | C9 | C9 | GDF15 | TGFBR2 | 2.78E-05 | 0 | GDF15_TGFBR2 | GDF15 - TGFBR2 | GDF | Secreted Signaling | KEGG: hsa04350 | LM |
| 69 | C9 | FOLR2 Macrophage | GDF15 | TGFBR2 | 8.65E-05 | 0 | GDF15_TGFBR2 | GDF15 - TGFBR2 | GDF | Secreted Signaling | KEGG: hsa04350 | LM |
| 70 | C9 | Proliferating Macrophage | GDF15 | TGFBR2 | 6.89E-06 | 0 | GDF15_TGFBR2 | GDF15 - TGFBR2 | GDF | Secreted Signaling | KEGG: hsa04350 | LM |
| 71 | C9 | SPP1 Macrophage | GDF15 | TGFBR2 | 9.37E-05 | 0 | GDF15_TGFBR2 | GDF15 - TGFBR2 | GDF | Secreted Signaling | KEGG: hsa04350 | LM |
| 72 | SPP1 Macrophage | C9 | INHBA | ACVR1B_ACVR2A | 2.63E-05 | 0 | INHBA_ACVR1B_ACVR2A | INHBA - (ACVR1B+ACVR2A) | ACTIVIN | Secreted Signaling | KEGG: hsa04350 | LM |
| 73 | SPP1 Macrophage | Proliferating Macrophage | INHBA | ACVR1B_ACVR2A | 6.33E-06 | 0 | INHBA_ACVR1B_ACVR2A | INHBA - (ACVR1B+ACVR2A) | ACTIVIN | Secreted Signaling | KEGG: hsa04350 | LM |
| 74 | C9 | SPP1 Macrophage | INHBA | ACVR1B_ACVR2A | 1.82E-05 | 0.01 | INHBA_ACVR1B_ACVR2A | INHBA - (ACVR1B+ACVR2A) | ACTIVIN | Secreted Signaling | KEGG: hsa04350 | LM |
| 75 | SPP1 Macrophage | SPP1 Macrophage | INHBA | ACVR1B_ACVR2A | 0.00017089 | 0 | INHBA_ACVR1B_ACVR2A | INHBA - (ACVR1B+ACVR2A) | ACTIVIN | Secreted Signaling | KEGG: hsa04350 | LM |
| 76 | SPP1 Macrophage | C9 | INHBA | ACVR1B_ACVR2B | 2.28E-05 | 0 | INHBA_ACVR1B_ACVR2B | INHBA - (ACVR1B+ACVR2B) | ACTIVIN | Secreted Signaling | KEGG: hsa04351 | LM |
| 77 | SPP1 Macrophage | Proliferating Macrophage | INHBA | ACVR1B_ACVR2B | 5.05E-06 | 0 | INHBA_ACVR1B_ACVR2B | INHBA - (ACVR1B+ACVR2B) | ACTIVIN | Secreted Signaling | KEGG: hsa04351 | LM |
| 78 | SPP1 Macrophage | SPP1 Macrophage | INHBA | ACVR1B_ACVR2B | 0.000119829 | 0 | INHBA_ACVR1B_ACVR2B | INHBA - (ACVR1B+ACVR2B) | ACTIVIN | Secreted Signaling | KEGG: hsa04351 | LM |
| 79 | C9 | C9 | EGF | EGFR | 1.86E-06 | 0 | EGF_EGFR | EGF - EGFR | EGF | Secreted Signaling | KEGG: hsa04012 | LM |
| 80 | FOLR2 Macrophage | C9 | EGF | EGFR | 1.06E-06 | 0 | EGF_EGFR | EGF - EGFR | EGF | Secreted Signaling | KEGG: hsa04012 | LM |
| 81 | Proliferating Macrophage | C9 | EGF | EGFR | 1.48E-07 | 0 | EGF_EGFR | EGF - EGFR | EGF | Secreted Signaling | KEGG: hsa04012 | LM |
| 82 | SPP1 Macrophage | C9 | EGF | EGFR | 1.41E-06 | 0 | EGF_EGFR | EGF - EGFR | EGF | Secreted Signaling | KEGG: hsa04012 | LM |
| 83 | C9 | FOLR2 Macrophage | EGF | EGFR | 1.22E-06 | 0 | EGF_EGFR | EGF - EGFR | EGF | Secreted Signaling | KEGG: hsa04012 | LM |
| 84 | C9 | Proliferating Macrophage | EGF | EGFR | 1.70E-07 | 0 | EGF_EGFR | EGF - EGFR | EGF | Secreted Signaling | KEGG: hsa04012 | LM |
| 85 | Proliferating Macrophage | Proliferating Macrophage | EGF | EGFR | 1.36E-08 | 0 | EGF_EGFR | EGF - EGFR | EGF | Secreted Signaling | KEGG: hsa04012 | LM |
| 86 | C9 | SPP1 Macrophage | EGF | EGFR | 1.61E-06 | 0 | EGF_EGFR | EGF - EGFR | EGF | Secreted Signaling | KEGG: hsa04012 | LM |
| 87 | C9 | C9 | EGF | EGFR_ERBB2 | 2.54E-06 | 0 | EGF_EGFR_ERBB2 | EGF - (EGFR+ERBB2) | EGF | Secreted Signaling | KEGG: hsa04012 | LM |
| 88 | FOLR2 Macrophage | C9 | EGF | EGFR_ERBB2 | 1.45E-06 | 0 | EGF_EGFR_ERBB2 | EGF - (EGFR+ERBB2) | EGF | Secreted Signaling | KEGG: hsa04012 | LM |
| 89 | Proliferating Macrophage | C9 | EGF | EGFR_ERBB2 | 2.02E-07 | 0 | EGF_EGFR_ERBB2 | EGF - (EGFR+ERBB2) | EGF | Secreted Signaling | KEGG: hsa04012 | LM |
| 90 | SPP1 Macrophage | C9 | EGF | EGFR_ERBB2 | 1.93E-06 | 0 | EGF_EGFR_ERBB2 | EGF - (EGFR+ERBB2) | EGF | Secreted Signaling | KEGG: hsa04012 | LM |
| 91 | C9 | FOLR2 Macrophage | EGF | EGFR_ERBB2 | 1.82E-06 | 0 | EGF_EGFR_ERBB2 | EGF - (EGFR+ERBB2) | EGF | Secreted Signaling | KEGG: hsa04012 | LM |
| 92 | C9 | Proliferating Macrophage | EGF | EGFR_ERBB2 | 2.59E-07 | 0 | EGF_EGFR_ERBB2 | EGF - (EGFR+ERBB2) | EGF | Secreted Signaling | KEGG: hsa04012 | LM |
| 93 | Proliferating Macrophage | Proliferating Macrophage | EGF | EGFR_ERBB2 | 2.06E-08 | 0 | EGF_EGFR_ERBB2 | EGF - (EGFR+ERBB2) | EGF | Secreted Signaling | KEGG: hsa04012 | LM |
| 94 | SPP1 Macrophage | Proliferating Macrophage | EGF | EGFR_ERBB2 | 1.97E-07 | 0.04 | EGF_EGFR_ERBB2 | EGF - (EGFR+ERBB2) | EGF | Secreted Signaling | KEGG: hsa04012 | LM |
| 95 | C9 | SPP1 Macrophage | EGF | EGFR_ERBB2 | 2.40E-06 | 0 | EGF_EGFR_ERBB2 | EGF - (EGFR+ERBB2) | EGF | Secreted Signaling | KEGG: hsa04012 | LM |
| 96 | C9 | C9 | TGFA | EGFR | 5.05E-06 | 0 | TGFA_EGFR | TGFA - EGFR | EGF | Secreted Signaling | KEGG: hsa04012 | LM |
| 97 | FOLR2 Macrophage | C9 | TGFA | EGFR | 5.53E-06 | 0 | TGFA_EGFR | TGFA - EGFR | EGF | Secreted Signaling | KEGG: hsa04012 | LM |
| 98 | Proliferating Macrophage | C9 | TGFA | EGFR | 7.03E-07 | 0 | TGFA_EGFR | TGFA - EGFR | EGF | Secreted Signaling | KEGG: hsa04012 | LM |
| 99 | SPP1 Macrophage | C9 | TGFA | EGFR | 6.98E-06 | 0 | TGFA_EGFR | TGFA - EGFR | EGF | Secreted Signaling | KEGG: hsa04012 | LM |
| 100 | C9 | FOLR2 Macrophage | TGFA | EGFR | 3.31E-06 | 0 | TGFA_EGFR | TGFA - EGFR | EGF | Secreted Signaling | KEGG: hsa04012 | LM |
| 101 | C9 | Proliferating Macrophage | TGFA | EGFR | 4.63E-07 | 0 | TGFA_EGFR | TGFA - EGFR | EGF | Secreted Signaling | KEGG: hsa04012 | LM |
| 102 | C9 | SPP1 Macrophage | TGFA | EGFR | 4.39E-06 | 0 | TGFA_EGFR | TGFA - EGFR | EGF | Secreted Signaling | KEGG: hsa04012 | LM |
| 103 | C9 | C9 | TGFA | EGFR_ERBB2 | 6.90E-06 | 0 | TGFA_EGFR_ERBB2 | TGFA - (EGFR+ERBB2) | EGF | Secreted Signaling | KEGG: hsa04012 | LM |
| 104 | FOLR2 Macrophage | C9 | TGFA | EGFR_ERBB2 | 7.56E-06 | 0 | TGFA_EGFR_ERBB2 | TGFA - (EGFR+ERBB2) | EGF | Secreted Signaling | KEGG: hsa04012 | LM |
| 105 | Proliferating Macrophage | C9 | TGFA | EGFR_ERBB2 | 9.62E-07 | 0 | TGFA_EGFR_ERBB2 | TGFA - (EGFR+ERBB2) | EGF | Secreted Signaling | KEGG: hsa04012 | LM |
| 106 | SPP1 Macrophage | C9 | TGFA | EGFR_ERBB2 | 9.55E-06 | 0 | TGFA_EGFR_ERBB2 | TGFA - (EGFR+ERBB2) | EGF | Secreted Signaling | KEGG: hsa04012 | LM |
| 107 | C9 | FOLR2 Macrophage | TGFA | EGFR_ERBB2 | 4.96E-06 | 0 | TGFA_EGFR_ERBB2 | TGFA - (EGFR+ERBB2) | EGF | Secreted Signaling | KEGG: hsa04012 | LM |
| 108 | C9 | Proliferating Macrophage | TGFA | EGFR_ERBB2 | 7.04E-07 | 0 | TGFA_EGFR_ERBB2 | TGFA - (EGFR+ERBB2) | EGF | Secreted Signaling | KEGG: hsa04012 | LM |
| 109 | C9 | SPP1 Macrophage | TGFA | EGFR_ERBB2 | 6.53E-06 | 0 | TGFA_EGFR_ERBB2 | TGFA - (EGFR+ERBB2) | EGF | Secreted Signaling | KEGG: hsa04012 | LM |
| 110 | C9 | C9 | AREG | EGFR | 5.63E-05 | 0 | AREG_EGFR | AREG - EGFR | EGF | Secreted Signaling | KEGG: hsa04012 | LM |
| 111 | Proliferating Macrophage | C9 | AREG | EGFR | 4.40E-06 | 0 | AREG_EGFR | AREG - EGFR | EGF | Secreted Signaling | KEGG: hsa04012 | LM |
| 112 | SPP1 Macrophage | C9 | AREG | EGFR | 3.54E-05 | 0 | AREG_EGFR | AREG - EGFR | EGF | Secreted Signaling | KEGG: hsa04012 | LM |
| 113 | C9 | FOLR2 Macrophage | AREG | EGFR | 3.70E-05 | 0 | AREG_EGFR | AREG - EGFR | EGF | Secreted Signaling | KEGG: hsa04012 | LM |
| 114 | C9 | Proliferating Macrophage | AREG | EGFR | 5.18E-06 | 0 | AREG_EGFR | AREG - EGFR | EGF | Secreted Signaling | KEGG: hsa04012 | LM |
| 115 | C9 | SPP1 Macrophage | AREG | EGFR | 4.91E-05 | 0 | AREG_EGFR | AREG - EGFR | EGF | Secreted Signaling | KEGG: hsa04012 | LM |
| 116 | C9 | C9 | AREG | EGFR_ERBB2 | 7.70E-05 | 0 | AREG_EGFR_ERBB2 | AREG - (EGFR+ERBB2) | EGF | Secreted Signaling | KEGG: hsa04012 | LM |
| 117 | Proliferating Macrophage | C9 | AREG | EGFR_ERBB2 | 6.02E-06 | 0 | AREG_EGFR_ERBB2 | AREG - (EGFR+ERBB2) | EGF | Secreted Signaling | KEGG: hsa04012 | LM |
| 118 | SPP1 Macrophage | C9 | AREG | EGFR_ERBB2 | 4.85E-05 | 0 | AREG_EGFR_ERBB2 | AREG - (EGFR+ERBB2) | EGF | Secreted Signaling | KEGG: hsa04012 | LM |
| 119 | C9 | FOLR2 Macrophage | AREG | EGFR_ERBB2 | 5.54E-05 | 0 | AREG_EGFR_ERBB2 | AREG - (EGFR+ERBB2) | EGF | Secreted Signaling | KEGG: hsa04012 | LM |
| 120 | C9 | Proliferating Macrophage | AREG | EGFR_ERBB2 | 7.87E-06 | 0 | AREG_EGFR_ERBB2 | AREG - (EGFR+ERBB2) | EGF | Secreted Signaling | KEGG: hsa04012 | LM |
| 121 | C9 | SPP1 Macrophage | AREG | EGFR_ERBB2 | 7.30E-05 | 0 | AREG_EGFR_ERBB2 | AREG - (EGFR+ERBB2) | EGF | Secreted Signaling | KEGG: hsa04012 | LM |
| 122 | C9 | C9 | HBEGF | EGFR | 2.82E-05 | 0 | HBEGF_EGFR | HBEGF - EGFR | EGF | Secreted Signaling | KEGG: hsa04012 | LM |
| 123 | FOLR2 Macrophage | C9 | HBEGF | EGFR | 4.61E-05 | 0 | HBEGF_EGFR | HBEGF - EGFR | EGF | Secreted Signaling | KEGG: hsa04012 | LM |
| 124 | Proliferating Macrophage | C9 | HBEGF | EGFR | 1.02E-05 | 0 | HBEGF_EGFR | HBEGF - EGFR | EGF | Secreted Signaling | KEGG: hsa04012 | LM |
| 125 | SPP1 Macrophage | C9 | HBEGF | EGFR | 0.000125836 | 0 | HBEGF_EGFR | HBEGF - EGFR | EGF | Secreted Signaling | KEGG: hsa04012 | LM |
| 126 | SPP1 Macrophage | FOLR2 Macrophage | HBEGF | EGFR | 8.27E-05 | 0 | HBEGF_EGFR | HBEGF - EGFR | EGF | Secreted Signaling | KEGG: hsa04012 | LM |
| 127 | SPP1 Macrophage | Proliferating Macrophage | HBEGF | EGFR | 1.16E-05 | 0 | HBEGF_EGFR | HBEGF - EGFR | EGF | Secreted Signaling | KEGG: hsa04012 | LM |
| 128 | SPP1 Macrophage | SPP1 Macrophage | HBEGF | EGFR | 0.000109564 | 0 | HBEGF_EGFR | HBEGF - EGFR | EGF | Secreted Signaling | KEGG: hsa04012 | LM |
| 129 | C9 | C9 | HBEGF | EGFR_ERBB2 | 3.85E-05 | 0 | HBEGF_EGFR_ERBB2 | HBEGF - (EGFR+ERBB2) | EGF | Secreted Signaling | KEGG: hsa04012 | LM |
| 130 | FOLR2 Macrophage | C9 | HBEGF | EGFR_ERBB2 | 6.31E-05 | 0 | HBEGF_EGFR_ERBB2 | HBEGF - (EGFR+ERBB2) | EGF | Secreted Signaling | KEGG: hsa04012 | LM |
| 131 | Proliferating Macrophage | C9 | HBEGF | EGFR_ERBB2 | 1.39E-05 | 0 | HBEGF_EGFR_ERBB2 | HBEGF - (EGFR+ERBB2) | EGF | Secreted Signaling | KEGG: hsa04012 | LM |
| 132 | SPP1 Macrophage | C9 | HBEGF | EGFR_ERBB2 | 0.000172013 | 0 | HBEGF_EGFR_ERBB2 | HBEGF - (EGFR+ERBB2) | EGF | Secreted Signaling | KEGG: hsa04012 | LM |
| 133 | SPP1 Macrophage | FOLR2 Macrophage | HBEGF | EGFR_ERBB2 | 0.000123699 | 0 | HBEGF_EGFR_ERBB2 | HBEGF - (EGFR+ERBB2) | EGF | Secreted Signaling | KEGG: hsa04012 | LM |
| 134 | SPP1 Macrophage | Proliferating Macrophage | HBEGF | EGFR_ERBB2 | 1.76E-05 | 0 | HBEGF_EGFR_ERBB2 | HBEGF - (EGFR+ERBB2) | EGF | Secreted Signaling | KEGG: hsa04012 | LM |
| 135 | SPP1 Macrophage | SPP1 Macrophage | HBEGF | EGFR_ERBB2 | 0.000162911 | 0 | HBEGF_EGFR_ERBB2 | HBEGF - (EGFR+ERBB2) | EGF | Secreted Signaling | KEGG: hsa04012 | LM |
| 136 | C9 | C9 | HBEGF | ERBB4 | 1.22E-05 | 0 | HBEGF_ERBB4 | HBEGF - ERBB4 | EGF | Secreted Signaling | KEGG: hsa04012 | LM |
| 137 | FOLR2 Macrophage | C9 | HBEGF | ERBB4 | 1.99E-05 | 0 | HBEGF_ERBB4 | HBEGF - ERBB4 | EGF | Secreted Signaling | KEGG: hsa04012 | LM |
| 138 | Proliferating Macrophage | C9 | HBEGF | ERBB4 | 4.41E-06 | 0 | HBEGF_ERBB4 | HBEGF - ERBB4 | EGF | Secreted Signaling | KEGG: hsa04012 | LM |
| 139 | SPP1 Macrophage | C9 | HBEGF | ERBB4 | 5.44E-05 | 0 | HBEGF_ERBB4 | HBEGF - ERBB4 | EGF | Secreted Signaling | KEGG: hsa04012 | LM |
| 140 | SPP1 Macrophage | FOLR2 Macrophage | HBEGF | ERBB4 | 2.16E-05 | 0.02 | HBEGF_ERBB4 | HBEGF - ERBB4 | EGF | Secreted Signaling | KEGG: hsa04012 | LM |
| 141 | SPP1 Macrophage | Proliferating Macrophage | HBEGF | ERBB4 | 3.09E-06 | 0 | HBEGF_ERBB4 | HBEGF - ERBB4 | EGF | Secreted Signaling | KEGG: hsa04012 | LM |
| 142 | SPP1 Macrophage | SPP1 Macrophage | HBEGF | ERBB4 | 3.04E-05 | 0 | HBEGF_ERBB4 | HBEGF - ERBB4 | EGF | Secreted Signaling | KEGG: hsa04012 | LM |
| 143 | C9 | C9 | HBEGF | ERBB2_ERBB4 | 2.53E-05 | 0 | HBEGF_ERBB2_ERBB4 | HBEGF - (ERBB2+ERBB4) | EGF | Secreted Signaling | KEGG: hsa04012 | LM |
| 144 | FOLR2 Macrophage | C9 | HBEGF | ERBB2_ERBB4 | 4.15E-05 | 0 | HBEGF_ERBB2_ERBB4 | HBEGF - (ERBB2+ERBB4) | EGF | Secreted Signaling | KEGG: hsa04012 | LM |
| 145 | Proliferating Macrophage | C9 | HBEGF | ERBB2_ERBB4 | 9.17E-06 | 0 | HBEGF_ERBB2_ERBB4 | HBEGF - (ERBB2+ERBB4) | EGF | Secreted Signaling | KEGG: hsa04012 | LM |
| 146 | SPP1 Macrophage | C9 | HBEGF | ERBB2_ERBB4 | 0.000113127 | 0 | HBEGF_ERBB2_ERBB4 | HBEGF - (ERBB2+ERBB4) | EGF | Secreted Signaling | KEGG: hsa04012 | LM |
| 147 | SPP1 Macrophage | FOLR2 Macrophage | HBEGF | ERBB2_ERBB4 | 6.32E-05 | 0 | HBEGF_ERBB2_ERBB4 | HBEGF - (ERBB2+ERBB4) | EGF | Secreted Signaling | KEGG: hsa04012 | LM |
| 148 | SPP1 Macrophage | Proliferating Macrophage | HBEGF | ERBB2_ERBB4 | 9.08E-06 | 0 | HBEGF_ERBB2_ERBB4 | HBEGF - (ERBB2+ERBB4) | EGF | Secreted Signaling | KEGG: hsa04012 | LM |
| 149 | SPP1 Macrophage | SPP1 Macrophage | HBEGF | ERBB2_ERBB4 | 8.59E-05 | 0 | HBEGF_ERBB2_ERBB4 | HBEGF - (ERBB2+ERBB4) | EGF | Secreted Signaling | KEGG: hsa04012 | LM |
| 150 | C9 | C9 | EREG | EGFR | 1.03E-05 | 0 | EREG_EGFR | EREG - EGFR | EGF | Secreted Signaling | KEGG: hsa04012 | LM |
| 151 | Proliferating Macrophage | C9 | EREG | EGFR | 1.84E-06 | 0 | EREG_EGFR | EREG - EGFR | EGF | Secreted Signaling | KEGG: hsa04012 | LM |
| 152 | SPP1 Macrophage | C9 | EREG | EGFR | 2.78E-05 | 0 | EREG_EGFR | EREG - EGFR | EGF | Secreted Signaling | KEGG: hsa04012 | LM |
| 153 | C9 | FOLR2 Macrophage | EREG | EGFR | 6.75E-06 | 0 | EREG_EGFR | EREG - EGFR | EGF | Secreted Signaling | KEGG: hsa04012 | LM |
| 154 | SPP1 Macrophage | FOLR2 Macrophage | EREG | EGFR | 1.82E-05 | 0 | EREG_EGFR | EREG - EGFR | EGF | Secreted Signaling | KEGG: hsa04012 | LM |
| 155 | C9 | Proliferating Macrophage | EREG | EGFR | 9.44E-07 | 0 | EREG_EGFR | EREG - EGFR | EGF | Secreted Signaling | KEGG: hsa04012 | LM |
| 156 | Proliferating Macrophage | Proliferating Macrophage | EREG | EGFR | 1.69E-07 | 0.04 | EREG_EGFR | EREG - EGFR | EGF | Secreted Signaling | KEGG: hsa04012 | LM |
| 157 | SPP1 Macrophage | Proliferating Macrophage | EREG | EGFR | 2.55E-06 | 0 | EREG_EGFR | EREG - EGFR | EGF | Secreted Signaling | KEGG: hsa04012 | LM |
| 158 | C9 | SPP1 Macrophage | EREG | EGFR | 8.94E-06 | 0 | EREG_EGFR | EREG - EGFR | EGF | Secreted Signaling | KEGG: hsa04012 | LM |
| 159 | SPP1 Macrophage | SPP1 Macrophage | EREG | EGFR | 2.41E-05 | 0 | EREG_EGFR | EREG - EGFR | EGF | Secreted Signaling | KEGG: hsa04012 | LM |
| 160 | C9 | C9 | EREG | EGFR_ERBB2 | 1.41E-05 | 0 | EREG_EGFR_ERBB2 | EREG - (EGFR+ERBB2) | EGF | Secreted Signaling | KEGG: hsa04012 | LM |
| 161 | Proliferating Macrophage | C9 | EREG | EGFR_ERBB2 | 2.52E-06 | 0 | EREG_EGFR_ERBB2 | EREG - (EGFR+ERBB2) | EGF | Secreted Signaling | KEGG: hsa04012 | LM |
| 162 | SPP1 Macrophage | C9 | EREG | EGFR_ERBB2 | 3.80E-05 | 0 | EREG_EGFR_ERBB2 | EREG - (EGFR+ERBB2) | EGF | Secreted Signaling | KEGG: hsa04012 | LM |
| 163 | C9 | FOLR2 Macrophage | EREG | EGFR_ERBB2 | 1.01E-05 | 0 | EREG_EGFR_ERBB2 | EREG - (EGFR+ERBB2) | EGF | Secreted Signaling | KEGG: hsa04012 | LM |
| 164 | SPP1 Macrophage | FOLR2 Macrophage | EREG | EGFR_ERBB2 | 2.73E-05 | 0 | EREG_EGFR_ERBB2 | EREG - (EGFR+ERBB2) | EGF | Secreted Signaling | KEGG: hsa04012 | LM |
| 165 | C9 | Proliferating Macrophage | EREG | EGFR_ERBB2 | 1.43E-06 | 0 | EREG_EGFR_ERBB2 | EREG - (EGFR+ERBB2) | EGF | Secreted Signaling | KEGG: hsa04012 | LM |
| 166 | Proliferating Macrophage | Proliferating Macrophage | EREG | EGFR_ERBB2 | 2.57E-07 | 0.04 | EREG_EGFR_ERBB2 | EREG - (EGFR+ERBB2) | EGF | Secreted Signaling | KEGG: hsa04012 | LM |
| 167 | SPP1 Macrophage | Proliferating Macrophage | EREG | EGFR_ERBB2 | 3.87E-06 | 0 | EREG_EGFR_ERBB2 | EREG - (EGFR+ERBB2) | EGF | Secreted Signaling | KEGG: hsa04012 | LM |
| 168 | C9 | SPP1 Macrophage | EREG | EGFR_ERBB2 | 1.33E-05 | 0 | EREG_EGFR_ERBB2 | EREG - (EGFR+ERBB2) | EGF | Secreted Signaling | KEGG: hsa04012 | LM |
| 169 | SPP1 Macrophage | SPP1 Macrophage | EREG | EGFR_ERBB2 | 3.59E-05 | 0 | EREG_EGFR_ERBB2 | EREG - (EGFR+ERBB2) | EGF | Secreted Signaling | KEGG: hsa04012 | LM |
| 170 | C9 | C9 | EREG | ERBB4 | 4.44E-06 | 0 | EREG_ERBB4 | EREG - ERBB4 | EGF | Secreted Signaling | KEGG: hsa04012 | LM |
| 171 | Proliferating Macrophage | C9 | EREG | ERBB4 | 7.96E-07 | 0 | EREG_ERBB4 | EREG - ERBB4 | EGF | Secreted Signaling | KEGG: hsa04012 | LM |
| 172 | SPP1 Macrophage | C9 | EREG | ERBB4 | 1.20E-05 | 0 | EREG_ERBB4 | EREG - ERBB4 | EGF | Secreted Signaling | KEGG: hsa04012 | LM |
| 173 | C9 | FOLR2 Macrophage | EREG | ERBB4 | 1.76E-06 | 0 | EREG_ERBB4 | EREG - ERBB4 | EGF | Secreted Signaling | KEGG: hsa04012 | LM |
| 174 | SPP1 Macrophage | FOLR2 Macrophage | EREG | ERBB4 | 4.75E-06 | 0 | EREG_ERBB4 | EREG - ERBB4 | EGF | Secreted Signaling | KEGG: hsa04012 | LM |
| 175 | C9 | Proliferating Macrophage | EREG | ERBB4 | 2.52E-07 | 0 | EREG_ERBB4 | EREG - ERBB4 | EGF | Secreted Signaling | KEGG: hsa04012 | LM |
| 176 | Proliferating Macrophage | Proliferating Macrophage | EREG | ERBB4 | 4.52E-08 | 0.04 | EREG_ERBB4 | EREG - ERBB4 | EGF | Secreted Signaling | KEGG: hsa04012 | LM |
| 177 | SPP1 Macrophage | Proliferating Macrophage | EREG | ERBB4 | 6.81E-07 | 0 | EREG_ERBB4 | EREG - ERBB4 | EGF | Secreted Signaling | KEGG: hsa04012 | LM |
| 178 | C9 | SPP1 Macrophage | EREG | ERBB4 | 2.48E-06 | 0 | EREG_ERBB4 | EREG - ERBB4 | EGF | Secreted Signaling | KEGG: hsa04012 | LM |
| 179 | SPP1 Macrophage | SPP1 Macrophage | EREG | ERBB4 | 6.71E-06 | 0 | EREG_ERBB4 | EREG - ERBB4 | EGF | Secreted Signaling | KEGG: hsa04012 | LM |
| 180 | C9 | C9 | EREG | ERBB2_ERBB4 | 9.24E-06 | 0 | EREG_ERBB2_ERBB4 | EREG - (ERBB2+ERBB4) | EGF | Secreted Signaling | KEGG: hsa04012 | LM |
| 181 | Proliferating Macrophage | C9 | EREG | ERBB2_ERBB4 | 1.66E-06 | 0 | EREG_ERBB2_ERBB4 | EREG - (ERBB2+ERBB4) | EGF | Secreted Signaling | KEGG: hsa04012 | LM |
| 182 | SPP1 Macrophage | C9 | EREG | ERBB2_ERBB4 | 2.49E-05 | 0 | EREG_ERBB2_ERBB4 | EREG - (ERBB2+ERBB4) | EGF | Secreted Signaling | KEGG: hsa04012 | LM |
| 183 | C9 | FOLR2 Macrophage | EREG | ERBB2_ERBB4 | 5.16E-06 | 0 | EREG_ERBB2_ERBB4 | EREG - (ERBB2+ERBB4) | EGF | Secreted Signaling | KEGG: hsa04012 | LM |
| 184 | SPP1 Macrophage | FOLR2 Macrophage | EREG | ERBB2_ERBB4 | 1.39E-05 | 0 | EREG_ERBB2_ERBB4 | EREG - (ERBB2+ERBB4) | EGF | Secreted Signaling | KEGG: hsa04012 | LM |
| 185 | C9 | Proliferating Macrophage | EREG | ERBB2_ERBB4 | 7.42E-07 | 0 | EREG_ERBB2_ERBB4 | EREG - (ERBB2+ERBB4) | EGF | Secreted Signaling | KEGG: hsa04012 | LM |
| 186 | Proliferating Macrophage | Proliferating Macrophage | EREG | ERBB2_ERBB4 | 1.33E-07 | 0.04 | EREG_ERBB2_ERBB4 | EREG - (ERBB2+ERBB4) | EGF | Secreted Signaling | KEGG: hsa04012 | LM |
| 187 | SPP1 Macrophage | Proliferating Macrophage | EREG | ERBB2_ERBB4 | 2.00E-06 | 0 | EREG_ERBB2_ERBB4 | EREG - (ERBB2+ERBB4) | EGF | Secreted Signaling | KEGG: hsa04012 | LM |
| 188 | C9 | SPP1 Macrophage | EREG | ERBB2_ERBB4 | 7.01E-06 | 0 | EREG_ERBB2_ERBB4 | EREG - (ERBB2+ERBB4) | EGF | Secreted Signaling | KEGG: hsa04012 | LM |
| 189 | SPP1 Macrophage | SPP1 Macrophage | EREG | ERBB2_ERBB4 | 1.89E-05 | 0 | EREG_ERBB2_ERBB4 | EREG - (ERBB2+ERBB4) | EGF | Secreted Signaling | KEGG: hsa04012 | LM |
| 190 | C9 | C9 | NRG1 | ERBB3 | 1.59E-05 | 0 | NRG1_ERBB3 | NRG1 - ERBB3 | NRG | Secreted Signaling | KEGG: hsa04012 | LM |
| 191 | FOLR2 Macrophage | C9 | NRG1 | ERBB3 | 1.67E-06 | 0 | NRG1_ERBB3 | NRG1 - ERBB3 | NRG | Secreted Signaling | KEGG: hsa04012 | LM |
| 192 | Proliferating Macrophage | C9 | NRG1 | ERBB3 | 2.26E-07 | 0 | NRG1_ERBB3 | NRG1 - ERBB3 | NRG | Secreted Signaling | KEGG: hsa04012 | LM |
| 193 | SPP1 Macrophage | C9 | NRG1 | ERBB3 | 2.23E-06 | 0 | NRG1_ERBB3 | NRG1 - ERBB3 | NRG | Secreted Signaling | KEGG: hsa04012 | LM |
| 194 | C9 | FOLR2 Macrophage | NRG1 | ERBB3 | 1.12E-06 | 0 | NRG1_ERBB3 | NRG1 - ERBB3 | NRG | Secreted Signaling | KEGG: hsa04012 | LM |
| 195 | C9 | Proliferating Macrophage | NRG1 | ERBB3 | 1.45E-07 | 0 | NRG1_ERBB3 | NRG1 - ERBB3 | NRG | Secreted Signaling | KEGG: hsa04012 | LM |
| 196 | C9 | SPP1 Macrophage | NRG1 | ERBB3 | 1.44E-06 | 0 | NRG1_ERBB3 | NRG1 - ERBB3 | NRG | Secreted Signaling | KEGG: hsa04012 | LM |
| 197 | C9 | C9 | NRG1 | ERBB2_ERBB3 | 1.20E-05 | 0 | NRG1_ERBB2_ERBB3 | NRG1 - (ERBB2+ERBB3) | NRG | Secreted Signaling | KEGG: hsa04012 | LM |
| 198 | FOLR2 Macrophage | C9 | NRG1 | ERBB2_ERBB3 | 1.26E-06 | 0 | NRG1_ERBB2_ERBB3 | NRG1 - (ERBB2+ERBB3) | NRG | Secreted Signaling | KEGG: hsa04012 | LM |
| 199 | Proliferating Macrophage | C9 | NRG1 | ERBB2_ERBB3 | 1.70E-07 | 0 | NRG1_ERBB2_ERBB3 | NRG1 - (ERBB2+ERBB3) | NRG | Secreted Signaling | KEGG: hsa04012 | LM |
| 200 | SPP1 Macrophage | C9 | NRG1 | ERBB2_ERBB3 | 1.68E-06 | 0 | NRG1_ERBB2_ERBB3 | NRG1 - (ERBB2+ERBB3) | NRG | Secreted Signaling | KEGG: hsa04012 | LM |
| 201 | C9 | FOLR2 Macrophage | NRG1 | ERBB2_ERBB3 | 2.82E-06 | 0 | NRG1_ERBB2_ERBB3 | NRG1 - (ERBB2+ERBB3) | NRG | Secreted Signaling | KEGG: hsa04012 | LM |
| 202 | C9 | Proliferating Macrophage | NRG1 | ERBB2_ERBB3 | 3.85E-07 | 0 | NRG1_ERBB2_ERBB3 | NRG1 - (ERBB2+ERBB3) | NRG | Secreted Signaling | KEGG: hsa04012 | LM |
| 203 | Proliferating Macrophage | Proliferating Macrophage | NRG1 | ERBB2_ERBB3 | 5.46E-09 | 0.02 | NRG1_ERBB2_ERBB3 | NRG1 - (ERBB2+ERBB3) | NRG | Secreted Signaling | KEGG: hsa04012 | LM |
| 204 | C9 | SPP1 Macrophage | NRG1 | ERBB2_ERBB3 | 3.66E-06 | 0 | NRG1_ERBB2_ERBB3 | NRG1 - (ERBB2+ERBB3) | NRG | Secreted Signaling | KEGG: hsa04012 | LM |
| 205 | C9 | C9 | NRG1 | ERBB4 | 2.09E-06 | 0 | NRG1_ERBB4 | NRG1 - ERBB4 | NRG | Secreted Signaling | KEGG: hsa04012 | LM |
| 206 | FOLR2 Macrophage | C9 | NRG1 | ERBB4 | 2.19E-07 | 0 | NRG1_ERBB4 | NRG1 - ERBB4 | NRG | Secreted Signaling | KEGG: hsa04012 | LM |
| 207 | Proliferating Macrophage | C9 | NRG1 | ERBB4 | 2.96E-08 | 0 | NRG1_ERBB4 | NRG1 - ERBB4 | NRG | Secreted Signaling | KEGG: hsa04012 | LM |
| 208 | SPP1 Macrophage | C9 | NRG1 | ERBB4 | 2.92E-07 | 0 | NRG1_ERBB4 | NRG1 - ERBB4 | NRG | Secreted Signaling | KEGG: hsa04012 | LM |
| 209 | C9 | FOLR2 Macrophage | NRG1 | ERBB4 | 8.29E-07 | 0 | NRG1_ERBB4 | NRG1 - ERBB4 | NRG | Secreted Signaling | KEGG: hsa04012 | LM |
| 210 | C9 | Proliferating Macrophage | NRG1 | ERBB4 | 1.19E-07 | 0 | NRG1_ERBB4 | NRG1 - ERBB4 | NRG | Secreted Signaling | KEGG: hsa04012 | LM |
| 211 | C9 | SPP1 Macrophage | NRG1 | ERBB4 | 1.17E-06 | 0 | NRG1_ERBB4 | NRG1 - ERBB4 | NRG | Secreted Signaling | KEGG: hsa04012 | LM |
| 212 | C9 | C9 | NRG1 | ERBB2_ERBB4 | 4.35E-06 | 0 | NRG1_ERBB2_ERBB4 | NRG1 - (ERBB2+ERBB4) | NRG | Secreted Signaling | KEGG: hsa04012 | LM |
| 213 | FOLR2 Macrophage | C9 | NRG1 | ERBB2_ERBB4 | 4.55E-07 | 0 | NRG1_ERBB2_ERBB4 | NRG1 - (ERBB2+ERBB4) | NRG | Secreted Signaling | KEGG: hsa04012 | LM |
| 214 | Proliferating Macrophage | C9 | NRG1 | ERBB2_ERBB4 | 6.17E-08 | 0 | NRG1_ERBB2_ERBB4 | NRG1 - (ERBB2+ERBB4) | NRG | Secreted Signaling | KEGG: hsa04012 | LM |
| 215 | SPP1 Macrophage | C9 | NRG1 | ERBB2_ERBB4 | 6.08E-07 | 0 | NRG1_ERBB2_ERBB4 | NRG1 - (ERBB2+ERBB4) | NRG | Secreted Signaling | KEGG: hsa04012 | LM |
| 216 | C9 | FOLR2 Macrophage | NRG1 | ERBB2_ERBB4 | 2.43E-06 | 0 | NRG1_ERBB2_ERBB4 | NRG1 - (ERBB2+ERBB4) | NRG | Secreted Signaling | KEGG: hsa04012 | LM |
| 217 | C9 | Proliferating Macrophage | NRG1 | ERBB2_ERBB4 | 3.49E-07 | 0 | NRG1_ERBB2_ERBB4 | NRG1 - (ERBB2+ERBB4) | NRG | Secreted Signaling | KEGG: hsa04012 | LM |
| 218 | Proliferating Macrophage | Proliferating Macrophage | NRG1 | ERBB2_ERBB4 | 4.94E-09 | 0.01 | NRG1_ERBB2_ERBB4 | NRG1 - (ERBB2+ERBB4) | NRG | Secreted Signaling | KEGG: hsa04012 | LM |
| 219 | C9 | SPP1 Macrophage | NRG1 | ERBB2_ERBB4 | 3.30E-06 | 0 | NRG1_ERBB2_ERBB4 | NRG1 - (ERBB2+ERBB4) | NRG | Secreted Signaling | KEGG: hsa04012 | LM |
| 220 | C9 | C9 | FGF1 | FGFR1 | 2.06E-08 | 0 | FGF1_FGFR1 | FGF1 - FGFR1 | FGF | Secreted Signaling | PMC: 4393358 | LM |
| 221 | Proliferating Macrophage | C9 | FGF1 | FGFR1 | 3.02E-09 | 0 | FGF1_FGFR1 | FGF1 - FGFR1 | FGF | Secreted Signaling | PMC: 4393358 | LM |
| 222 | SPP1 Macrophage | C9 | FGF1 | FGFR1 | 2.69E-08 | 0 | FGF1_FGFR1 | FGF1 - FGFR1 | FGF | Secreted Signaling | PMC: 4393358 | LM |
| 223 | C9 | FOLR2 Macrophage | FGF1 | FGFR1 | 1.97E-08 | 0 | FGF1_FGFR1 | FGF1 - FGFR1 | FGF | Secreted Signaling | PMC: 4393358 | LM |
| 224 | C9 | Proliferating Macrophage | FGF1 | FGFR1 | 2.81E-09 | 0 | FGF1_FGFR1 | FGF1 - FGFR1 | FGF | Secreted Signaling | PMC: 4393358 | LM |
| 225 | SPP1 Macrophage | Proliferating Macrophage | FGF1 | FGFR1 | 3.66E-09 | 0 | FGF1_FGFR1 | FGF1 - FGFR1 | FGF | Secreted Signaling | PMC: 4393358 | LM |
| 226 | C9 | SPP1 Macrophage | FGF1 | FGFR1 | 2.91E-08 | 0 | FGF1_FGFR1 | FGF1 - FGFR1 | FGF | Secreted Signaling | PMC: 4393358 | LM |
| 227 | Proliferating Macrophage | SPP1 Macrophage | FGF1 | FGFR1 | 4.25E-09 | 0.04 | FGF1_FGFR1 | FGF1 - FGFR1 | FGF | Secreted Signaling | PMC: 4393358 | LM |
| 228 | SPP1 Macrophage | SPP1 Macrophage | FGF1 | FGFR1 | 3.78E-08 | 0 | FGF1_FGFR1 | FGF1 - FGFR1 | FGF | Secreted Signaling | PMC: 4393358 | LM |
| 229 | C9 | C9 | FGF1 | FGFR2 | 1.53E-08 | 0 | FGF1_FGFR2 | FGF1 - FGFR2 | FGF | Secreted Signaling | PMC: 4393358 | LM |
| 230 | Proliferating Macrophage | C9 | FGF1 | FGFR2 | 2.25E-09 | 0 | FGF1_FGFR2 | FGF1 - FGFR2 | FGF | Secreted Signaling | PMC: 4393358 | LM |
| 231 | SPP1 Macrophage | C9 | FGF1 | FGFR2 | 2.00E-08 | 0 | FGF1_FGFR2 | FGF1 - FGFR2 | FGF | Secreted Signaling | PMC: 4393358 | LM |
| 232 | C9 | FOLR2 Macrophage | FGF1 | FGFR2 | 6.15E-09 | 0 | FGF1_FGFR2 | FGF1 - FGFR2 | FGF | Secreted Signaling | PMC: 4393358 | LM |
| 233 | C9 | Proliferating Macrophage | FGF1 | FGFR2 | 1.28E-09 | 0 | FGF1_FGFR2 | FGF1 - FGFR2 | FGF | Secreted Signaling | PMC: 4393358 | LM |
| 234 | SPP1 Macrophage | Proliferating Macrophage | FGF1 | FGFR2 | 1.67E-09 | 0 | FGF1_FGFR2 | FGF1 - FGFR2 | FGF | Secreted Signaling | PMC: 4393358 | LM |
| 235 | C9 | SPP1 Macrophage | FGF1 | FGFR2 | 1.42E-08 | 0 | FGF1_FGFR2 | FGF1 - FGFR2 | FGF | Secreted Signaling | PMC: 4393358 | LM |
| 236 | Proliferating Macrophage | SPP1 Macrophage | FGF1 | FGFR2 | 2.08E-09 | 0.01 | FGF1_FGFR2 | FGF1 - FGFR2 | FGF | Secreted Signaling | PMC: 4393358 | LM |
| 237 | SPP1 Macrophage | SPP1 Macrophage | FGF1 | FGFR2 | 1.85E-08 | 0 | FGF1_FGFR2 | FGF1 - FGFR2 | FGF | Secreted Signaling | PMC: 4393358 | LM |
| 238 | C9 | C9 | FGF1 | FGFR3 | 1.94E-08 | 0 | FGF1_FGFR3 | FGF1 - FGFR3 | FGF | Secreted Signaling | PMC: 4393358 | LM |
| 239 | FOLR2 Macrophage | C9 | FGF1 | FGFR3 | 2.37E-09 | 0 | FGF1_FGFR3 | FGF1 - FGFR3 | FGF | Secreted Signaling | PMC: 4393358 | LM |
| 240 | Proliferating Macrophage | C9 | FGF1 | FGFR3 | 2.84E-09 | 0 | FGF1_FGFR3 | FGF1 - FGFR3 | FGF | Secreted Signaling | PMC: 4393358 | LM |
| 241 | SPP1 Macrophage | C9 | FGF1 | FGFR3 | 2.52E-08 | 0 | FGF1_FGFR3 | FGF1 - FGFR3 | FGF | Secreted Signaling | PMC: 4393358 | LM |
| 242 | C9 | FOLR2 Macrophage | FGF1 | FGFR3 | 1.68E-09 | 0 | FGF1_FGFR3 | FGF1 - FGFR3 | FGF | Secreted Signaling | PMC: 4393358 | LM |
| 243 | C9 | Proliferating Macrophage | FGF1 | FGFR3 | 5.60E-10 | 0 | FGF1_FGFR3 | FGF1 - FGFR3 | FGF | Secreted Signaling | PMC: 4393358 | LM |
| 244 | Proliferating Macrophage | Proliferating Macrophage | FGF1 | FGFR3 | 8.20E-11 | 0.03 | FGF1_FGFR3 | FGF1 - FGFR3 | FGF | Secreted Signaling | PMC: 4393358 | LM |
| 245 | SPP1 Macrophage | Proliferating Macrophage | FGF1 | FGFR3 | 7.29E-10 | 0 | FGF1_FGFR3 | FGF1 - FGFR3 | FGF | Secreted Signaling | PMC: 4393358 | LM |
| 246 | C9 | SPP1 Macrophage | FGF1 | FGFR3 | 4.71E-09 | 0 | FGF1_FGFR3 | FGF1 - FGFR3 | FGF | Secreted Signaling | PMC: 4393358 | LM |
| 247 | Proliferating Macrophage | SPP1 Macrophage | FGF1 | FGFR3 | 6.89E-10 | 0.01 | FGF1_FGFR3 | FGF1 - FGFR3 | FGF | Secreted Signaling | PMC: 4393358 | LM |
| 248 | SPP1 Macrophage | SPP1 Macrophage | FGF1 | FGFR3 | 6.13E-09 | 0 | FGF1_FGFR3 | FGF1 - FGFR3 | FGF | Secreted Signaling | PMC: 4393358 | LM |
| 249 | C9 | C9 | FGF1 | FGFR4 | 1.78E-08 | 0 | FGF1_FGFR4 | FGF1 - FGFR4 | FGF | Secreted Signaling | PMC: 4393358 | LM |
| 250 | FOLR2 Macrophage | C9 | FGF1 | FGFR4 | 2.18E-09 | 0 | FGF1_FGFR4 | FGF1 - FGFR4 | FGF | Secreted Signaling | PMC: 4393358 | LM |
| 251 | Proliferating Macrophage | C9 | FGF1 | FGFR4 | 2.61E-09 | 0 | FGF1_FGFR4 | FGF1 - FGFR4 | FGF | Secreted Signaling | PMC: 4393358 | LM |
| 252 | SPP1 Macrophage | C9 | FGF1 | FGFR4 | 2.32E-08 | 0 | FGF1_FGFR4 | FGF1 - FGFR4 | FGF | Secreted Signaling | PMC: 4393358 | LM |
| 253 | C9 | Proliferating Macrophage | FGF1 | FGFR4 | 5.45E-10 | 0 | FGF1_FGFR4 | FGF1 - FGFR4 | FGF | Secreted Signaling | PMC: 4393358 | LM |
| 254 | Proliferating Macrophage | Proliferating Macrophage | FGF1 | FGFR4 | 7.98E-11 | 0.03 | FGF1_FGFR4 | FGF1 - FGFR4 | FGF | Secreted Signaling | PMC: 4393358 | LM |
| 255 | SPP1 Macrophage | Proliferating Macrophage | FGF1 | FGFR4 | 7.10E-10 | 0 | FGF1_FGFR4 | FGF1 - FGFR4 | FGF | Secreted Signaling | PMC: 4393358 | LM |
| 256 | C9 | SPP1 Macrophage | FGF1 | FGFR4 | 4.40E-09 | 0 | FGF1_FGFR4 | FGF1 - FGFR4 | FGF | Secreted Signaling | PMC: 4393358 | LM |
| 257 | Proliferating Macrophage | SPP1 Macrophage | FGF1 | FGFR4 | 6.44E-10 | 0 | FGF1_FGFR4 | FGF1 - FGFR4 | FGF | Secreted Signaling | PMC: 4393358 | LM |
| 258 | SPP1 Macrophage | SPP1 Macrophage | FGF1 | FGFR4 | 5.72E-09 | 0 | FGF1_FGFR4 | FGF1 - FGFR4 | FGF | Secreted Signaling | PMC: 4393358 | LM |
| 259 | C9 | C9 | FGF2 | FGFR1 | 2.48E-08 | 0 | FGF2_FGFR1 | FGF2 - FGFR1 | FGF | Secreted Signaling | PMC: 4393358 | LM |
| 260 | FOLR2 Macrophage | C9 | FGF2 | FGFR1 | 4.42E-09 | 0 | FGF2_FGFR1 | FGF2 - FGFR1 | FGF | Secreted Signaling | PMC: 4393358 | LM |
| 261 | Proliferating Macrophage | C9 | FGF2 | FGFR1 | 7.40E-10 | 0 | FGF2_FGFR1 | FGF2 - FGFR1 | FGF | Secreted Signaling | PMC: 4393358 | LM |
| 262 | SPP1 Macrophage | C9 | FGF2 | FGFR1 | 7.12E-09 | 0 | FGF2_FGFR1 | FGF2 - FGFR1 | FGF | Secreted Signaling | PMC: 4393358 | LM |
| 263 | C9 | FOLR2 Macrophage | FGF2 | FGFR1 | 2.36E-08 | 0 | FGF2_FGFR1 | FGF2 - FGFR1 | FGF | Secreted Signaling | PMC: 4393358 | LM |
| 264 | C9 | Proliferating Macrophage | FGF2 | FGFR1 | 3.37E-09 | 0 | FGF2_FGFR1 | FGF2 - FGFR1 | FGF | Secreted Signaling | PMC: 4393358 | LM |
| 265 | C9 | SPP1 Macrophage | FGF2 | FGFR1 | 3.49E-08 | 0 | FGF2_FGFR1 | FGF2 - FGFR1 | FGF | Secreted Signaling | PMC: 4393358 | LM |
| 266 | Proliferating Macrophage | SPP1 Macrophage | FGF2 | FGFR1 | 1.04E-09 | 0.03 | FGF2_FGFR1 | FGF2 - FGFR1 | FGF | Secreted Signaling | PMC: 4393358 | LM |
| 267 | C9 | C9 | FGF2 | FGFR2 | 1.84E-08 | 0 | FGF2_FGFR2 | FGF2 - FGFR2 | FGF | Secreted Signaling | PMC: 4393358 | LM |
| 268 | FOLR2 Macrophage | C9 | FGF2 | FGFR2 | 3.29E-09 | 0 | FGF2_FGFR2 | FGF2 - FGFR2 | FGF | Secreted Signaling | PMC: 4393358 | LM |
| 269 | Proliferating Macrophage | C9 | FGF2 | FGFR2 | 5.51E-10 | 0 | FGF2_FGFR2 | FGF2 - FGFR2 | FGF | Secreted Signaling | PMC: 4393358 | LM |
| 270 | SPP1 Macrophage | C9 | FGF2 | FGFR2 | 5.30E-09 | 0 | FGF2_FGFR2 | FGF2 - FGFR2 | FGF | Secreted Signaling | PMC: 4393358 | LM |
| 271 | C9 | FOLR2 Macrophage | FGF2 | FGFR2 | 7.38E-09 | 0 | FGF2_FGFR2 | FGF2 - FGFR2 | FGF | Secreted Signaling | PMC: 4393358 | LM |
| 272 | C9 | Proliferating Macrophage | FGF2 | FGFR2 | 1.53E-09 | 0 | FGF2_FGFR2 | FGF2 - FGFR2 | FGF | Secreted Signaling | PMC: 4393358 | LM |
| 273 | SPP1 Macrophage | Proliferating Macrophage | FGF2 | FGFR2 | 4.41E-10 | 0.04 | FGF2_FGFR2 | FGF2 - FGFR2 | FGF | Secreted Signaling | PMC: 4393358 | LM |
| 274 | C9 | SPP1 Macrophage | FGF2 | FGFR2 | 1.71E-08 | 0 | FGF2_FGFR2 | FGF2 - FGFR2 | FGF | Secreted Signaling | PMC: 4393358 | LM |
| 275 | Proliferating Macrophage | SPP1 Macrophage | FGF2 | FGFR2 | 5.11E-10 | 0 | FGF2_FGFR2 | FGF2 - FGFR2 | FGF | Secreted Signaling | PMC: 4393358 | LM |
| 276 | SPP1 Macrophage | SPP1 Macrophage | FGF2 | FGFR2 | 4.91E-09 | 0 | FGF2_FGFR2 | FGF2 - FGFR2 | FGF | Secreted Signaling | PMC: 4393358 | LM |
| 277 | C9 | C9 | FGF2 | FGFR3 | 2.32E-08 | 0 | FGF2_FGFR3 | FGF2 - FGFR3 | FGF | Secreted Signaling | PMC: 4393358 | LM |
| 278 | FOLR2 Macrophage | C9 | FGF2 | FGFR3 | 4.15E-09 | 0 | FGF2_FGFR3 | FGF2 - FGFR3 | FGF | Secreted Signaling | PMC: 4393358 | LM |
| 279 | Proliferating Macrophage | C9 | FGF2 | FGFR3 | 6.94E-10 | 0 | FGF2_FGFR3 | FGF2 - FGFR3 | FGF | Secreted Signaling | PMC: 4393358 | LM |
| 280 | SPP1 Macrophage | C9 | FGF2 | FGFR3 | 6.68E-09 | 0 | FGF2_FGFR3 | FGF2 - FGFR3 | FGF | Secreted Signaling | PMC: 4393358 | LM |
| 281 | C9 | FOLR2 Macrophage | FGF2 | FGFR3 | 2.01E-09 | 0 | FGF2_FGFR3 | FGF2 - FGFR3 | FGF | Secreted Signaling | PMC: 4393358 | LM |
| 282 | C9 | Proliferating Macrophage | FGF2 | FGFR3 | 6.71E-10 | 0 | FGF2_FGFR3 | FGF2 - FGFR3 | FGF | Secreted Signaling | PMC: 4393358 | LM |
| 283 | Proliferating Macrophage | Proliferating Macrophage | FGF2 | FGFR3 | 2.01E-11 | 0.02 | FGF2_FGFR3 | FGF2 - FGFR3 | FGF | Secreted Signaling | PMC: 4393358 | LM |
| 284 | SPP1 Macrophage | Proliferating Macrophage | FGF2 | FGFR3 | 1.93E-10 | 0.01 | FGF2_FGFR3 | FGF2 - FGFR3 | FGF | Secreted Signaling | PMC: 4393358 | LM |
| 285 | C9 | SPP1 Macrophage | FGF2 | FGFR3 | 5.64E-09 | 0 | FGF2_FGFR3 | FGF2 - FGFR3 | FGF | Secreted Signaling | PMC: 4393358 | LM |
| 286 | Proliferating Macrophage | SPP1 Macrophage | FGF2 | FGFR3 | 1.69E-10 | 0 | FGF2_FGFR3 | FGF2 - FGFR3 | FGF | Secreted Signaling | PMC: 4393358 | LM |
| 287 | SPP1 Macrophage | SPP1 Macrophage | FGF2 | FGFR3 | 1.62E-09 | 0 | FGF2_FGFR3 | FGF2 - FGFR3 | FGF | Secreted Signaling | PMC: 4393358 | LM |
| 288 | C9 | C9 | FGF2 | FGFR4 | 2.14E-08 | 0 | FGF2_FGFR4 | FGF2 - FGFR4 | FGF | Secreted Signaling | PMC: 4393358 | LM |
| 289 | FOLR2 Macrophage | C9 | FGF2 | FGFR4 | 3.82E-09 | 0 | FGF2_FGFR4 | FGF2 - FGFR4 | FGF | Secreted Signaling | PMC: 4393358 | LM |
| 290 | Proliferating Macrophage | C9 | FGF2 | FGFR4 | 6.39E-10 | 0 | FGF2_FGFR4 | FGF2 - FGFR4 | FGF | Secreted Signaling | PMC: 4393358 | LM |
| 291 | SPP1 Macrophage | C9 | FGF2 | FGFR4 | 6.14E-09 | 0 | FGF2_FGFR4 | FGF2 - FGFR4 | FGF | Secreted Signaling | PMC: 4393358 | LM |
| 292 | C9 | FOLR2 Macrophage | FGF2 | FGFR4 | 1.43E-09 | 0 | FGF2_FGFR4 | FGF2 - FGFR4 | FGF | Secreted Signaling | PMC: 4393358 | LM |
| 293 | C9 | Proliferating Macrophage | FGF2 | FGFR4 | 6.54E-10 | 0 | FGF2_FGFR4 | FGF2 - FGFR4 | FGF | Secreted Signaling | PMC: 4393358 | LM |
| 294 | Proliferating Macrophage | Proliferating Macrophage | FGF2 | FGFR4 | 1.96E-11 | 0.02 | FGF2_FGFR4 | FGF2 - FGFR4 | FGF | Secreted Signaling | PMC: 4393358 | LM |
| 295 | SPP1 Macrophage | Proliferating Macrophage | FGF2 | FGFR4 | 1.88E-10 | 0.01 | FGF2_FGFR4 | FGF2 - FGFR4 | FGF | Secreted Signaling | PMC: 4393358 | LM |
| 296 | C9 | SPP1 Macrophage | FGF2 | FGFR4 | 5.27E-09 | 0 | FGF2_FGFR4 | FGF2 - FGFR4 | FGF | Secreted Signaling | PMC: 4393358 | LM |
| 297 | Proliferating Macrophage | SPP1 Macrophage | FGF2 | FGFR4 | 1.58E-10 | 0 | FGF2_FGFR4 | FGF2 - FGFR4 | FGF | Secreted Signaling | PMC: 4393358 | LM |
| 298 | SPP1 Macrophage | SPP1 Macrophage | FGF2 | FGFR4 | 1.52E-09 | 0 | FGF2_FGFR4 | FGF2 - FGFR4 | FGF | Secreted Signaling | PMC: 4393358 | LM |
| 299 | C9 | C9 | FGF5 | FGFR1 | 2.34E-08 | 0 | FGF5_FGFR1 | FGF5 - FGFR1 | FGF | Secreted Signaling | PMC: 4393358 | LM |
