# Supplementary material for: Single‐Cell Transcriptomic Analysis Identifies a Novel OLR1 + SLC7A7 + Liver‐Enriched Metastatic Subset With Immunometabolic Rewiring in Pancreatic Cancer
Source: Cancer Med. 2025 Nov 2;14(21):e71345. doi: 10.1002/cam4.71345 (PMC12579899; doi:10.1002/cam4.71345)
Supplement: Supplementary file 4 — Table S3: Top 100 differentially expressed genes identified by pseudotemporal trajectory analysis. [file CAM4-14-e71345-s004.docx]

| **Supplementary Table 3. Top 100 differentially expressed genes identified by pseudotemporal trajectory analysis** | | | | |
| --- | --- | --- | --- | --- |
|  | **pval** | **qval** | **gene** | **num_cells_expressed** |
| MT-ND3 | 4.94749612536816e-163 | 1.5201832831526e-160 | MT-ND3 | 1509 |
| PCSK1N | 1.94553765198033e-144 | 4.54321952490446e-142 | PCSK1N | 375 |
| LGALS1 | 3.19302247856114e-143 | 7.16956354993843e-141 | LGALS1 | 723 |
| TIMP3 | 8.90248090568077e-130 | 1.77179602934197e-127 | TIMP3 | 121 |
| TFF3 | 0 | 0 | TFF3 | 952 |
| TFF2 | 1.99152022760844e-200 | 8.3046393491272e-198 | TFF2 | 965 |
| TFF1 | 0 | 0 | TFF1 | 1068 |
| SLPI | 4.0180142161991e-134 | 8.27900011558953e-132 | SLPI | 1360 |
| UBE2C | 5.85377124085798e-144 | 1.33146687678424e-141 | UBE2C | 176 |
| TGM2 | 0 | 0 | TGM2 | 646 |
| UBE2S | 5.8192659193422e-148 | 1.41553643487999e-145 | UBE2S | 735 |
| KLK6 | 4.46855103120264e-244 | 2.44569383626509e-241 | KLK6 | 414 |
| FTL | 2.52563593463562e-149 | 6.60208772525497e-147 | FTL | 1536 |
| APOE | 3.3113522110039e-170 | 1.1152888966062e-167 | APOE | 357 |
| LYPD3 | 1.05321563618106e-241 | 5.42529960355147e-239 | LYPD3 | 222 |
| CEACAM5 | 0 | 0 | CEACAM5 | 720 |
| CEACAM6 | 2.36425654369711e-242 | 1.25477542746398e-239 | CEACAM6 | 1199 |
| COMP | 7.77289205494619e-280 | 4.69429073966646e-277 | COMP | 114 |
| C3 | 0 | 0 | C3 | 485 |
| GATA6-AS1 | 6.57442289580577e-121 | 1.18705610924889e-118 | GATA6-AS1 | 382 |
| VMP1 | 7.65779825381129e-136 | 1.61588769418375e-133 | VMP1 | 1420 |
| ABCC3 | 4.65453722415598e-140 | 1.00641438202306e-137 | ABCC3 | 1225 |
| KRT17 | 0 | 0 | KRT17 | 604 |
| MT2A | 2.46640678903679e-234 | 1.1999069028664e-231 | MT2A | 816 |
| TMC5 | 1.07853948867512e-164 | 3.4980630749363e-162 | TMC5 | 1205 |
| THBS1 | 0 | 0 | THBS1 | 248 |
| OLFM4 | 1.53810701961778e-310 | 1.22447301552663e-307 | OLFM4 | 403 |
| MDM2 | 8.44709290353245e-165 | 2.79136575683901e-162 | MDM2 | 670 |
| CPM | 3.89970904007656e-290 | 2.52961126399633e-287 | CPM | 259 |
| LYZ | 0 | 0 | LYZ | 1289 |
| AC020656.1 | 6.25487107635018e-321 | 5.47738961342856e-318 | AC020656.1 | 721 |
| ATP5F1B | 5.95684991123638e-176 | 2.12914835398763e-173 | ATP5F1B | 1239 |
| KRT7 | 0 | 0 | KRT7 | 1329 |
| KRT18 | 8.5120971631508e-128 | 1.6382513155541e-125 | KRT18 | 1501 |
| IGFBP6 | 1.93218327478946e-118 | 3.45308753823088e-116 | IGFBP6 | 294 |
| TUBA1B | 2.02252230900276e-148 | 5.0603508171249e-146 | TUBA1B | 1010 |
| MGST1 | 1.39095563704625e-131 | 2.80013758933656e-129 | MGST1 | 1095 |
| LDHB | 3.50201352117494e-297 | 2.45337059239432e-294 | LDHB | 493 |
| MGP | 0 | 0 | MGP | 149 |
| RIMKLB | 7.45468208379204e-162 | 2.25105693130231e-159 | RIMKLB | 307 |
| YBX3 | 2.02474463036186e-127 | 3.85449754958236e-125 | YBX3 | 1171 |
| CD9 | 7.72471160503049e-129 | 1.50322887833893e-126 | CD9 | 1312 |
| GAPDH | 7.68764616827863e-174 | 2.64002813708298e-171 | GAPDH | 1502 |
| APOA1 | 2.83242907006716e-142 | 6.20089534164452e-140 | APOA1 | 90 |
| SYTL2 | 1.29802356346407e-175 | 4.54671693810195e-173 | SYTL2 | 861 |
| FOLR1 | 5.22846860840732e-150 | 1.40879075704071e-147 | FOLR1 | 169 |
| FTH1 | 3.32915809426343e-200 | 1.35597383402162e-197 | FTH1 | 1540 |
| MUC5AC | 8.03561260841677e-286 | 5.02627568656469e-283 | MUC5AC | 842 |
| CTSD | 3.80096094102664e-127 | 7.08191807671709e-125 | CTSD | 1349 |
| H19 | 7.43013500334884e-304 | 5.65788628037616e-301 | H19 | 82 |
| CD81 | 1.21464523554049e-134 | 2.53253531610193e-132 | CD81 | 853 |
| PRAP1 | 0 | 0 | PRAP1 | 446 |
| IFITM3 | 1.63910534452828e-122 | 2.99034281292378e-120 | IFITM3 | 1331 |
| DMBT1 | 0 | 0 | DMBT1 | 462 |
| SNCG | 1.16351770685299e-312 | 9.70373767515393e-310 | SNCG | 257 |
| ADIRF | 2.63966775566125e-133 | 5.37571407821524e-131 | ADIRF | 749 |
| PLAU | 2.03715515702542e-157 | 6.0472432915497e-155 | PLAU | 340 |
| SRGN | 2.50234664713729e-144 | 5.76659199710034e-142 | SRGN | 292 |
| VIM | 4.68587475269586e-154 | 1.32368403901154e-151 | VIM | 432 |
| AKR1C2 | 0 | 0 | AKR1C2 | 268 |
| TNC | 1.93026996885052e-204 | 8.45168705861201e-202 | TNC | 122 |
| ANXA1 | 0 | 0 | ANXA1 | 1143 |
| LYPD2 | 7.20345565762087e-202 | 3.07710542408712e-199 | LYPD2 | 269 |
| TRBC2 | 2.46299142843967e-154 | 7.07161178322826e-152 | TRBC2 | 225 |
| CAV1 | 3.23942981654869e-215 | 1.45475317453933e-212 | CAV1 | 397 |
| FAM3C | 2.94359288341686e-225 | 1.35668646737271e-222 | FAM3C | 1045 |
| CYP3A5 | 3.0339900499393e-164 | 9.6613275881158e-162 | CYP3A5 | 1103 |
| PEG10 | 3.41551092304782e-149 | 8.79694975092051e-147 | PEG10 | 204 |
| UPK3B | 1.22436966916697e-156 | 3.57393506429837e-154 | UPK3B | 201 |
| IGFBP3 | 2.92738617553295e-153 | 8.1381335679816e-151 | IGFBP3 | 349 |
| CD24 | 1.14386761288784e-149 | 3.0354086927451e-147 | CD24 | 1281 |
| PGC | 4.29929247654513e-163 | 1.34460372203949e-160 | PGC | 185 |
| HMGA1 | 6.63894063223899e-146 | 1.57127575990586e-143 | HMGA1 | 1055 |
| DUSP1 | 1.73848694801669e-176 | 6.34330425157589e-174 | DUSP1 | 1053 |
| IQGAP2 | 3.84529136624e-143 | 8.52486493523132e-141 | IQGAP2 | 768 |
| FYB1 | 8.54731887711158e-117 | 1.49697742813732e-114 | FYB1 | 190 |
| IGFBP7 | 1.33957675931798e-182 | 5.10029290493373e-180 | IGFBP7 | 402 |
| EREG | 2.63423588504805e-259 | 1.48825829970102e-256 | EREG | 296 |
| AREG | 7.38013191256327e-236 | 3.69301800904666e-233 | AREG | 764 |
| CLDN18 | 2.00749184803452e-226 | 9.50248979093962e-224 | CLDN18 | 917 |
| DCBLD2 | 9.85720494197662e-137 | 2.10535472382657e-134 | DCBLD2 | 502 |
| FN1 | 0 | 0 | FN1 | 286 |
| SULT1C2 | 0 | 0 | SULT1C2 | 779 |
| LINC00342 | 0 | 0 | LINC00342 | 792 |
| ANKRD36C | 9.77838388239682e-302 | 7.13577563817908e-299 | ANKRD36C | 894 |
| REG1A | 3.25582084642099e-147 | 7.81129401427633e-145 | REG1A | 223 |
| CAPN8 | 3.44294006593607e-127 | 6.48383358223702e-125 | CAPN8 | 1040 |
| CTSE | 2.71818981602456e-293 | 1.83101447837901e-290 | CTSE | 1235 |
| PIGR | 1.04944069015846e-126 | 1.93472676288792e-124 | PIGR | 628 |
| MIR205HG | 0 | 0 | MIR205HG | 123 |
| ELF3 | 1.50219913865988e-129 | 2.95612536117856e-127 | ELF3 | 1451 |
| ATP1B1 | 7.51561993775868e-149 | 1.9076603998537e-146 | ATP1B1 | 1434 |
| ITLN1 | 3.01442852812208e-181 | 1.12329151577723e-178 | ITLN1 | 80 |
| MUC1 | 1.46682918261326e-266 | 8.56334876809622e-264 | MUC1 | 1466 |
| S100A9 | 5.47530842697634e-190 | 2.13099003977919e-187 | S100A9 | 282 |
| S100A4 | 1.1162891358511e-151 | 3.05479498832753e-149 | S100A4 | 1077 |
| S100A2 | 0 | 0 | S100A2 | 474 |
| REG4 | 1.96358077272589e-192 | 7.81594401216391e-190 | REG4 | 567 |
| GUCA2B | 2.21978283581985e-148 | 5.47567275866884e-146 | GUCA2B | 83 |
| STMN1 | 3.37535052702509e-118 | 5.9713019323553e-116 | STMN1 | 603 |
