# Supplementary material for: Single‐Cell Transcriptomic Analysis Identifies a Novel OLR1 + SLC7A7 + Liver‐Enriched Metastatic Subset With Immunometabolic Rewiring in Pancreatic Cancer
Source: Cancer Med. 2025 Nov 2;14(21):e71345. doi: 10.1002/cam4.71345 (PMC12579899; doi:10.1002/cam4.71345)
Supplement: Supplementary file 5 — Table S4: The score of key metabolic pathways. [file CAM4-14-e71345-s003.docx]

| **Supplementary Table 4. The score of key metabolic pathways.** | | | | | | | |
| --- | --- | --- | --- | --- | --- | --- | --- |
| **Name** | **p_val** | **avg_diff** | **p_val_adj** | **cluster** | **direction** | **methods** | **target.gene** |
| Fatty acid degradation | 1.79333530130667e-260 | 0.0109269980001245 | 1.52433500611067e-258 | C2 | up | AUCell | ACAT2, ACAT1, ACAA1, ACAA2, HADHB, HADH, HADHA, EHHADH, ECHS1, ACOX3, ACOX1, ACADS, ACADM, ACADL, ACADSB, ACADVL, GCDH, ACSL6, ACSL4, ACSL1, ACSL5, ACSL3, ACSBG1, ACSBG2, CPT1A, CPT1B, CPT1C, CPT2, ECI1, ECI2, CYP4A11, CYP4A22, ADH1A, ADH1B, ADH1C, ADH7, ADH4, ADH5, ADH6, ALDH2, ALDH3A2, ALDH1B1, ALDH7A1, ALDH9A1 |
|  | 0.966066693468049 | -0.000127052 | 1 | C6 | down | AUCell |  |
|  | 7.377762871031e-28 | 0.00666459547403121 | 6.27109844037635e-26 | C9 | up | AUCell |  |
| Fatty acid elongation | 8.5234318266984e-122 | 0.00856151491127901 | 7.24491705269364e-120 | C2 | up | AUCell | ACAA2, HADHB, HADH, HADHA, ECHS1, MECR, PPT1, PPT2, ABHD17A, ABHD17B, ABHD17C, ELOVL1, ELOVL2, ELOVL3, ELOVL4, ELOVL5, ELOVL6, ELOVL7, HSD17B12, HACD2, HACD1, HACD4, HACD3, TECR, ACOT4, ACOT2, ACOT1, ACOT7, THEM4, THEM5 |
|  | 4.8749025679199e-21 | 0.00410137241305755 | 4.14366718273191e-19 | C6 | up | AUCell |  |
|  | 2.82075994523228e-22 | 0.00830321092486343 | 2.39764595344744e-20 | C9 | up | AUCell |  |
| Fatty acid biosynthesis | 6.09823154853841e-25 | 0.00396445350329486 | 5.18349681625765e-23 | C2 | up | AUCell | ACACA, ACACB, MCAT, FASN, OXSM, OLAH, ACSL6, ACSL4, ACSL1, ACSL5, ACSL3, ACSBG1, ACSBG2 |
|  | 0.451360074746302 | -0.002303084 | 1 | C6 | down | AUCell |  |
|  | 1.11535514868692e-05 | 0.00326169402779539 | 0.000948051876383881 | C9 | up | AUCell |  |
| Glycolysis - Gluconeogenesis | 2.9934543007812e-123 | 0.00978337766182381 | 2.54443615566402e-121 | C2 | up | AUCell | HK3, HK1, HK2, HKDC1, GCK, GPI, PFKM, PFKP, PFKL, FBP1, FBP2, ALDOC, ALDOA, ALDOB, TPI1, GAPDH, GAPDHS, PGK2, PGK1, PGAM1, PGAM2, PGAM4, ENO3, ENO2, ENO1, ENO4, PKM, PKLR, PDHA1, PDHB, DLAT, DLD, LDHAL6A, LDHAL6B, LDHA, LDHB, LDHC, ADH1A, ADH1B, ADH1C, ADH7, ADH4, ADH5, ADH6, AKR1A1, ALDH2, ALDH3A2, ALDH1B1, ALDH7A1, ALDH9A1, ALDH3B1, ALDH3B2, ALDH1A3, ALDH3A1, ACSS1, ACSS2, GALM, PGM1, PGM2, G6PC, G6PC2, G6PC3, ADPGK, BPGM, MINPP1, PCK1, PCK2 |
|  | 3.04768484473634e-68 | 0.00980298205287823 | 2.59053211802589e-66 | C6 | up | AUCell |  |
|  | 8.4643814201044e-106 | 0.0204908624542636 | 7.19472420708874e-104 | C9 | up | AUCell |  |
